# Chemical genetics reveals cross-regulation of plant developmental signaling by the immune peptide-receptor pathway

**DOI:** 10.1126/sciadv.ads3718

**Published:** 2025-02-05

**Authors:** Arvid Herrmann, Krishna Mohan Sepuru, Pengfei Bai, Hitoshi Endo, Ayami Nakagawa, Shuhei Kusano, Asraa Ziadi, Hiroe Kato, Ayato Sato, Jun Liu, Libo Shan, Seisuke Kimura, Kenichiro Itami, Naoyuki Uchida, Shinya Hagihara, Keiko U. Torii

**Affiliations:** ^1^ Howard Hughes Medical Institute, University of Texas at Austin, Austin, TX 78712, USA.; ^2^ Department of Molecular Biosciences, University of Texas at Austin, Austin, TX 78712, USA.; ^3^ Institute of Transformative Bio-Molecules, Nagoya University, Nagoya, Aichi 464-8601, Japan.; ^4^ RIKEN Center for Sustainable Resource Science, Wako, Saitama 351-0198, Japan.; ^5^ Department of Biochemistry and Biophysics, Texas A&M University, College Station, TX 77843, USA.; ^6^ Faculty of Life Sciences and Center for Plant Sciences, Kyoto Sangyo University, Kamigamo-Motoyama, Kita-ku, Kyoto 603-8555, Japan.

## Abstract

Cells sense and integrate multiple signals to coordinate a response. A receptor-kinase signaling pathway for plant stomatal development shares components with the immunity pathway. The mechanism ensuring their signal specificities remains unclear. Using chemical genetics, here, we report the identification of a small molecule, kC9, that triggers excessive stomatal differentiation by inhibiting the canonical ERECTA pathway. kC9 binds to and inhibits the downstream mitogen-activated protein kinase MPK6, perturbing its substrate interaction. Notably, activation of immune signaling by a bacterial flagellin peptide nullified kC9’s effects on stomatal development. This cross-regulation depends on the immune receptor FLS2 (FLAGELLIN SENSING 2) and occurs even in the absence of kC9 if the ERECTA family receptor population becomes suboptimal. Proliferating stomatal lineage cells are vulnerable to this immune signal penetration. Our findings suggest that the signal specificity between development and immunity can be ensured by mitogen-activated protein kinase homeostasis, reflecting the availability of upstream receptors, thereby providing an unanticipated view on signal specificity.

## Teaser

A small compound uncovers the MAP kinase homeostasis–based signal specificity between plant development and immunity.

## INTRODUCTION

Living organisms must sense numerous environmental and endogenous cues and integrate information to grow and survive. For effectively allocating their resources, plants balance their growth and defense. As such, how the growth and defense signal transduction pathways cross-talk while maintaining specificities remains an important question. It is well established that plants use a battery of cell-surface receptors, known as receptor kinases (RKs), to perceive signals (*1*, *2*). Among them, those RKs with extracellular leucine-rich repeats (LRR-RKs) mediate developmental and environmental/immunity signals and elicit the responses accordingly (*3*–*6*). In the past decades, ligand binding, activation mechanisms, and downstream signal transduction pathways of LRR-RKs have been studied extensively (*6*–*8*). Increasing efforts have been made to dissect the mechanism of signal cross-talk between development and defense, which happens at multiple levels from the segregation of receptor complexes at the plasma membrane to the regulation of gene expressions (*9*, *10*). Still, understanding how each RK signaling pathway maintains signal specificity remains largely enigmatic.

The developmental patterning of stomata, cellular valves on the land plant epidermis for photosynthetic gas exchange and water control, is enforced by the ERECTA family of LRR-RKs, ERECTA, ERECTA-LIKE 1 (ERL1), and ERL2 (*11*). These receptors perceive a family of secreted peptides, EPIDERMAL PATTERNING FACTORs (EPFs)/EPF-LIKEs (EPFLs), to synergistically enforce proper stomatal patterning (*12*–*14*). Upon ligand binding, the activated ERECTA family receptors form a receptor complex with BRI1-ASSOCIATED KINASE 1 (BAK1)/SOMATIC EMBRYOGENESIS RECEPTOR KINASEs (SERKs) to transduce cellular signals (*15*). The LRR receptor-like protein TOO MANY MOUTHS (TMM) associates with ERECTA family receptors to discriminate different EPF/EPFL peptides in a context-specific manner (*16*–*20*). The signal is then transduced to a mitogen-activated protein kinase (MAPK) cascade, comprising MAPK kinase kinase (MAPKKK) YODA (also known as MAPKKK4), two redundant MAPK kinases (MAPKKs) MKK4 and MKK5, and two redundant MAPKs MPK3 and MPK6 (*21*–*23*). The activated MPK3 and MPK6 inhibit stomatal differentiation via direct binding to a basic-helix-loop-helix (bHLH) transcription factor, SCREAM (SCRM, also known as ICE1), and subsequent phosphorylation and degradation of bHLH heterodimers of SCRM and SPEECHLESS (SPCH) (*21*, *22*, *24*, *25*). The *SPCH* paralogs, *MUTE* and *FAMA*, act sequentially to promote the differentiation of stomatal precursor cells, meristemoids, and then guard mother cells (*26*). Recent genetic studies indicate that the receptor-like cytoplasmic kinases BRASSINOSTEROID SIGNALING KINASE 1 (BSK1) and BSK2 (*27*) act downstream of the ERECTA family, likely bridging the receptor complex to the MAPK cascade (*28*).

Increasing evidence show that whereas the upstream ligand-receptor pairs and downstream transcription factors of the LRR-RK pathways are unique, their intermediate signaling components are shared among the different pathways (*29*, *30*). The EPF-ERECTA family pathway largely shares co-receptor and downstream MAPK components with the well-established immunity pathway mediated by the FLAGELLIN SENSING 2 (FLS2) LRR-RK (*15*, *21*, *31*–*33*). FLS2 perceives the bacterial flagellin peptide flg22, forms an active receptor complex with BAK1, and parallelly activates two downstream MAPK cascades: one mediated by MEKK1, MKK1/2, and MPK4 to prevent autoimmunity and the other mediated by MAPKKK3/5, MKK4/5, and MPK3/6 that phosphorylate transcription factors (e.g., WRKY) for defense gene expression (*30*–*35*). MKK4/5 and MPK3/6 are shared by both ERECTA family and FLS2 pathways. How could each RK pathway maintain signal specificity while sharing the same components? Initially, stomatal development and immunity pathways were predicted to use distinct MAPKKKs, YODA and MAPKKK3/5, respectively (*23*, *36*), which may determine the signal specificity. It was reported that ERECTA family and FLS2 signaling pathways maintain specificity via antagonistic interactions between YODA (MAPKKK4) and MAPKKK3/5 (*37*). However, two more recent studies show that YODA (MAPKKK4), MAPKKK3, and MAPKKK5 have overlapping functions in stomatal development and immunity, thereby arguing against their mutually inhibitory roles (*38*, *39*). These contrasting observations may stem from complex genetic redundancy and pleiotropy/lethality in these mutants, which often hamper the dissection of intricate signaling pathways.

Chemical genetics offers an unbiased approach to investigate signal transduction pathways in cells to whole organisms through perturbations of biological targets (e.g., proteins) by small molecules (*40*–*42*). The controllable nature of chemical application, both in dose and timing, may enable disentanglement of signaling pathways. With this in mind, we performed a large-scale chemical genetics screen and identified a small compound, kC9 [hydroxy-2-naphthalenymethylphosphonic acid (HNMPA)], as a potent inducer of stomatal development by interfering with the EPF-ERECTA signaling pathway. Further mechanistic studies using available genetic mutant resources, chemical derivatization and structure-activity relationship analyses, and biochemical and biophysical binding assays backed up with docking modeling show that kC9 is an inhibitor of MAPK, MPK6. Intriguingly, we found that kC9’s effects in increasing stomatal development are fully nullified by the activation of the flg22-FLS2 signaling pathway. This unexpected signal cross-regulation of stomatal development by the immune signaling pathway occurs even in the absence of kC9, if the functional population of ERECTA family receptors becomes limited. Detailed time-course studies further uncovered the developmental window whereby stomatal lineage cells are most vulnerable to the flg22-FLS2 immune signal activation. Our findings suggest that the signal specificity between two distinct signaling pathways in development and immunity can be ensured when they are in a fully activable state. The work introduces an additional regulatory layer to the current understanding of signaling specificity, which has primarily emphasized pathway-specific scaffolds, protein-protein interactions, and cell-type specificity (*43*).

## RESULTS

### A small molecule promotes stomatal development by interfering with a canonical signaling pathway

**Fig. 1.**
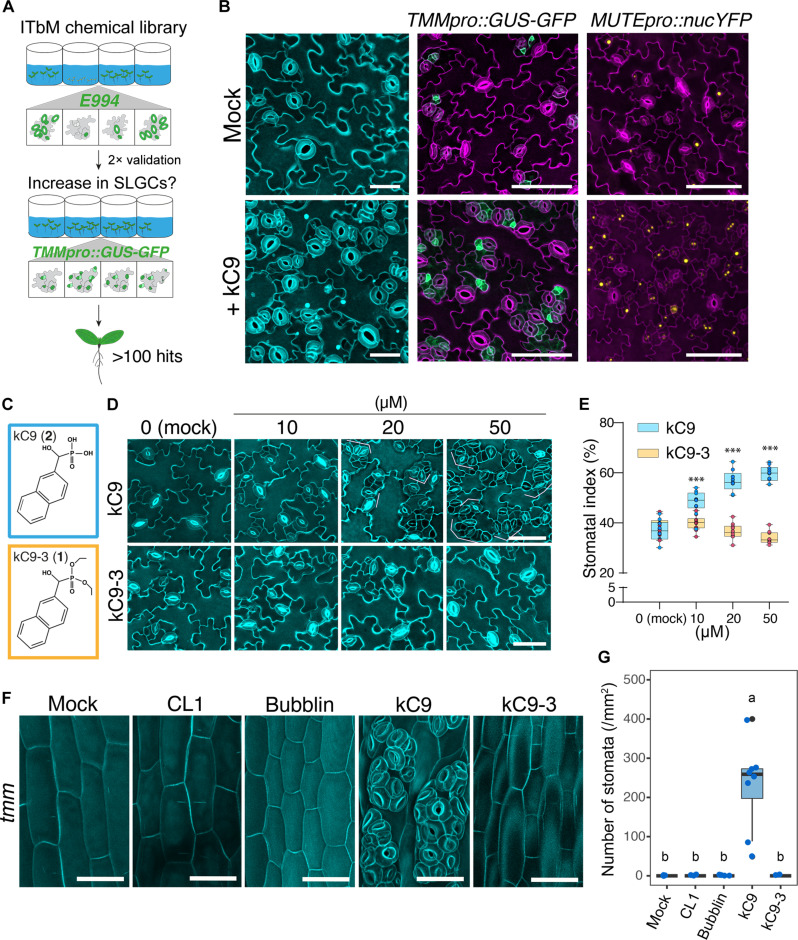
kC9 increases stomata formation by affecting the cell-cell signaling pathways. (**A**) Schematics of a phenotype-based screen using a synthetic compound library. Seedlings expressing the guard cell marker *E994* were screened for an increased number of stomata. The phenotype was validated by two different markers (*E994* and *TMMpro::GUS*-*GFP*). (**B**) Effects of kC9 on stomatal differentiation. Shown are representative images of cotyledon abaxial epidermis from 9-day-old WT (left), *TMMpro::GUS*-*GFP* (middle), and *MUTEpro::nucYFP* (right) seedlings treated with mock (top) or 50 μM kC9. Cell peripheries are visualized with propidium iodide. Scale bars, 40 μm (left) and 100 μm (middle and right). (**C**) Chemical structure of kC9 (top; compound **2**) and its nonbioactive analog kC9-3 (bottom, compound **1**). (**D**) Representative images of cotyledon abaxial seedling epidermis treated with kC9 or kC9-3 at 0 (mock), 10, 20, or 50 μM. Pink brackets, stomatal clusters. Scale bars, 50 μm. (**E**) Quantitative analysis of SI in (D). *n* = 10. Two-tailed Student’s unpaired *t* test was performed for kC9 versus kC9-3. Mock: not significant (NS), *P* = 0.4621529; 10 μM: ***P = 0.000298551; 20 μM: ***P = 1.2829 × 10^−9^; 50 μM: ***P = 7.292 × 10^−14^. (**F**) kC9 reverts the absence of stomata on *tmm* hypocotyls. Shown are representative images of hypocotyl epidermis of 9-day-old *tmm* treated with either mock, 25 μM CL1, 10 μM Bubblin, 50 μM kC9, or 50 μM kC9-3. Scale bars, 50 μm. See fig. S2 for schematic diagrams depicting the organ-specific regional effects of *tmm* mutation and their molecular underpinnings. (**G**) Quantitative analyses of stomatal density per 1 mm^2^ of hypocotyl epidermis from the *tmm* seedlings grown in each compound as indicated in (F). One-way analysis of variance (ANOVA) followed by Tukey’s post hoc analysis was performed. Letters (a or b) indicate groups that are statistically different. *n* = 8.

To identify novel chemical compounds with profound effects on stomatal development, we performed a phenotype-based screen using our pipeline of curated small molecules on Arabidopsis seedlings expressing a guard cell–specific green fluorescent protein (GFP) marker (Fig. 1A; see Materials and Methods). Among ~10,000 compounds screened, we identified HNMPA (from now referred to as kC9) as a potent inducer of stomatal differentiation, resulting in severe stomatal clustering (Fig. 1 and fig. S1). HNMPA is a known inhibitor of the human insulin receptor with weak/modest efficacy (*44*). However, its action or targets in plants are unknown. Consistent with the increase in the number of stomata (fig. S1, B and C), the number of cells expressing stomatal lineage markers *TMMpro::GUS*-*GFP* (Fig. 1B) and *MUTEpro::nucYFP* (Fig. 1B) is substantially increased. The effect of kC9 is dose dependent, significantly increasing the number of stomata as its concentration increases (Fig. 1, C to E; and fig. S1, B and C).

To characterize the structure-activity relationship of kC9, we subsequently synthesized a series of kC9 analogs and tested them for their bioactivity (Fig. 1, C to E; fig. S1, D and E; and document S1). kC9 (compound **2**) consists of two aryl rings (naphthalene) with a phosphonic acid through a hydroxymethylene linker (Fig. 1C and document S1). Through chemical synthesis, we replaced the side chains [kC9-3 (**1**), kC9-A (**10**), kC9-11 (**11**), and kC9-13 (**13**)] or added an additional naphthalene to its predecessor [kC9-8 (**6**)] [fig. S1; see document S1 for details of synthesis and nuclear magnetic resonance (NMR) analyses]. While kC9 can increase the stomatal index (SI) in a dose-dependent manner, kC9-3, in which all hydroxy groups in the phosphoric acid moiety are replaced by methoxyl groups, failed to increase the number of stomata (Fig. 1, C to E). Likewise, kC9-8 did not increase the number of stomata (fig. S1, D and E), whereas the remaining kC9 analogs exhibit bioactivity. Together, these results suggest that the sizes of aryl rings (kC9-8) and the phosphonic acid (kC9-3) are crucial for the bioactivity of kC9 to promote stomatal development.

We sought to test whether kC9 triggers stomatal development by affecting the canonical EPF-ERECTA family signaling pathway. For this purpose, we took advantage of the *tmm* mutant phenotypes. Whereas *tmm* cotyledons and leaves form stomatal clusters like the *erecta (er) erl1 erl2* triple mutant, *tmm* hypocotyls and stems are devoid of stomata (*11*, *45*). This is due to the role of TMM in safeguarding the inappropriate activation of ERECTA family RKs (*16*, *17*, *46*, *47*): In cotyledons and leaves, TMM forms a receptor complex with the ERECTA family and assists EPF1/2 ligand perception. In contrast, in hypocotyls and stems, TMM buffers EPFL4/5/6 peptides secreted from inner cell layers, which normally mediate other ERECTA family–dependent developmental processes, such as stem elongation and vascular patterning (*16*, *17*, *47*) (fig. S2). Because of this, in the absence of TMM, ERECTA family RKs become overly activated, resulting in a complete inhibition of stomatal differentiation (fig. S2). Genetically, loss-of-function mutations within the *EPF/EPFL*-*ERECTA* family genes can “bring back” stomatal differentiation on *tmm* hypocotyl epidermis (*11*, *16*, *17*). We therefore predicted that if kC9 acts on the EPF/EPFL-ERECTA signaling pathway, its application can revert the *tmm* hypocotyl phenotype. Intriguingly, kC9 application significantly increases the stomatal development, conferring severe stomatal clustering on stomata-forming cell files of *tmm* hypocotyls, whereas its inactive analog kC9-3 has no effects (Fig. 1, F and G). Unlike kC9, neither the previously reported small molecule CL1, which increases stomata (*48*), nor Bubblin [4-(4-bromophenyl)-2-pyridin-3-yl-1,3-thiazole], which triggers asymmetric division defects and stomatal cluster formation (*49*), can resume stomatal differentiation on *tmm* hypocotyls (Fig. 1, F and G). Therefore, we conclude that kC9 must act within the EPF/EPFL-ERECTA family signaling pathways.

### kC9 targets the stomatal MAPK cascade

**Fig. 2.**
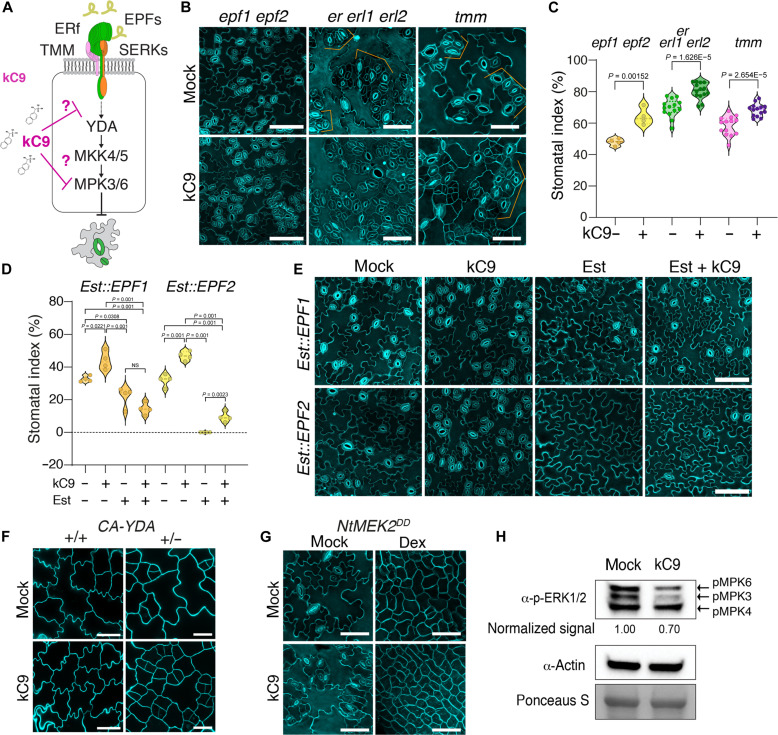
kC9 acts at the point of the MAPK. (**A**) Cartoon of kC9 action (red T-bars) on the stomata peptide-receptor signaling pathways. Arrow, activation. T-bar, inhibition. (**B**) Representative confocal images of the 7-day-old abaxial cotyledon epidermis from *epf1 epf2* (left), *er erl1 erl2* (middle), and *tmm* (right) seedlings treated with mock (top) or 50 μM kC9. Scale bars, 50 μm. (**C**) SI of *epf1 epf2* (*n* = 4, **P = 0.0015), *er105 erl1*-*2 erl2*-*1* (*n* = 14, ****P = 1.6256 × 10^−5^), and *tmm* (*n* = 14, ****P = 2.6540 × 10^−5^) seedlings treated with mock (top) or 50 μM kC9. Two-tailed unpaired Student’s *t* test was performed. (**D** and **E**) Induced overexpression of EPF1 or EPF2 counteracts the activity of kC9 on stomatal development. (D) SI of the 7-day-old abaxial epidermis from *Est::EPF1* and *Est::EPF2* seedlings treated with mock, 50 μM kC9, 5 μM estradiol (Est), or both. *n* = 4. One-way ANOVA followed by Tukey’s post hoc analysis was performed. For exact *P* values, see dataset S1. (E) Representative confocal images corresponding to (D). Scale bars, 50 μm. (**F**) kC9 treatment to constitutively active *YDA* (*CA*-*YDA*) seedlings*.* Note that *CA*-*YDA* homozygotes (+/+) exhibit a pavement cell–only phenotype, while heterozygous plants (+/-) show an increase in small stomatal lineage cells upon 50 μM kC9 treatment. (**G**) kC9 treatment to dexamethasone (Dex)–induced overexpression of NtMEK2^DD^, which is equivalent to MKK4/5 constitutive activation. (**H**) MAPK activation assays. Immunoblot analysis showing phosphorylated MAPKs (pMPK3/6) in WT seedlings treated with mock or 50 μM kC9 for 6 hours. Here, the blots are overexposed to visualize the basal level of MAPK activation. Actin was used as a control. Ponceau S staining was used to ensure equal loading of protein samples. Normalized signal intensities relative to mock treatment are indicated below the blot.

Our analyses of *tmm* mutant hypocotyls uncovered that kC9 potentially acts within the canonical stomatal cell-cell signaling pathway (Fig. 1, F and G). To locate the exact target of kC9, we used a chemical-genetic approach and treated different stomatal pathway mutants with kC9 (Fig. 2A and fig. S3). Single or higher-order mutants impaired in the peptide-receptor-signaling complex, *epf1 epf2*, *er erl1 erl2*, and *tmm*, as well as the *serk1 serk2 bak1* triple mutant, all display a significant increase in the SI upon kC9 application compared to their respective mock controls (Fig. 2, B and C; and fig. S3, A and B). Among the stomatal transcription factor mutants, kC9 treatment had no effects on *spch* mutant epidermis (fig. S3C). Whereas kC9 vastly increased the arrested meristemoids in *mute* and the guard mother cell (GMC) tumors in *fama*, no stomatal differentiation occurred (fig. S3C), indicating that kC9 cannot promote stomatal differentiation in the absence of *SPCH/MUTE/FAMA*. Combined, the results suggest that kC9 affects downstream of the peptide-receptor signaling module but upstream of the bHLH master transcription factors. As reported previously (*14*), induced overexpression of *EPF1* (*Est::EPF1*) or *EPF2* (*Est::EPF2*) severely inhibits stomatal development. Notably, the kC9 application has minimal effects on the inhibition of stomatal development when *EPF1* and *EPF2* are overexpressed (Fig. 2, D and E), indicating that activated EPF-ERECTA family signaling can counteract the effects of kC9.

We further dissected the MAPK pathway affected by kC9. It is known that YDA MAPKKK and MKK4/5 MAPKK relay phosphorylation signals to MPK3/6 to enforce stomatal development (*26*). As such, a constitutive activation of YDA (CA-YDA), as well as that of MKK4/5 by a tobacco (*Nicotiana tabacum*) ortholog of Arabidopsis MKK4/5, NtMEK2^DD^, strongly activates MPK3/6 and confers astomatous epidermis (*21*, *23*). The treatment of kC9 exerts no effects on this “pavement cell–only” phenotype of CA-YDA and induced NtMEK2^DD^ overexpression (Fig. 2, F and G), indicating that the constitutive activation of the YDA-MKK4/5-MPK3/6 pathway prevents kC9 from exerting its effects. Consistent with the notion that CA-YDA acts in a dose-dependent manner because of a weaker MAPK activation (*21*, *22*), heterozygous CA-YDA plants responded to kC9 treatment with increased small, stomatal lineage cells (Fig. 2F and fig. S3D). On the other hand, weak cosuppression lines of *YDA* showed diminished efficacy of kC9 (fig. S3, E and F). A single loss-of-function *mpk3* and *mpk6* mutant, however, still responds to kC9 treatment, consistent with their functional redundancy (fig. S3, G and H).

These mechanistic dissections of kC9 action within the EPF-ERECTA family signaling pathway narrow down kC9’s in vivo targets to MAPKs. To directly address this possibility, we performed established, immunoblot-based MAPK activation assays using an anti-active MAPK antibody (*50*). We optimized the immunoblotting and exposures to reliably detect relative signal differences. As shown in Fig. 2H, treatment of Arabidopsis wild-type (WT) seedlings under a stationary condition with 50 μM kC9 notably reduced the activated population of MPK3 and MPK6 in vivo. Together, we conclude that kC9 triggers excessive stomatal differentiation via inhibition of the MAPKs, whereas a constitutive activation of the MAPK cascade incapacitates the action of kC9.

### kC9 directly binds to MPK6 and inhibits kinase activity

**Fig. 3.**
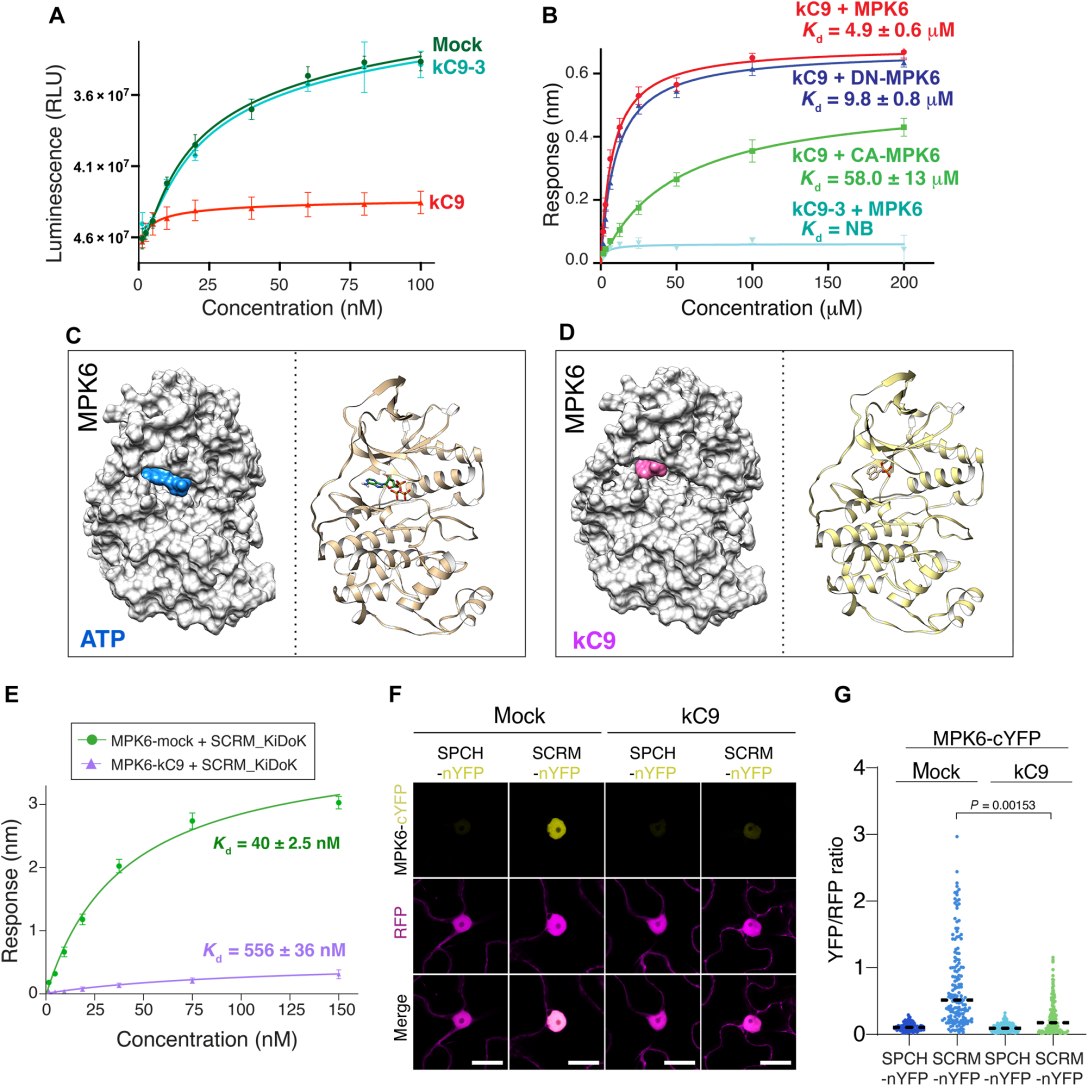
kC9 inhibits MPK6 activity and interferes with the MPK6-SCRM association. (**A**) Kinase activity assays for MPK6 in the presence of mock (dark green), 10 μM kC9-3 (cyan), and 10 μM kC9 (red). A decrease in luminescence (RLU, relative light unit) indicates reduced MAPK activity. The *y* axis has been inverted for clarity. (**B**) Quantitative analysis of kC9 binding kinetics to MPK6, inactive MPK6_K92M_K93R_ (DN-MPK6), and constitutively active MPK6_D218G_E222A_ (CA-MPK6). The data represent the means ± SD and are representative of two independent experiments. (**C** and **D**) Structural docking models of the known MPK6 structure (PDB: 6TDL) with ATP [(C); blue] and kC9 [(D); magenta] sharing a similar binding site. (**E**) Quantitative analysis of MPK6 with SCRM_KiDoK peptide interactions in the presence and absence of kC9. *K*_d_ values are indicated. Data are presented as the means ± SD and representative of three independent experiments. (**F** and **G**) rBiFC analysis of interaction between MPK6 and SCRM as well as SPCH (negative control) in the absence (mock) or presence of kC9 in *N. benthamiana* epidermis leaves. Representative confocal images (F). Scale bars, 20 μm. Quantitative analysis (G). MPK6-cYFP + SPCH-nYFP, *n* = 126; MPK6-cYFP + SCRM-nYFP, *n* = 158. One-way ANOVA followed by Tukey’s post hoc analysis was performed. For exact *P* values, see dataset S1.

To decipher the molecular action of kC9, we first investigated whether kC9 inhibits MAPK activity via direct binding. For these purposes, we chose MPK6 for its available structural information (*24*). As shown in Fig. 3A, in vitro kinase activity assays show that kC9 (**2**) inhibits the activity of recombinant MPK6 in a dose-dependent manner. In contrast, the inactive analog kC9-3 (**1**) has no such effect. Next, we examined the in vitro direct quantitative binding of kC9 (**2**) and recombinant MPK6 using biolayer interferometry (BLI) and isothermal titration calorimetry (ITC) assays. kC9 (**2**) showed binding to MPK6 with a dissociation constant (*K*_d_) of 4.9 ± 0.6 μM. In contrast, the inactive analog kC9-3 (**1**) did not exhibit any binding (Fig. 3B, fig. S4, and tables S1 and S2). Because the activation of the MAPK cascade incapacitates kC9 from increasing the number of stomata (Fig. 2 and fig. S2), we predicted that the constitutive activation of MPK6 may hinder kC9 binding. A constitutively active CA-MPK6, MPK6_D218G_E222A_, showed severely reduced binding to kC9 with a *K*_d_ value of 58.0 ± 13 μM (Fig. 3B, fig. S4, and tables S1 and S2). In addition, kinase-inactive mutations DN-MPK6, MPK6_K92M_K93R_, slightly decreased the kC9 binding (fig. S4 and tables S1 and S2). Furthermore, we examined whether kC9 binds to MKK5, one of the upstream MAPKKs of MPK6 in stomatal development (*21*). Our ITC assays did not detect any kC9 binding to MKK5 in vitro (fig. S4 and table S1), thus molecularly delineating the site of kC9 action within the canonical stomatal development MAPK cascade.

The reduced binding of kC9 to CA-MPK6 (and, to some extent, to DN-MPK6) implies that activation-loop dynamics play a prominent role in kC9 binding, suggestive of its binding to the adenosine 5′-triphosphate (ATP)–binding pocket of MPK6. To address this, we took both experimental and docking simulation approaches. First, we observed that kC9 can effectively compete with ATP for binding to MPK6 (fig. S4). The ATP binding affinity decreased ~2.5-fold in the presence of kC9 using ITC (table S2), indicating that either kC9 competes directly with ATP at the binding site or both share a similar binding pocket. Second, we conducted docking modeling studies to identify the specific binding pockets for kC9 and ATP. Briefly, we used high ambiguity–driven biomolecular docking (HADDOCK)–based calculations to guide the docking process, incorporating binding site information from Swiss Dock as ambiguous interaction restraints (*51*, *52*). The resulting structural models indicate that kC9 fits to the ATP-binding pocket and that the binding interfaces involve a combination of hydrophobic and charged residues (Fig. 3, C and D; and fig. S4, B and C). Because of its single phosphate group, kC9 can form only two hydrogen bonds with MPK6 compared to five between ATP and MPK6 (fig. S4C). This weak binding mode may be reflected by the nature of kC9 not to affect stomatal development when the MAPK cascade is activated (Fig. 2 and fig. S3). In contrast, the inactive analog kC9-3 (**1**) as well as kC9-8 (**6**) docking with MPK6 generated very few high-energy structures, and these ligands are transiently fit at the narrow ATP-binding pocket between N and C lobes because of their bulky nature compared to kC9 (fig. S4, D and E, and table S3). The docking results suggest that kC9 is a more favorable ligand compared to its inactive counterparts at the ATP-binding pocket. On the basis of these findings, we conclude that kC9 directly inhibits MAPK (MPK6) by competitively binding to its ATP-binding pocket and that kC9’s binding is perturbed when MPK6 is activated.

### kC9 interferes with MPK6-SCRM binding

Many small-molecule inhibitors of MAPKs have been identified and used pharmacologically. Having identified kC9 as a direct inhibitor of MPK6, we sought to explore its selectivity and effectiveness for stomatal development. It has been shown that MPK6 is directly recruited to the SPCH-SCRM heterodimers via a bipartite MAPK binding motif within SCRM, named a KiDoK motif (*24*). The mutation within this motif disrupts MPK6-SCRM interaction, thereby stabilizing the SPCH-SCRM heterodimers and resulting in a “stomata-only” epidermis (*24*, *25*). The predicted MPK6-KiDoK binding interface (*24*) partially overlaps with the kC9-binding grove. We thus postulated that kC9 may perturb MPK6-KiDoK (hence MPK6-SCRM) association in addition to inhibiting the kinase activity. To test this hypothesis, we first performed BLI and ITC (Fig. 3E; fig. S4, L and M; tables S1 and S2). Preincubating kC9 with MPK6 decreased the binding affinity of MPK6 and the SCRM KiDoK domain by 80 to 100% for both BLI and ITC (*K*_d_ values increased from 40.1 ± 1.2 to 856.5 ± 16.0 nM in BLI and from 43.4 ± 2.1 and 1403 ± 40 nM in ITC with or without preincubation with kC9, respectively) (Fig. 3E; fig. S4, L and M; and tables S1 and S2).

We further examined whether kC9 perturbs the MPK-SCRM association in planta by ratiometric bimolecular fluorescence complementation (rBiFC) assays. In this setup, MPK6 and SCRM (or control SPCH), each fused with a complementary half yellow fluorescent protein (YFP), along with a full-length red fluorescent protein (RFP) for normalization are located on the same plasmid, which were coexpressed in *Nicotiana benthamiana* leaves (see Materials and Methods). A strong YFP signal was reconstituted in MPK6-cYFP and SCRM-nYFP, confirming their direct association (Fig. 3F). Notably, kC9 treatment significantly diminished the YFP signals (Fig. 3, F and G). SPCH-nYFP served as a negative control as it has been shown that MPK6 does not directly associate with SPCH (*24*) (Fig. 3, F and G). Collectively, these results affirm that kC9 binding not only attenuates the kinase activity of MPK6 but also disrupts the association with its direct substrate SCRM. This dual mode of kC9 may underpin the propensity of kC9 to induce excessive stomatal cluster formation.

### kC9 confers modest reduction in immune response

**Fig. 4.**
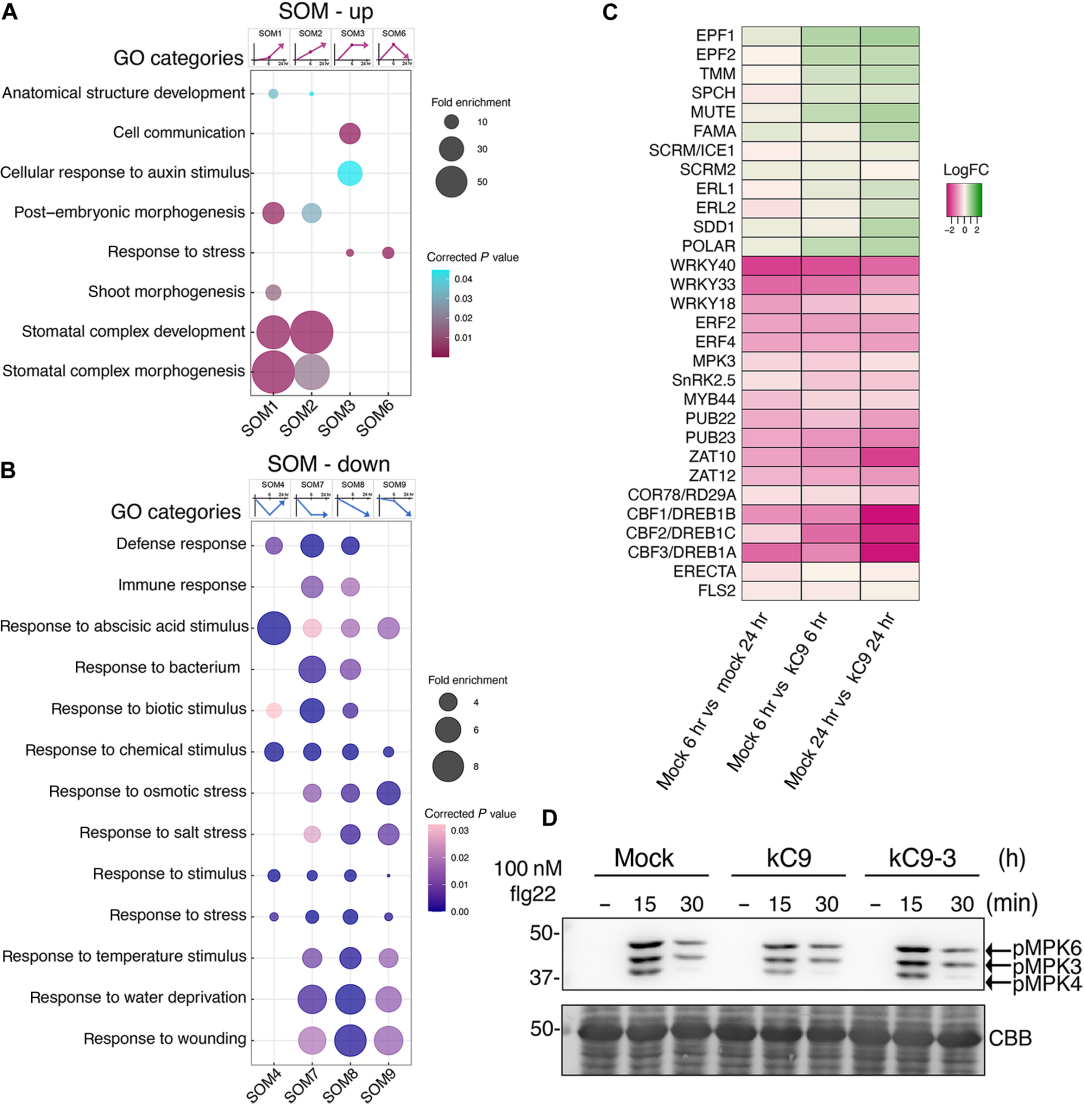
kC9 attenuates stress and immunity pathways. (**A** and **B**) SOM clustering and GO enrichment of up-regulated (A) and down-regulated (B) DEG categories. The gene expression trajectories of each SOM category are indicated as a diagram above the corresponding column of bubble plots. Selected major GO terms are visualized by bubble plots. Fold enrichment and corrected *P* values of each cluster are visualized according to the bubble size and color scale, respectively. Number of genes in each SOM category: SOM1, 125; SOM2, 100; SOM3, 93; SOM4, 112; SOM6, 42; SOM7, 127; SOM8, 174; SOM9, 140. SOM cluster 5 is absent. See also data S2. (**C**) Heatmap showing the expression changes of the selected genes in up-regulated (A) and down-regulated (B) SOM clusters. Those from the up-regulated SOMs include stomatal development genes, and those from the down-regulated SOMs include biotic (e.g., immunity) and broad abiotic (cold-, drought-, abscisic acid–, or ethylene-mediated) pathways. The heatmaps present the mean log_2_(fold change) values relative to the corresponding levels in the respective mock treatment. See also data S2. hr, hours. (**D**) kC9 attenuates the MAPK activation in immune signaling. Top, immunoblot by an anti-pERK antibody of seedlings treated with 100 nM flg22 for 0, 15, and 30 min in mock (left), kC9 (middle), or inactive kC9-3 (right). Bottom, total proteins stained with Coomassie brilliant blue (CBB). The locations of phosphorylated MAPKs are indicated by the arrows at the right. The molecular mass marker size is indicated at the left.

We have demonstrated that kC9 exaggerates the number of stomata by directly inhibiting MPK6 activity as well as perturbing the recruitment of its downstream target SCRM, the latter of which likely contributes to the high propensity of kC9 to affect stomatal development (Figs. 1 to 3 and figs. S1, S3, and S4). It is well established that MPK6 mediates diverse abiotic (environmental) and biotic stress signaling pathways, including drought, osmotic, salt, and temperature stresses as well as immunity/defense responses (*53*–*56*). To gain a comprehensive view of signaling pathways influenced by kC9, we took an unbiased approach and characterized transcriptome dynamics in response to kC9 treatment. Specifically, WT seedlings were subjected to mock or kC9 treatment for 6 and 24 hours followed by RNA sequencing (RNA-seq) (Fig. 4, A to C, and dataset S2; see Materials and Methods). Here, the seedlings were grown under a continuous light condition to minimize the circadian effects on gene expression. We identified 500 and 641 differentially expressed genes (DEGs) between mock versus kC9 treatment for 6 and 24 hours, respectively, with false discovery rate (FDR) <0.01. Of these DEGs, 228 genes overlapped between both 6 and 24 hours. Thus, 913 genes were detected as DEGs between mock and kC9 (fig. S5).

To categorize the dynamic response patterns of the DEGs, we next performed 3 by 3 self-organizing map (SOM) clustering on the basis of the log_2_(fold change) values of the three time points, including time 0, where theoretically, no difference exists between mock and kC9. The SOM clusters were then classified into up- or down-regulated groups, after which gene expression [Gene Ontology (GO)] categories overrepresented within each SOM were identified (see Materials and Methods). Consistent with kC9’s effects, GO categories including “stomatal complex development” and “stomatal complex morphogenesis” are highly enriched in the up-regulated SOM clusters, SOM1 (slowly up) and SOM2 (constantly up) (Fig. 4A). Other developmental processes including “shoot morphogenesis,” “embryonic morphogenesis,” and “anatomical structure development” are also detected in these SOMs. SOM3 (up and stay steady) includes “cell communication” (Fig. 4A). Consistently, stomatal regulatory genes are uniformly up-regulated by kC9 treatment (Fig. 4C).

Notably, down-regulated SOM clusters are overwhelmingly dominated by abiotic and biotic stress response categories (Fig. 4B). Various biotic stress response categories including “defense response” and “response to biotic stimulus” are enriched in SOM4 (rapidly down and then back up), SOM7 (rapidly down and stay down), and SOM8 (constantly down). “Immune response” and “response to bacterium” are enriched in SOM7 and SOM8. Abiotic stress response categories are overrepresented in SOM7, SOM8, and SOM9 (slowly down and then down), including “response to osmotic stress,” “response to salt stress,” “response to temperature stress,” “response to water deprivation,” and “response to wounding.” General stress response categories, such as “response to stimulus,” “response to stress,” and “response to abscisic acid stimulus,” are enriched across all four down-regulated SOMs (Fig. 4B). kC9 significantly repressed the expression of key transcription factor genes, such as *CBF1/DREB1B*, *CBF2/DREB1C*, and *CBF3/DREB1A*, for temperature/drought stress response as well as *WRKY33* and *WRKY40* for immunity response (*57*–*59*)(Fig. 4C). Whereas the overrepresented GO categories for down-regulated SOM clusters overwhelmingly include stress and immune responses, the differential expression of individual genes is not very substantial. This may be due to the developmental time point of choice (3-day-old seedlings), which may have been too young and thus not optimal for the detection of stress/immunity molecular signatures.

To test whether kC9 application attenuates the activity of MAPKs in the context of stress signaling, we turned our focus on a well-established immune signaling pathway (*32*). As previously demonstrated (*50*), the application of the flg22 peptide to Arabidopsis seedlings elicits rapid phosphorylation and activation of MPKs (MPK3/6/4) within 15 min (Fig. 4D). Preincubation with kC9 slightly diminishes the flg22-induced phosphorylation, suggesting that kC9 can attenuate the activation of immune signaling by flg22 (Fig. 4D). Collectively, our results suggest that kC9’s potency to inhibit MPK6 extends beyond the context of stomatal development to diverse stress responses, including immune signaling.

### Activation of immune signaling nullifies kC9 from inducing stomatal development

**Fig. 5.**
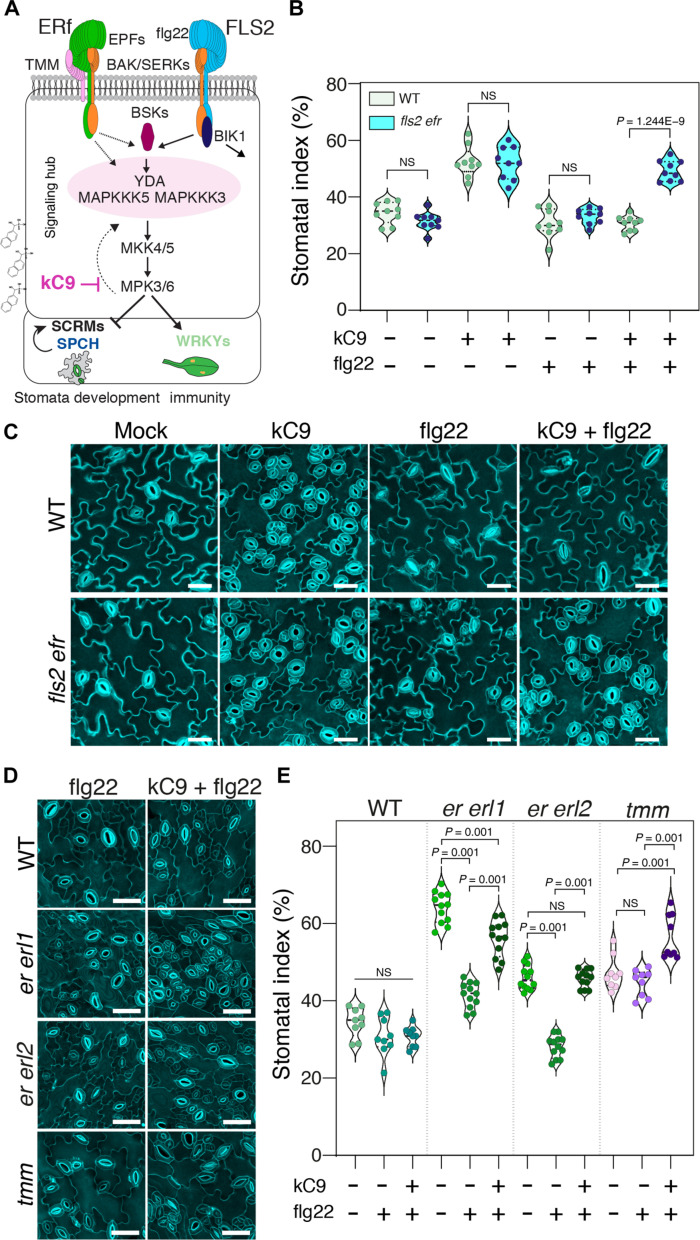
Activation of immune signaling nullifies excessive stomata formation by kC9 or *erecta* family mutations. (**A**) Schematic representation of both the developmental and immune pathways [modified from (*26*)]. Note that both pathways do share signaling components. (**B**) SI of WT (*n* = 9) compared to *fls2 efr* (*n* = 9) double-mutant seedlings treated with either mock (NS: 0.0901), kC9 (NS: 0.8927), flg22 (NS: 0.2065), or kC9 + flg22 (****P = 1.2435 × 10^−9^). Note that flg22 treatment can nullify kC9’s effect in WT but not *fls2 efr* double-mutant plants. Two-tailed unpaired Student’s *t* test was performed. (**C**) Representative confocal images of 7-day-old abaxial cotyledon epidermis from WT (top) and *fls2 efr* seedlings (bottom) corresponding to (B). Scale bars, 40 μm. (**D** and **E**) Activation of the immunity pathway by flg22 reduces the SI of stomatal development receptor mutants, while cotreatment with kC9 can reverse the effect. (D) Representative confocal images of 7-day-old abaxial cotyledon epidermis from WT, *er erl1*, *er erl2* double-mutant, and *tmm* single-mutant seedlings (from top to bottom) treated with either flg22 (left panel) or kC9 + flg22 (right panel). Scale bars, 50 μm. (E) SI of WT (*n* = 9) and stomatal component mutants (*er erl1*, *n* = 12; *er erl2*, *n* = 12; *tmm*, *n* = 9; from left to right) treated with either mock, flg22, or kC9 + flg22. The SI in WT does not show a significant change when treated with either flg22 or kC9 + flg22 compared to mock. In contrast, the SI is significantly reduced (**P < 0.001) in both *er erl1* and *er erl2* double-mutant plants upon flg22 application. kC9 + flg22 coapplication significantly increases (**P < 0.001) the SI for all three receptor mutants. One-way ANOVA followed by Tukey’s post hoc analysis was performed. For exact *P* values, see dataset S1.

Signal transduction pathways regulating stomatal development and immune response are initiated upon the recognition of distinct ligands (EPFs versus flg22) by cognate primary receptors (ERECTA family versus FLS2) (*14*, *31*). Yet, many of their signaling components are shared (*29*) (Fig. 5A). This raises an important, unresolved question of how their signal specificity can be maintained. We identified kC9 as a direct MPK6 inhibitor affecting stomatal development (Figs. 1 to 3). Moreover, kC9 potentially attenuates the flg22-mediated MAPK activation (Fig. 4). This prompted us to explore kC9 as a molecular tool to dissect the signal specificity.

kC9 application did not affect the expression levels of *ERECTA* and *FLS2*, the main receptors for stomatal development and innate-immunity response, respectively (*11*, *60*) (Fig. 4C). To address whether signal transduction pathways that enforce stomatal development are safeguarded from the activation of MPK6 by other (e.g., immunity) pathways, we conducted a separate cotreatment of kC9 and flg22 peptide on WT seedlings. As previously demonstrated (*61*), treatment of flg22 alone does not affect stomatal development (Fig. 5, B and C). Notably, however, the flg22 treatment completely blocked the excessive stomatal development triggered by kC9, resulting in the SI being insignificant from the mock-treated seedlings (Fig. 5, B and C). The effect of flg22 to nullify kC9 is dependent on the presence of the corresponding immune receptor: *fls2 efr* mutant seedlings, which lack the major immune receptors FLS2 and EFR, are capable of fully responding to kC9 to increase stomatal development regardless of the presence or absence of flg22 (Fig. 5, B and C). These results indicate that activation of the flg22-FLS2 immune signaling pathway can fully nullify kC9’s action as an MPK6 inhibitor triggering excessive stomatal development. These findings argue against the mechanism that separates MAPK cascades activated by different ligand-receptor pairs to safeguard signal specificity.

### flg22-mediated signal activation mitigates the loss of ERECTA family receptors

We observed that flg22 application nullifies the effects of kC9 (Fig. 5, B and C). Because kC9 shows reduced binding to CA-MPK6 (Fig. 3), it is plausible that kC9 cannot effectively bind to and inhibit the MPK6 (and likely MPK3) activated by flg22 treatment. If so, how can a stomatal cluster phenotype be “rescued”? It could be possible if the activated FLS2 signaling penetrates into the stomatal development pathway in the presence of kC9. This raises a question of whether this “merger” of signaling pathways occurs because of the artificial influence of chemical compounds or an inherent attribute of the endogenous pathways. To address this question, we examined the effects of flg22-induced immune signal activation on single and double loss-of-function *erecta* family mutants that are partially compromised in MAPK activation (*22*) (Fig. 5, D and E, and fig. S6). As previously reported (*11*), single loss-of-function mutants of *erl1* and *erl2* do not exhibit any stomatal phenotypes. However, we found that these single mutations attenuate the efficacy of the flg22 peptide in nullifying the elevated SI by kC9 (fig. S6). This trend becomes more exaggerated in *er* single as well as *er erl1* and *er erl2* double mutants, where flg22 application no longer fully counteracts kC9 (Fig. 5, D and E, and fig. S6).

Under normal (mock) conditions, these higher-order mutant seedlings exhibit higher SI than WT owing to reduced receptor populations (fig. S6). Unexpectedly, even in the absence of kC9, the application of the flg22 peptide alone is sufficient to inhibit the elevated SI in *er*, *er erl1*, and *er erl2* mutant seedlings (Fig. 5E and fig. S6). These effects were less pronounced in the *tmm* mutant, perhaps owing to its auxiliary role in the functional homeostasis of EPF-ERECTA family signaling. Together, we conclude that under the reduced population of upstream ERECTA family receptors, the activation of the MAPK cascade by the flg22-mediated immunity pathway is sufficient to compensate for the reduced MAPK activation in the EPF-ERECTA family stomatal development signaling pathways. The findings suggest that maintaining the specificity of signaling pathways relies on a proper homeostasis of available MAPKs that are yet to be activated.

### Vulnerability of stomatal lineage cells to the immune signaling

Our study revealed that kC9’s effects in elevating stomatal numbers by inhibiting MPK6 activity can be counteracted by the activation of the MAPK cascade by the flg22-FLS2 immune signaling pathway (Fig. 5 and fig. S6). Moreover, even in the absence of kC9, flg22 can mitigate the excessive stomatal development because of reduced populations of the upstream ERECTA family receptors (Fig. 5 and fig. S6). These findings challenge the prevailing notion of signal specificity (*43*). However, it is important to note that our experimental setups thus far entail a constant treatment of kC9 and/or flg22. Therefore, they do not depict which specific developmental stages of the seedling epidermis are affected by the flg22-FLS2–mediated signal cross-regulation.

**Fig. 6.**
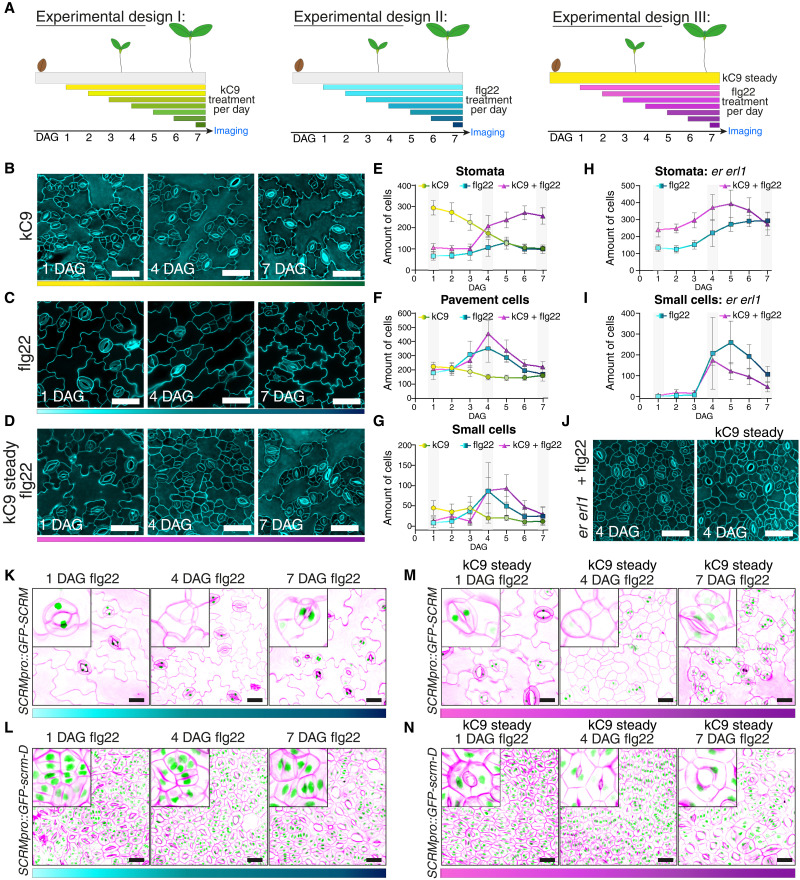
Sequential flg22 application reveals the developmental stage labile to the immune signal activation. (**A**) Schematics. kC9 treatment (left: experimental design I, yellow to green), flg22 treatment (middle: experimental design II, light blue to dark blue), and flg22 treatment in the steady level of kC9 (right: experimental design III, pink to purple). (**B** to **G**) Chemical and/or peptide treatment over time depicted in (A). [(B) to (D)] Representative confocal images of 7-day-old WT abaxial cotyledon epidermis from seedlings treated on 1, 4, or 7 DAG with 50 μM kC9 (B), 0.1 μM flg22 (C), or 0.1 μM flg22 where kC9 steadily was present for 7 days (D). Note an accumulation of small epidermal cells in (F) on 4 DAG. Scale bars, 50 μm. [(E) to (G)] Quantitative analysis of different epidermal cell types of WT cotyledon abaxial seedlings treated over time. For exact values, see dataset S1. The number of stomata (E), epidermal pavement cells (F), and small cells representing meristemoids and SLGCs (G) per unit area (0.339178112 mm^2^) are plotted. Error bars, SD. (**H** and **I**) Quantitative analysis of different epidermal cell types in *er erl1* cotyledon abaxial seedlings treated over time on experimental designs II (light blue to dark blue) and III (pink to purple). The number of stomata (H) and small cells representing meristemoids and SLGCs (I) per unit area (0.339178112 mm^2^) are plotted. Error bars, SD. (**J**) Representative confocal images of 7-day-old *er erl1* cotyledon abaxial seedling epidermis treated on 4 DAG with 0.1 μM flg22 in the presence or absence of kC9. Scale bars, 50 μm. (**K** to **N**) Representative confocal images of 7-day-old cotyledon expressing *SCRMpro::GFP*-*SCRM* [(K) and (M)] or *SCRMpro::GFP*-*scrm*-*D* [(L) and (N)] treated on 1, 4, or 7 DAG with 0.1 μM flg22 in the absence [(K) and (L)] or presence [(M) and (N)] of kC9. Scale bars, 25 μm.

To delineate a developmental window where the altered MAPK activity by kC9 and/or flg22 affects stomatal differentiation, we designed a series of time-course experiments. Normally, under mock condition, germinating seedlings exhibit active cell proliferation around 4 days after germination (DAG) and initial epidermal patterns mature around 7 DAG (fig. S7). In our three experimental designs, either kC9 (experimental design I) or flg22 (experimental design II) was applied consecutively on each day after germination up to day 7. In the cotreatment experiment (experimental design III), seedlings were germinated in the constant presence of kC9, with the flg22 peptide sequentially applied on each day after germination (Fig. 6A). For all of these experiments, cotyledon abaxial epidermis was observed at day 7 (Fig. 6, B to D; and fig. S8, A to C). In experimental design I, the number of stomata declined as the duration of kC9 treatment shortened (Fig. 6, B and E, and fig. S8A). The number of epidermal pavement cells stayed constant, whereas that of small stomatal lineage cells slowly decreased as stomata differentiated (Fig. 6, F and G, and fig. S8A). Thus, the number of stomata inversely correlated with the duration and early application of kC9 treatment.

In contrast, time window–specific effects of flg22 on stomatal development were observed when we performed experimental design II, a sequential application of the flg22 peptide (Fig. 6, C and E to G, and fig. S8B). Whereas the number of stomata remained relatively constant throughout the time course, small stomatal lineage–like cells overwhelmingly increased, peaking at 4 DAG (4 days of flg22 treatment) (Fig. 6, C, E, and G, and fig. S8B). The vast increase in the small stomatal lineage–like cells (and, consequently, pavement cells) is more notable in experimental design III, where flg22 was sequentially applied under the constant presence of kC9, again peaking in numbers at 4 to 5 DAG (4 to 3 days of flg22 treatment) (Fig. 6, D, F, and G, and fig. S8C). In the *er erl1* double-mutant background wherein ERECTA family receptor signaling is suboptimal, the effects of flg22 to vastly increase the small cells at 4 to 5 DAG became even more exaggerated (Fig. 6, H to J). Under a normal condition without any kC9 and/or flg22 application, 4 DAG is the time when stomatal lineage cells are actively proliferating, with *er erl1* double mutation intensifying the cell proliferation (fig. S7).

To further explore the identity of these small cells induced by the flg22 application, we examined the expression of SCRM protein, a SPCH/MUTE/FAMA partner that is directly targeted by MPK6 (*24*, *25*). For this purpose, seedlings expressing a fusion protein of GFP and SCRM (*SCRMpro::GFP*-*SCRM*) and its stabilized version (*SCRMpro::GFP*-*scrm*-*D*) each driven by the endogenous promoter were subjected to experimental designs II and III (Fig. 6, A and K to N). SCRM is known to accumulate in the nucleus of all stomatal lineage cells, including stomatal lineage ground cells (SLGCs) (*25*). We found that most of the massively accumulated small cells at 4 DAG do not exhibit GFP signals (Fig. 6, K and M, middle panels). Likewise, transcriptional reporters of stomatal precursor cells, including *SCRMpro::nucGFP*, *MUTEpro::nucYFP*, and *TMMpro::GUS*-*GFP*, are barely detectable when flg22 was applied at 4 DAG (fig. S8, D to I), indicating that these small cells lost the proper stomatal precursor identity. In contrast, flg22 has no effects on GFP-scrm-D accumulation nor the “stomata-only” phenotype conferred by GFP-scrm-D (Fig. 6, L and N). Collectively, these results suggest that flg22 treatment at 4 DAG, when stomatal lineage cells are actively proliferating (*62*), triggers the arrest and subsequent withdrawal of stomatal lineage fate through the MPK6-mediated degradation of SCRM. Moreover, the remarkably narrow developmental window of the flg22 action implies that stomatal lineage cells are most sensitive to the activation of the MAPK cascade by otherwise unrelated immune signaling pathways when the cells are in the decision-making process. This vulnerability likely stems from the intricate cellular response to a proper activation status of MAPKs.

## DISCUSSION

Through a small compound library screening, we identified kC9 as a potent inducer of stomatal development by inhibiting the canonical EPF-ERECTA family signaling pathway. Further comprehensive chemical genetics analyses together with in vitro biochemical, biophysical, and structural studies revealed that kC9 is an inhibitor of plant MAPKs. We found that the activation of the immune RK signaling pathway fully nullifies the excessive stomatal differentiation by kC9. This cross-regulation is not an artifact of chemical application but rather the result of signal integration when the activable upstream receptors for stomatal development are limited. Last, we revealed the cell-state specificity upon which the immune signaling pathway intervenes with stomatal differentiation through the activation of a shared MAPK cascade.

Our docking modeling of kC9 with the structurally solved MPK6 and subsequent binding and kinase activity assays, backed up by the structure-activity relationship analysis of the kC9 analogs, revealed that kC9 weakly binds to the ATP-binding pocket of MPK6 and inhibits its activity (Fig. 3 and figs. S1 and S4). The ATP-binding pocket within the catalytic cleft is shared by protein kinases (*63*), as such kC9 may not likely be a highly selective kinase inhibitor. Aligning with this view, kC9 (HNMPA) is a known insulin receptor tyrosine kinase inhibitor (*44*). As we have not profiled the effects of kC9 on the entire Arabidopsis kinome, our study does not exclude the possibility that kC9 may bind to and inhibit MAPKs other than MPK3 and MPK6 as well as kinases in plants beyond MPKs. Nevertheless, as demonstrated by the extensive phenotypic analyses and the in vivo MPK3/6 activity assays (Figs. 1 and 2 and fig. S2), it is clear that kC9 inhibits MAPK activation downstream of the EPF-ERECTA family. We did not detect the binding of kC9 to MKK5 (fig. S4 and table S1), one of the upstream MAPKKs of MPK3/6 in stomatal development (*21*). This further delineates a kC9 target to MPK6 (and MPK3) within the MAPK cascade. One explanation for the strong propensity of kC9 to affect stomatal development is that the binding of kC9 to MPK6 perturbs the recruitment of its substrate, SCRM (Fig. 3, E to G). SCRM has a unique bipartite anchoring module (KiDoK, canonical kinase docking site and SCRM-specific KRAAM motif) to associate with MPK6, and it is required for the posttranslational regulation of SPCH and, hence, proper stomatal patterning (*24*). MPK6 phosphorylates diverse regulators of development and environmental responses, including MYC2 in jasmonic acid response and WRKYs in immunity and pollen development (*55*, *64*, *65*). Although the structural basis of MPK6 binding mode with these substrates is not known, further studies may unravel exactly how kC9 affects additional MPK6-mediated pathways, as suggested by our transcriptome analysis (Fig. 4).

The diminished binding affinity of kC9 to the preactivated MPK6 (Fig. 3B) enabled us to investigate whether the preactivation of the upstream signaling receptors can forestall kC9 from inhibiting the MPK6-mediated inhibition of stomatal development. Notably, kC9’s action can be nullified not only by the bona fide ligands for ERECTA family RKs, EPF1 and EPF2, but also by the immunity ligand flg22 for FLS2 (Figs. 2 and 5). In the cell signaling field, it is widely established that extracellular signals perceived by the cell-surface receptors are relayed to a specific MAPK cascade via selective activation of scaffold proteins (*66*). The well-known example is yeast mating and osmolarity response, both of which are mediated by the shared MAPKKK Ste11. There, the activated upstream signaling recruits distinct scaffold proteins, Ste5 and Pbs2 for mating and osmosis, respectively, to ensure signal specificity (*67*–*69*). In plants, RACK1 (RECEPTOR FOR ACTIVATED KINASE C) functions as a scaffold protein that bridges heterotrimeric G proteins to an MAPK cascade (*70*). Yet, scaffold proteins that separate LRR-RK immunity versus development signaling remain elusive (*43*).

On the basis of our findings, we propose that the signal specificity of the EPF-ERECTA family pathway can be ensured by its own fully activable state. During epidermal development, ERECTA family RKs are constantly detecting multiple EPF/EPFL peptides (*26*, *46*), which likely elicits optimal, continual activation of the downstream MAPK cascade. This signaling dynamics itself prevents the inappropriate signal bleed-through from other inputs. Our findings accord with a previous report that the constitutive activation of the FLS2 immune signaling failed to affect stomatal development when ERECTA family signaling is fully active (*61*). Moreover, our proposed mechanism reconciles the previously reported inconsistencies regarding the relationship between YODA and MAPKKK3/MAPKKK5 in development and immunity, whether they act together or antagonistically (*38*, *39*, *55*). Sun *et al.* (*37*) observed that the flg22 peptide triggers excessive phosphorylation of MPK3/6 in *erecta* family single- and double-mutant backgrounds and concluded that the immune signaling can be hyperactivated when the population of ERECTA family receptors is limited. However, this could be the sign of immunity-to-development signal cross-regulation as observed in our study (see Fig. 5, D and E; and fig. S6, A and B). On the other hand, whether the EPF-ERECTA pathway can also cross-activate the immune signal remains unclear. It has been reported that ERECTA regulates plant disease resistance via the YODA MAPK cascade (*71*–*73*). Since ERECTA’s role in immunity does not seem to require EPF peptides (*71*), it may involve different mechanisms, such as an indirect resistance through modulation of downstream gene expression that influences cell wall properties (*71*, *73*).

It is important to note that even under a suboptimal ERECTA family signaling pathway, the activated flg22-FLS2 signaling pathway can only reduce the numbers of stomata to the WT level (see Fig. 5, D and E, and fig. S6, A and B). In contrast, induced EPF1 or EPF2 overexpression severely diminishes the number of stomata even in the presence of kC9 (Fig. 2, D and E). These observations imply that the influence of the immune signal on the MAPK cascade in stomatal development is safeguarded by a functional MAPK homeostasis, which is regulated by the strengths of the signal input (i.e., ERECTA family RKs). Consequently, the cross-regulation of the MAPK cascade by immunity signal inputs cannot exceed the basal (normal) level. While the exact molecular nature of the threshold that limits cross-signal activation is unknown, it is tempting to hypothesize that the MAPK cascade components form a shared dynamic signaling hub (Fig. 5A). This hub can sense and associate with a partially compromised upstream receptor to allow signal activation beyond the pathway boundaries, reflecting how signaling input strength and MAPK homeostasis contribute to signal specificity.

Our time-course experiments (Fig. 6) revealed the narrow developmental window of the flg22-induced inhibition of stomatal development, highlighting the vulnerability of proliferating stomatal lineage cells to the immune signaling. These early stomatal lineage cells undergo asymmetric cell divisions, where the differential MAPK activities will be translated into the cell-fate decision to become a meristemoid or an SLGC (*74*). Consistently, MAP KINASE PHOSPHATASE 1 (MPK1), which attenuates MAPK activity, influences stomatal differentiation during the early proliferating precursor state (*75*). In addition to the EPF-ERECTA family–mediated MAPK signaling, dynamically assembled polarity proteins including BASL (BREAKING OF ASYMMETRY IN STOMATAL LINEAGES) differentiate the MAPK activity between the two daughter cells via positive feedbacks (*76*, *77*). As such, it is conceivable that an additional spike of immune signal activation disturbs the intricacy of differential MAPK activity required for maintaining the meristemoid identity.

Last, exploring the biological significance of the observed cross-regulation of stomatal development by flg22-FLS2 is the most exciting yet challenging future direction. It is tempting to speculate that stomatal immunity extends beyond the regulation of stomatal movement to that of stomatal development. As an immediate solution, rapid stomatal closure prevents pathogen entry (*29*). Perhaps, as a long-term solution, decreasing the number of stomata may limit the “entry point” for forthcoming pathogens. While this could be a fascinating hypothesis, it is important to note that our study was conducted in a purely experimental setting. It is unlikely that seedlings experience a sudden exposure to pure flg22 peptides in a natural environment. Perhaps, our finding may reflect the idea that the immediate plant response toward sudden exposures to pathogen-associated molecular signatures is to activate a general stress response (*78*). Such pathways are mediated by the shared MAPK cascade, many involving MPK3/6 (*79*). Our work thus may have unraveled a hidden developmental response to reduce the number of stomata when exposed to acute biotic/abiotic stresses. Understanding how different cell types and cell states interpret and respond to development and developmental signals is a fundamentally important future direction to gain insight into the dynamics and heterogeneity in cellular signal specificity and integration.

## MATERIALS AND METHODS

### Plant materials

The Arabidopsis Columbia (Col) accession was used as WT. The following mutants and reporter transgenic lines were reported previously: *er*-*105*, *erl1*-*2*, *erl2*-*1*, *er*-*105 erl1*-*2*, *er*-*105 erl2*-*1*, *erl1*-*2 erl2*-*1*, and *er*-*105 erl1*-*2 erl2*-*1* (*80*); *spch*-*3*, *fama*, and *E994* (*81*); *mute*-*2* (*82*); *MUTEpro::nucYFP* (*83*); *SCRMpro::GFP*-*SCRM* and *SCRMpro::GFP*-*scrm*-*D* (*25*); *TMMpro::GUS*-*GFP* (*18*); *tmm*-*KO* (*12*), *epf1*-*1*, *epf2*-*1*, *Est::EPF1*, and *Est::EPF2* (*14*); *mpk3*, *mpk6*, and *NtMEK2_DD_* (*21*); *fls2 efr* (*84*); *bak1*-*5*, *serk1*-*1*, and *serk2*-*1* (*15*); and *CA*-*YDA* (*85*), *amiYDA#1*, and *amiYDA#2* (*37*). Seedlings and plants were grown under a long-day condition (for general growth and analysis) or a continuous light condition (for RNA-seq analysis to minimize circadian effects) at 21°C.

### Chemical screening

For the initial screen, we used the Arabidopsis E994 line that expresses stomatal guard cell–specific endoplasmic reticulum–localized GFP. Three E994 seeds were germinated in each well of a 96-well microtiter plate (TL5003; True Line) supplemented with 95 μl of 12 MS media. At 1 DAG, 5 μl of individual chemical compounds [dimethyl sulfoxide (DMSO)/medium mixture with the ratio of 1:9 prepared from 10 mM master stock in 100% DMSO] from the Institute of Transformative Bio-Molecules (ITbM) chemical compound library (*48*) was added to each well at the final concentration of 50 μM. The seedlings were grown for 9 days at 140 rpm and 22°C in continuous light, and subsequently, GFP signals were examined under a Zeiss SteREO Discovery V20 fluorescent microscope. The compounds that increased GFP-positive stomata were subjected to a secondary screen to validate the reproducibility of the compounds to increase the number of stomata. Those chemicals that passed the secondary screen were moved to a tertiary screen using seedlings expressing *TMMpro::GUS*-*GFP* (*18*), which marks stomatal lineage cells. Confocal images of *TMMpro::GUS*-*GFP* abaxial cotyledon epidermis are subsequently analyzed using our pipeline: The original images were converted to black/white, and the area mean intensity of black pixels, which correspond to GFP signals from three biological replicates, was calculated and plotted. Those compounds that increased the area mean intensity signals of stomatal lineage cells >1.5-fold (*P* < 0.05) are classified as stomatal number–increasing compounds.

### Chemical synthesis and NMR analysis

We synthesized kC9 (HNMPA) (**2**) and its derivatives from commercially available 2-naphthaldehyde, 1-pyrenecarboxaldehyde, and 2-bromomethyl naphthalene. Phosphonic acid diesters (**1**, **5**, **7**, and **9**) were prepared by nucleophilic addition and substitution reactions with dialkyl phosphites. Acetylation of α-hydroxy group of **1** led to **3**. Lewis acid treatment of phosphonic acid diesters (**1**, **3**, **5**, **7**, and **9**) successfully provided phosphonic acids (**2**, **4**, **6**, and **8**) and phosphonic acid monoester (**10**), respectively. ^1^H, ^13^C{^1^H}, and ^31^P{^1^H} NMR was recorded on a JEOL JNM-ECA500 (500 MHz for ^1^H, 125 MHz for ^13^C, and 202 MHz for ^31^P) spectrometer. Chemical shifts were reported in parts per million (d), and coupling constants were reported in hertz. High-resolution mass analyses were submitted to the Mass Spectrometry Laboratory (Molecular Structure Characterization Unit) at RIKEN. Thin-layer chromatography was performed on Merck 60 F254-precoated silica gel plates. For detailed chemical synthesis procedures as well as NMR spectral data, see document S1.

### Chemical treatment

Compound CL1 (SC-58125, catalog no. 70655) was purchased from Cayman Chemical (Ann Arbor, MI). Bubblin (MS-6628) was purchased from Key-Organics (Cornwell, UK). kC9 and its analogs were synthesized (see the above section). kC9 and its phosphonic acid ester form {*p*-[[(acetyloxy)methoxy]-2-naphthalenylmethyl]-bis[(acetyloxy)methyl] ester phosphonic acid, **1**} are also commercially available (Abcam, ab141566 and ab141567, respectively). These compounds were diluted in DMSO and subjected to chemical treatment. For experiments in Figs. 1 (B and F) and 5E, seedlings were incubated for 8 days and observed at day 9. For all other experiments, seedlings were incubated for 7 days and observed at day 7. For a dose-dependent analysis, kC9 was diluted to respective concentrations from a 100 mM stock solution and subjected to aforementioned treatments. For phenotypic analyses, kC9 and its derivatives were applied to Arabidopsis mutants and reporter lines using 24-well microtiter plates (Life Science, Durham, US, ref.: 351147). For estradiol induction of EPF1 (*Est::EPF1*) and EPF2 (*Est::iEPF2*) overexpression, germinated seedlings were grown in the presence of 5 μM estradiol with or without 50 μM kC9. The flg22 peptide was either synthesized at the ITbM Peptide Protein Center or obtained from BioSynthesis (lot no. K1254-1). Seedlings were treated with 50 to 100 nM flg22 peptide in 12 MS liquid media in the presence or absence of 50 μM kC9.

### Confocal microscopy

For confocal microscopy, cell peripheries of seedlings were visualized with propidium iodide (PI) (Sigma-Aldrich, P4170). Images were acquired using LSM800 (Zeiss) using a 20× lens. The GFP, YFP reporter, and propidium iodide signals were detected at 488 nm and the 410 to 546 nm emission range and with excitation at 561 nm and the 582 to 617 nm emission range. Raw data were collected with 1024-pixel by 1024-pixel images. Alternatively, images were acquired using Stellaris-8 FALCON (Leica - Mannheim, Germany) using either a 40× or 63× oil lens for high-resolution imaging and a 20× water lens for data acquisition. Signals were detected for the following conditions: GFP, excitation at 488 nm and emission from 490 to 546 nm; YFP, excitation at 514 nm and emission from 520 to 560 nm; propidium iodide, excitation at 561 nm and emission from 570 to 620 nm. Signals were visualized sequentially using separate HyD detectors (HyDX/HyDS). For qualitative image presentation, Fiji (ImageJ) as well as Adobe Photoshop CS6 was used to trim and uniformly adjust the contrast/brightness.

### Plasmid construction

For detailed information on the plasmids constructed in this study, see table S4. See table S5 for a list of primer oligo DNA sequences. For pDONR221_P3P2, coding sequences of SCRM and SPCH (*24*) were amplified (Phusion, NEB) and cloned via a Gateway BP reaction (Thermo Fisher Scientific) into *pDONR221_P3P2*. Subsequently, a Gateway LR reaction (Thermo Fisher Scientific) was performed using *pBiFCt:2in1*-*CC* (256) as the destination vector. For *pDONR221_P1P4*, the MPK6 coding sequence (*24*) was cloned via a Gateway BP reaction (Thermo Fisher Scientific, lot no. 2536741) into *pDONR221_P1P4*. Subsequently, a Gateway LR reaction (Thermo Fisher Scientific) was performed using *pBiFCt:2in1*-*CC* (256) as the destination vector. To create dominant-negative (DN, K92M_K93R) and constitutive-active versions (CA, D218G_E22A) of MPK6 for protein purification, site-directed mutagenesis was performed using the Prime STAR (Takara Bio, lot no. N5101FA) protocol and *pGEX4T*-*1_MPK6*Δ*Nt (29*–*305)* (*24*) as the backbone.

### Ratiometric biomolecular fluorescence complementation (rBiFC)

The Gateway-compatible 2in1 system (*86*, *87*) was used to test the interaction between SCRM and MPK6 in the presence or absence of kC9. SPCH together with MPK6 was used as the negative control. Amplicons with recombination sites were cloned either in *pDONR221*-*P3P2* or *pDONR221*-*P1P4* by BP clonase. A subsequent LR clonase reaction was performed with the pBiFCt-2in1-CC destination vector. While both SPCH and SCRM were fused to N-terminal half YFP (nYFP), MPK6 was fused to C-terminal half YFP (cYFP) on the same plasmid, each expressed under the control of a 35S promoter. An internal expression control (35S::RFP) is also included on the plasmid. Interaction strength was determined by the YFP/RFP ratio. For this, 4-week-old leaves of *N. benthamiana* were transfected with *Agrobacterium tumefaciens*, carrying respective destination vectors at an optical density of 0.2 in the presence or absence of 100 μM kC9 and incubated for 2 days in the plant room under short-day conditions. Confocal microscopy single-plane images of nuclei were taken by using the Stellaris-8 FALCON (Leica, Mannheim, Germany) and a 63× oil lens. For comparison of interaction strength rBiFC, identical confocal settings were used for respective experiments (*n* = 3, ~50 nuclei each). The average fluorescence signal intensity for YFP and RFP was measured by using ImageJ version 1.8.0_66. Calculation of mean YFP/RFP ratios was performed in Excel, and the graph was assembled in GraphPad Prism version 10.

### RNA sequencing and analysis

Three-day-old Arabidopsis WT seedlings were treated with mock (5% DMSO) or 50 μM kC9 (in 5% DMSO) for 6 and 24 hours, and total RNAs were extracted and purified using the RNeasy Plant Mini Kit (QIAGEN). Each RNA sample (three independent RNA samples for each treatment) was prepared from a pool of 30 whole seedlings. Three micrograms of purified RNA was used for RNA-seq as described previously (*88*). Briefly, after the RNA integrity was confirmed by running the RNA samples on an Agilent RNA 6000 Nano Chip (Agilent Technologies), RNA-seq libraries were prepared using the Illumina TruSeq Stranded mRNA LT Sample kit. The resulting barcoded libraries were pooled and sequenced on an Illumina NextSeq500 sequencing platform, and 75–base pair single-end reads were obtained. Sequences of RNA-seq experiments were mapped on TAIR 10 genome using TopHat2 (version 2.1.0, https://github.com/infphilo/tophat) with default options, and the Illumina reads were mapped and the read counts were calculated. Transcript expression and DEGs were determined using the EdgeR GLM approach (*89*), with genes having FDRs <0.01 classified as DEGs. Scaled expression values (fold change) were used for SOM-based clustering (*90*). To assess the enrichment of GO terms within the various sets of DEGs, GO enrichment analysis was conducted using Cytoscape with the BiNGO add-on (http://apps.cytoscape.org/apps/bingo) (*91*). The GO term finder evaluates the overrepresentation of GO categories using a hypergeometric test, with FDR correction for multiple testing (*P* ≤ 0.05). See dataset S2 for specific gene sets. All RNA-seq data have been deposited to DDBJ (DNA Data Bank of Japan) with accession number PRJDB18465.

### Recombinant protein expression and purification

MPK6ΔNt (29 to 305) (*24*), MPK6_D218G_E222A_ (CA-MPK6), MPK6_K92M_K93R_ (DN-MPK6), MKK5, MKK5_DD_, and SCRM (1 to 494) were cloned into the pGEX-4T-1 vector with an N-terminal glutathione *S*-transferase (GST) tag and a thrombin cleavage sequence. For protein expression, the constructs were transformed into *Escherichia coli* strain BL21-CodonPlus. For each transformant, a single colony was selected and incubated in 10 ml of LB liquid medium. The overnight-incubated *E. coli* suspensions were transferred to 1 liter of LB medium and incubated at 37°C for around 2 hours until the optical density at 600 nm reached 0.4 to 0.6. Isopropyl β-d-1-thiogalactopyranoside was added to the cultures, and the strains were incubated for further 6 hours. GST-fused proteins were purified using glutathione agarose resin. The soluble portion of the cell lysate was loaded onto a GST-Sepharose column. Nonspecifically bound proteins were removed by washing the column with 20 mM tris (pH 8.0) and 200 mM NaCl. The bound GST-fused protein was eluted with 10 mM glutathione, 20 mM tris (pH 8.0), and 200 mM NaCl (pH 8.0). The GST-fused proteins were exchanged with phosphate-buffered saline (PBS) buffer, and then the solution was treated with 50 μg of thrombin for 10 to 12 hours at 16°C. The GST portion of the protein was cleaved during thrombin digestion, and then the whole solution was reloaded onto the GST column to obtain pure protein. The purified proteins were further purified by gel filtration on a Superdex-200 (GE) column using fast protein liquid chromatography and phosphate buffer (pH 7.2) as the eluent. The purity of the protein was checked by SDS–polyacrylamide gel electrophoresis.

### Isothermal titration calorimetry (ITC)

Binding of the small molecules kC9 and kC9-3 to MPK6ΔNt (29 to 305), MPK6_D218G_E222A_ (CA-MPK6), MPK6_K92M_K93R_ (DN-MPK6), MKK5, kC9-3 to MPK6ΔNt-ATP (29 to 305), and SCRM KiDoK peptide to MPK6ΔNt (29 to 305) in the presence and absence of kC9 was characterized at 25°C using a Malvern PEAQ-ITC microcalorimeter. All protein samples were dialyzed overnight using PBS buffer containing 2% DMSO. Small molecules kC9 and kC9-3 were dissolved in PBS buffer containing 2% DMSO. Titrations were performed by injecting 1 × 0.5–μl and 17 × 2–μl aliquots of 1 mM kC9/kC9-3 to 30 mM protein in PBS buffer (pH 7.4), containing 2% DMSO. All titrations were carried out at least twice. The raw data were corrected using buffer and protein controls and analyzed using the software supplied by the manufacturer.

### Biolayer interferometry (BLI)

The binding affinities of kC9 and kC9-3 to MPK6 and MPK6_D218G_E222A_ (CA-MPK6), MPK6_K92M_K93R_ (DN-MPK6), and SCRM KiDoK peptide to MPK6 in the presence and absence of kC9 were measured using the Octet Red96 system (ForteBio, Pall Life Sciences) following the manufacturer’s protocols. The SCRM KiDoK and scrm-D KiDoK peptides were custom synthesized and biotinylated (BioSynthesis). The optical probes coated with anti-GST or streptavidin were first loaded with 500 nM GST-tagged proteins or biotinylated SCRM KiDoK peptide before kinetic binding analyses. The experiment was performed in 96-well plates maintained at 25°C. Each well was loaded with a 200 μl reaction volume for the experiment. The binding buffer used in these experiments contained 1× PBS supplemented with 2% DMSO, 0.02 Tween 20, and 0.1% bovine serum albumin. The concentrations of kC9/kC9-3 as the analyte in the binding buffer were 100, 50, 25, 12.5, 6.25, 3.12, and 1.56 μM, and the concentrations of MPK6/MPK6:kC9 (1:1.5) as the analyte in the binding buffer were 150, 75, 37.5, 18.75, 9.37, and 4.68 nM. There was no binding of the analytes to the unloaded probes as shown by the control wells. Binding kinetics to all seven concentrations of the analytes were measured simultaneously using default parameters on the instrument. The data were analyzed using Octet data analysis software. The association and dissociation curves were fit with the 1:1 homogeneous ligand model. The *k*_obs_ (observed rate constant) values were used to calculate *K*_d_ with steady-state analysis of the direct binding.

### Docking modeling

Molecular docking of ATP and kC9 (**2**) and its analogs (kC9-3 and kC9-8) to MPK6 [PDB (Protein Data Bank) ID: 6DTL] (*24*) was carried out using the HADDOCK approach (*51*, *52*). Ambiguous interaction restraints were selected on the basis of the preliminary AUTODOCK model (*92*) and ATP-interacting residues with ERK2 kinase (*93*). After initial rigid body docking, the top 1000 structures that had the best intermolecular energies were then sequentially subjected to semiflexible simulated annealing and explicit solvent refinement. The pairwise “ligand interface root mean square deviation (RMSD) matrix” over all structures was calculated, and the final structures were clustered using an RMSD cutoff value of 3.5 Å for both ligand and protein. The clusters were then prioritized using RMSD and the “HADDOCK score” (weighted sum of a combination of energy terms). The structures with the best HADDOCK score were used for representation.

### Kinase-Glo luminescence kinase assay

A luminescence assay was used to monitor the phosphotransferase activity of MPK6 kinase in the presence and absence of kC9 and the kC9-3 analog. When activated, MPK6 phosphorylated a peptide substrate by converting ATP to adenosine 5′-diphosphate (ADP). The kinase reaction was stopped, and unreacted ATP was removed using the ADP-Glo reagent (Promega). The kinase detection reagent was added to convert ADP back to ATP, which was then converted to a luminescent signal by the luciferase reaction (Promega, catalog no. V6072). First, the MPK6 protein alone or together with MKK5_DD_ was incubated in 30 μl of reaction buffer: 25 mM tris-HCl (pH 7.5), 12 mM MgCl_2_, 1 mM dithiothreitol, and 50 μM ATP. The mixtures were kept at room temperature for 30 min. In the second step, a 5 μl solution from each of the first reactions was incubated with SCRM/scrm-D in 25 μl of reaction buffer: 25 mM tris-HCl (pH 7.5), 12 mM MgCl_2_, 1 mM dithiothreitol, and 50 μM ATP. The kinase reaction was conducted at room temperature (25 to 27°C) for 30 min. DMSO was used as a negative control for small-molecule inhibitors, and a system without ATP was used to measure background signals. Luminescence was measured using a GloMax 96 Microplate Luminometer (Promega). The data were analyzed using GraphPad Prism 10 software.

### In vivo MAPK phosphorylation activity assays

MAPK phosphorylation activity assays were conducted with slight modifications to the procedures described by Lee *et al.* (*19*). Five-day-old Arabidopsis Col-0 seedlings, initially grown on 12 MS medium plates, were transferred to 12 MS liquid media in 12-well cell culture plates and incubated overnight in a long-day growth room. The kC9 priming treatment was performed by adding kC9 stock to the liquid medium to achieve a final concentration of 50 μM at 6 hours. For flg22 treatment, 10 nM flg22 was applied to medic primed with 50 μM kC9. Seedlings were briefly dried on Kimwipe paper and immediately frozen in liquid nitrogen for 1 hour after peptide treatment. The samples were ground in liquid nitrogen and homogenized in protein extraction buffer [100 mM tris, pH 7.5, 150 mM NaCl, 10% glycerol, 20 mM sodium fluoride, 1.5 mM sodium orthovanadate, 2 mM sodium molybdate, 1 mM phenylmethylsulfonyl fluoride, 1% Triton X-100, 1× protease inhibitor cocktail, 1× phosphatase inhibitor cocktail 2, 1× phosphatase inhibitor cocktail 3, and 10 μM MG-132 (Sigma-Aldrich)]. The protein concentration was determined by using the Bradford assay (Bio-Rad) with standard bovine serum albumin. Equally normalized protein samples were subjected to immunoblot analysis using Anti-Phospho-p44/42 MAPK (Erk1/2) (Thr^202^/Tyr^204^) (catalog no. 9101S, Cell Signaling Technology) as the primary antibody and peroxidase-conjugated goat Anti-rabbit IgG (catalog no. ab205718, Abcam) as the secondary antibody. The same immunoblot membrane was analyzed using an Anti-Actin antibody (catalog no. ab230169, Abcam) as the internal loading control. The immunoblots and exposures are separately adjusted for the blots presented in Figs. 2H and 4D to reliably detect relative signal differences without saturating the signals. The signal intensity of the immunoblot was quantified using Fiji ImageJ.

## Supplementary Materials

### The PDF file includes:

Figs. S1 to S8 Tables S1 to S5 Section S1 Legends for datasets S1 and S2

### Other Supplementary Material for this manuscript includes the following:

Datasets S1 and S2
