# Supplementary material for: Chemical genetics reveals cross-regulation of plant developmental signaling by the immune peptide-receptor pathway
Source: Sci Adv. 2025 Feb 5;11(6):eads3718. doi: 10.1126/sciadv.ads3718 (PMC11797554; doi:10.1126/sciadv.ads3718)
Supplement: Supplementary file 1 — Figs. S1 to S8 Tables S1 to S5 Section S1 Legends for datasets S1 and S2 [file sciadv.ads3718_sm.pdf]

Science Advances
AAAS

Supplementary Materials for

Chemical genetics reveals cross-regulation of plant developmental signaling by the immune peptide-receptor pathway

Arvid Herrmann *et al.*

Corresponding author: Keiko U. Torii, ktorii@utexas.edu

Sci. Adv. **11**, eads3718 (2025)
DOI: 10.1126/sciadv.ads3718

The PDF file includes:

Figs. S1 to S8
Tables S1 to S5
Section S1
Legends for datasets S1 and S2

Other Supplementary Material for this manuscript includes the following:

Datasets S1 and S2

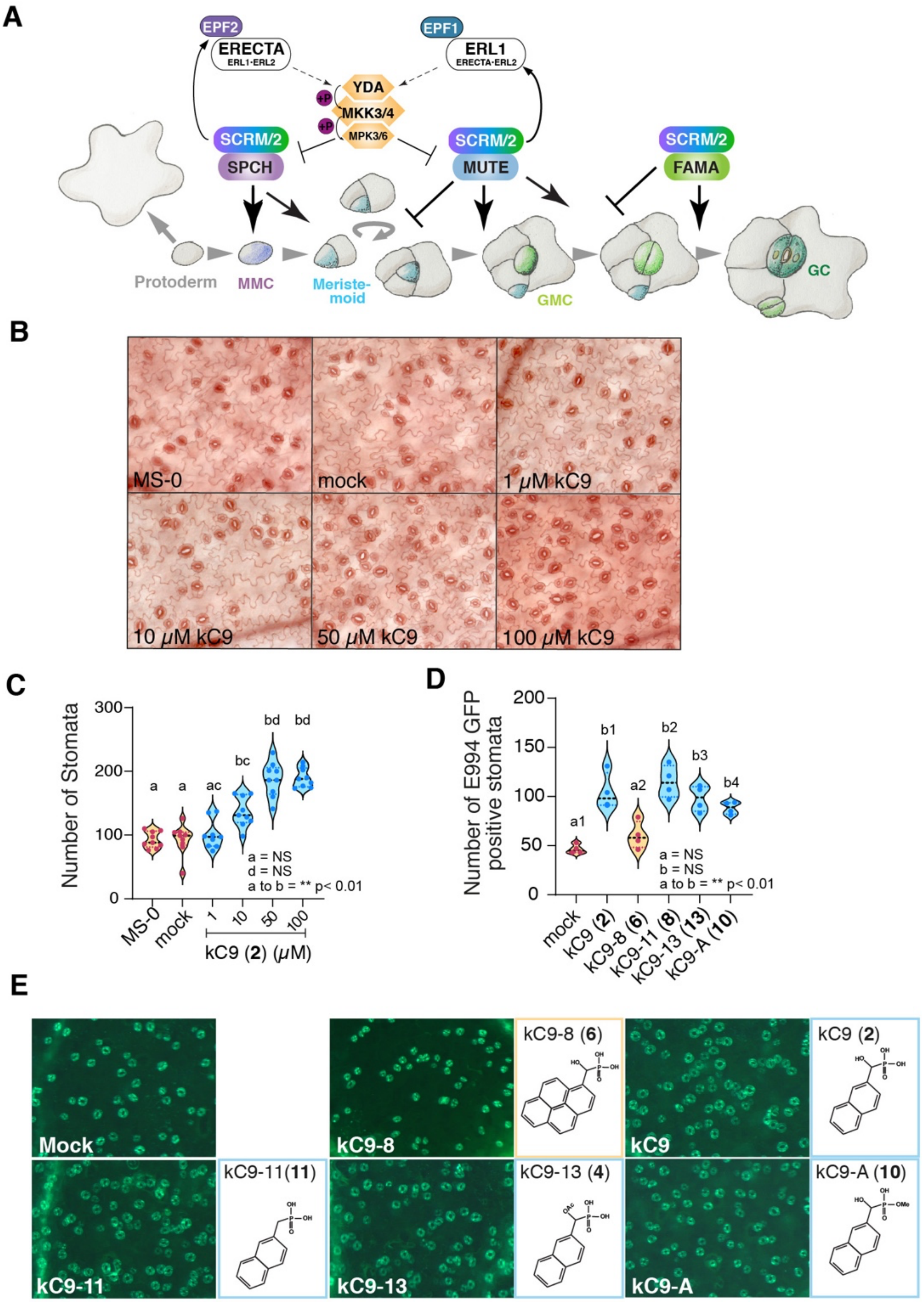

A
EPF2
ERECTA
ERL1·ERL2
EPF1
ERL1
ERECTA·ERL2
YDA
MKK3/4
MPK3/6
+P
SCRM/2
SPCH
SCRM/2
MUTE
SCRM/2
FAMA
Protoderm
MMC
Meriste-moid
GMC
GC
B
MS-0
mock
1 μM kC9
10 μM kC9
50 μM kC9
100 μM kC9
C
Number of Stomata
a = NS
d = NS
a to b = ** p< 0.01
MS-0
mock
kC9 (2) (μM)
D
Number of E994 GFP positive stomata
a = NS
b = NS
a to b = ** p< 0.01
mock
kC9 (2)
kC9-8 (6)
kC9-11 (8)
kC9-13 (13)
kC9-A (10)
E
Mock
kC9-8
kC9-8 (6)
kC9
kC9 (2)
kC9-11
kC9-11(11)
kC9-13
kC9-13 (4)
kC9-A
kC9-A (10)

Figure S1. kC9 and its analogues increasing stomatal development

(**A**) Schematic diagram of the genetic control of stomatal cell state transitions. Key genes are indicated in the cartoon. See main text for details of each step. Arrows show positive regulation, T-bars negative regulation. Dotted arrow line shows potential activation.

(**B**) Microscopy images of 9-day-old abaxial wild-type cotyledon epidermis treated with different concentration of kC9. Specimens are stained with Neutral Red. Note that the bioactivity of kC9, represented as number of stomata, is saturated at a concentration of 50 µM.

(**C**). Quantitative analysis of stomatal density. One-way ANOVA followed by Tukey's HSD analyses were performed. Letters a and d indicate groups that are statistically not different ($p = 0.8999947$), while letter a to b indicate groups which are statistically different, $**p = 0.010053$, as well c to c $**p = 0.0087742$. $n = 9$.

(**D, E**) The effect of different kC9 analogues (50 µM) on *Arabidopsis* upon treatment. While kC9 (compound 2), kC9-11 (compound 11), kC9-13 (compound 4) and kC9-A (compound 10) show a significant increase of stomata expressing the marker E994 upon application, kC9-8 (compound 6) is not significantly different compared to mock treated plants. Shown are quantitative analyses (**D**) and fluorescent light microscopy images (**E**). Structures of the corresponding compounds are provided right next to the representative microscopy images in (**E**). One-way ANOVA followed by Tukey's HSD analyses were performed. Letter a and b indicate groups that are statistically different (except a2 to b4). For exact *P* values see Dataset S1.

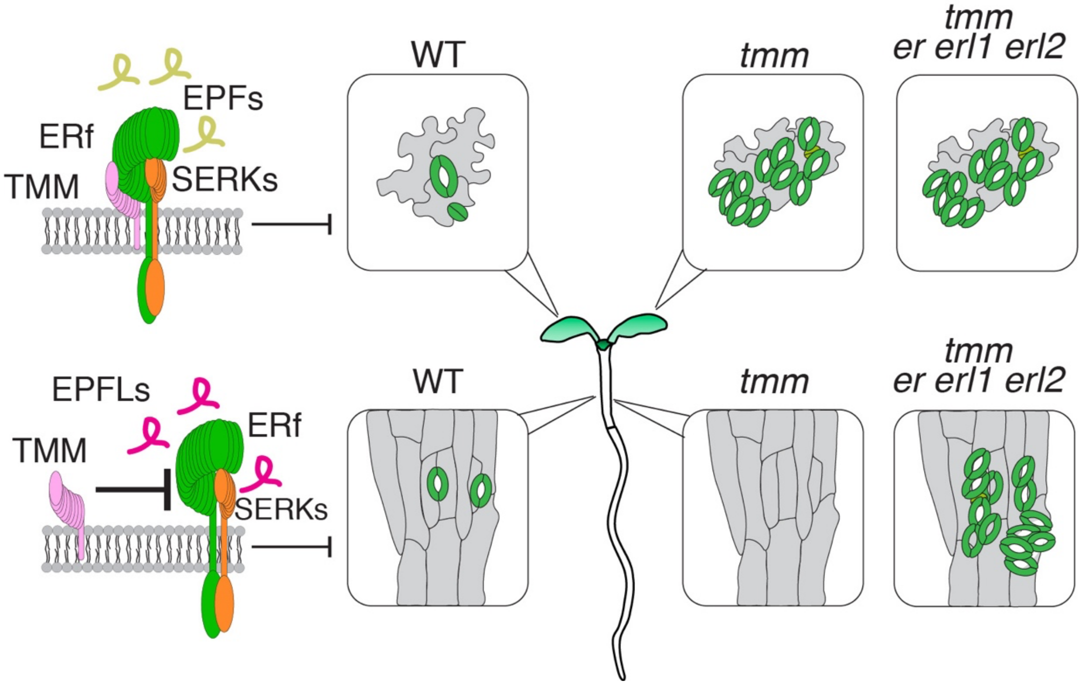

Figure S2. Organ-specific regional effects of the *tmm* mutation and their molecular underpinnings

Shown are schematic diagrams illustrating the organ/region-specific molecular functions of TMM on EPF-ERECTA family (ERf) peptide ligand-receptor system (left) and eventual stomatal phenotypes (right) on cotyledons/leaves (top) and hypocotyls/stems (bottom).

(Top) In cotyledons/leaves, TMM receptor-like protein (pink) preforms a complex with ERf (green) and assists EPF peptide ligand (olive) perception. Ligand-activated ERf forms a receptor complex with SERKs (orange). As such, loss-of-function *tmm* mutant exhibits stomatal clusters, resembling the *er erl1 erl2* triple mutant.

(Bottom) In hypocotyls/stems, TMM 'buffers' EPFL peptides (magenta) enamating from internal cell layers to ensure proper activation of ERf. In the tmm mutant hypocotyls/stems, ERf receptor kinases become overly active, resulting in an epidermis devoid of stomata. Here, ERf are epistatic to TMM, so *tmm er erl1 erl2* quadruple mutant exhibits severe stomatal clusters in stomata-producing epidermal cell files. A seedling cartoon courtesy of Dr. Yvon Jaillais (CNRS, France).

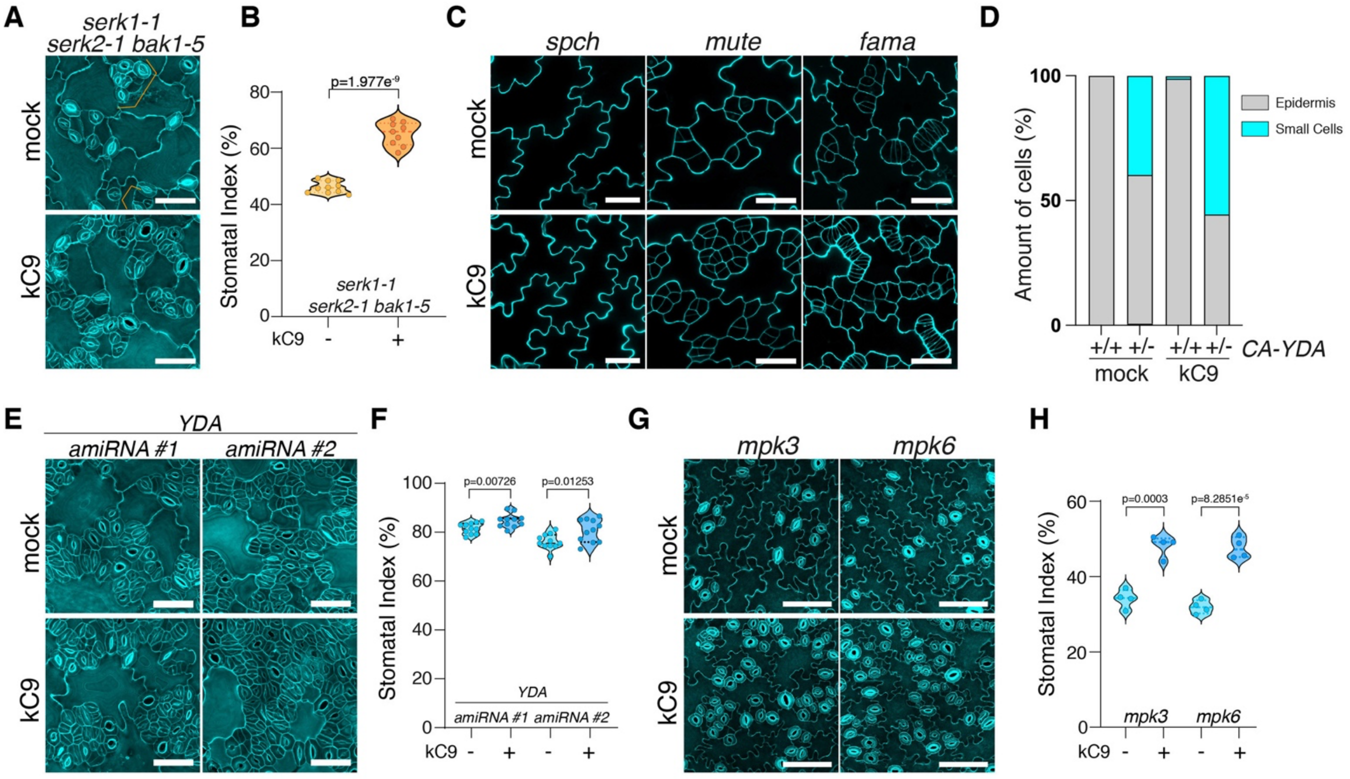

Figure S3. kC9 acts downstream of co-receptors and upstream of master stomatal transcription factors most likely at the signal intermediates MAPKS

(**A, B**) kC9 enhances the stomatal numbers of co-receptor SERK/BAK triple loss-of-function mutants. (**A**) Representative confocal images of co-receptor *serk1-1 serk2-1 bak1-5* mutant, mock (top) and treated with 50 µM kC9 (bottom). Scale bars, 50 µm. (**B**) Stomatal Index, n = 9, Asterisks indicate statistical significance using two-tailed unpaired Student's t-test. ****p = 1.9771E-09

(**C**) kC9's effects on stomatal bHLH mutants. Shown are representative confocal microscopy images of seedling epidermis from *spch* (*spch-3*; no stomata), *mute* (*mute-2*; arrested meristemoids) and *fama* (progressive symmetric divisions) mock (top) or with 50 µM kC9 (bottom). Scale bars, 50 µm for *spch*, 25 µm for *mute* and *fama*. While *spch* mutants do not respond to kC9 treatment, *mute* and *fama* mutants exhibit an increase in mutant cells. However, none of them differentiate stomata. Scale bars, 25 µm.

(**D**). Quantitative analysis of cell types. Epidermal pavement cells, gray; small cells, cyan. Total numbers of cells counted; mock (-/-) n = 104, mock (-/+) n = 171, kC9 (-/-) n = 84, kC9 (-/+) n = 608); pavement cells; mock (-/-) n = 104, mock (-/+) n = 100, kC9 (-/-) n = 82, kC9 (-/+) n = 270, small cells; mock (-/-) n = 0, mock (-/+) n = 71, kC9 (-/-) n = 2, kC9 (-/+) n = 338.

(**E, F**) Representative confocal images of independent transgenic lines (amiRNA#1 and amiRNA#2; expressing an amiRNA against *YDA* (**E**) and quantification of Stomatal Index (%, **F**) treated with either kC9 or mock. SI for *amiRNA#1 YDA* (mock = 81.57 % ± 2.24 %; kC9 = 84.78 ± 2.77 n = 11; * p < 0. 0.007261372) and *amiRNA#2 YDA* (mock = 76.09 % ± 3.05 %; kC9 = 80.70 ± 4.63 n = 11; * p < 0.012535128). Asterisks indicate statistical significance using two-tailed unpaired Student's t-test. Scale bars, 50 μm

(**G, H**) Representative confocal images of different *mpk* related mutants (**G**) and their quantification of the Stomatal Index (%, **H**), treated with either kC9 or mock. Note that kC9 still significantly increases Stomatal Index for both *mpk3* (mock = 34.16 % ± 2.51 %; kC9 = 48.24 ± 2.84 n = 4; ** p < 0.000308133) and *mpk6* single mutant plants (mock = 31.99 % ± 1.81 %; kC9 = 47.57 ± 2.72 n = 4; **** p < 8.28517E-05). Asterisks indicate statistical significance using two-tailed unpaired Student's t-test. Scale bars, 50 μm.

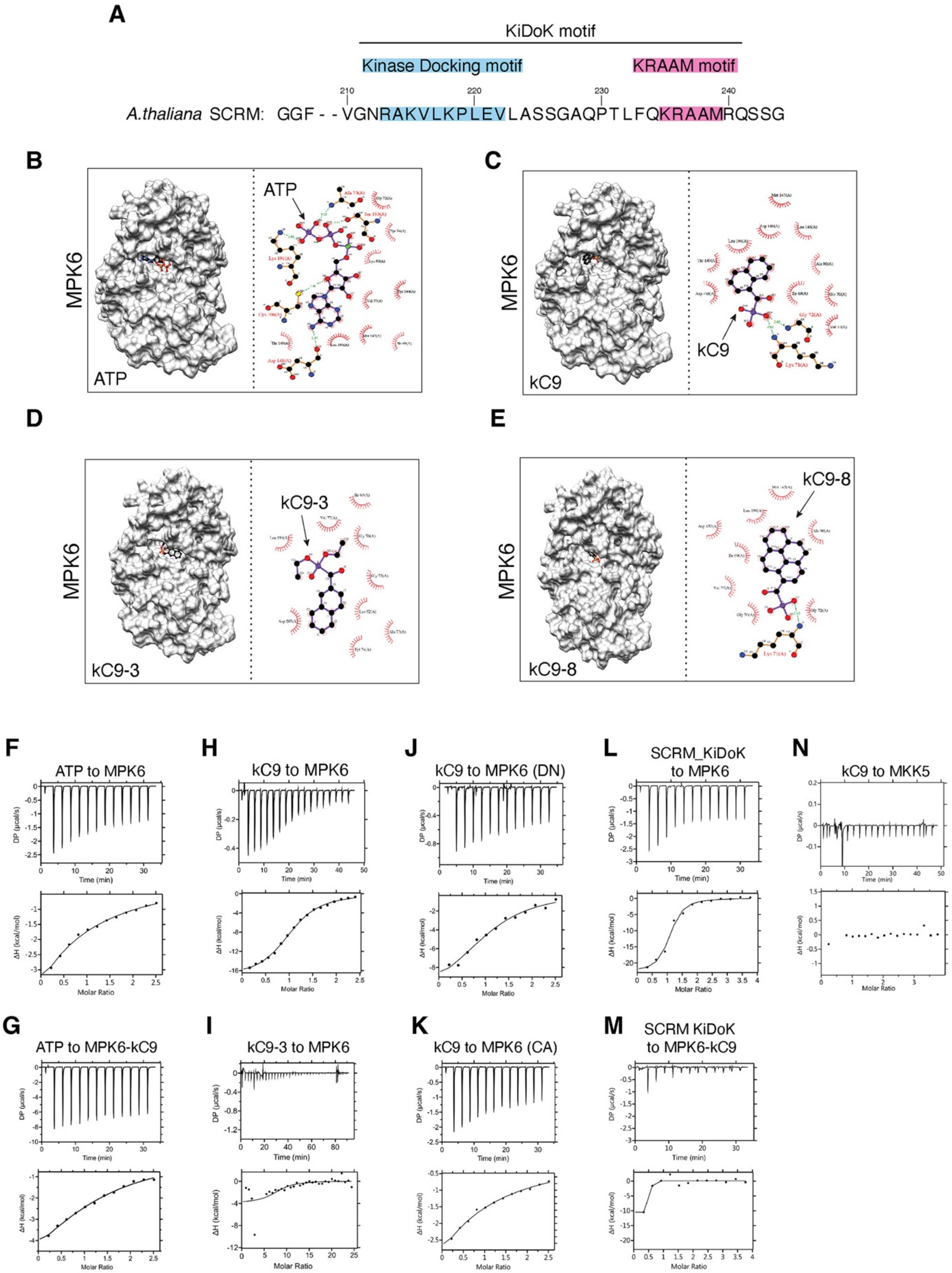
A
KiDoK motif
Kinase Docking motif
KRAAM motif
A.thaliana SCRM: GGF - - VGNRAKVLKPLEVLASSGAQPTLFQKRAAMRQSSG
B
MPK6
ATP
C
kC9
D
kC9-3
E
kC9-8
F
ATP to MPK6
H
kC9 to MPK6
J
kC9 to MPK6 (DN)
L
SCRM_KiDoK to MPK6
N
kC9 to MKK5
G
ATP to MPK6-kC9
I
kC9-3 to MPK6
K
kC9 to MPK6 (CA)
M
SCRM KiDoK to MPK6-kC9
DP (μcal/s)
Time (min)
ΔH (kcal/mol)
Molar Ratio

Figure S4. kC9 binds to MPK6, but not to MKK5, and interferes MPK6 from recruiting its substrate SCRM

(**A**) Amino acid sequence of the SCRM protein kinase docking domain and KRAAM motif.

(**B-E**) Surface representation of structural docking models of the known atMPK6 structure (PDB:6TDL) with ATP (**B**), kC9 (**C**), kC9-3 (**D**), and kC9-8 (**E**). The ligand structures are shown as ball-and-stick representations. The right panel in each figure represents ligand-interacting residues derived from LIGPLOT.

(**F-N**) Isotherms corresponding to the binding of ATP to MPK6 are shown in the absence (**F**) and presence (**G**) of kC9. kC9 binding with wtMPK6 (**H**) and kC9 analogue kC9-3 binding to MPK6 (**I**) are also shown. Additionally, kC9 binding to inactive (**J**) and constitutively active (**K**) forms of MPK6, SCRM KiDOK binding with MPK6 in the absence (**L**) and presence (**M**) of kC9, and kC9 binding to MKK5 (**N**) are presented. The titrations and the integrated data obtained after subtracting the heat of dilution are shown in the upper and lower panels, respectively.

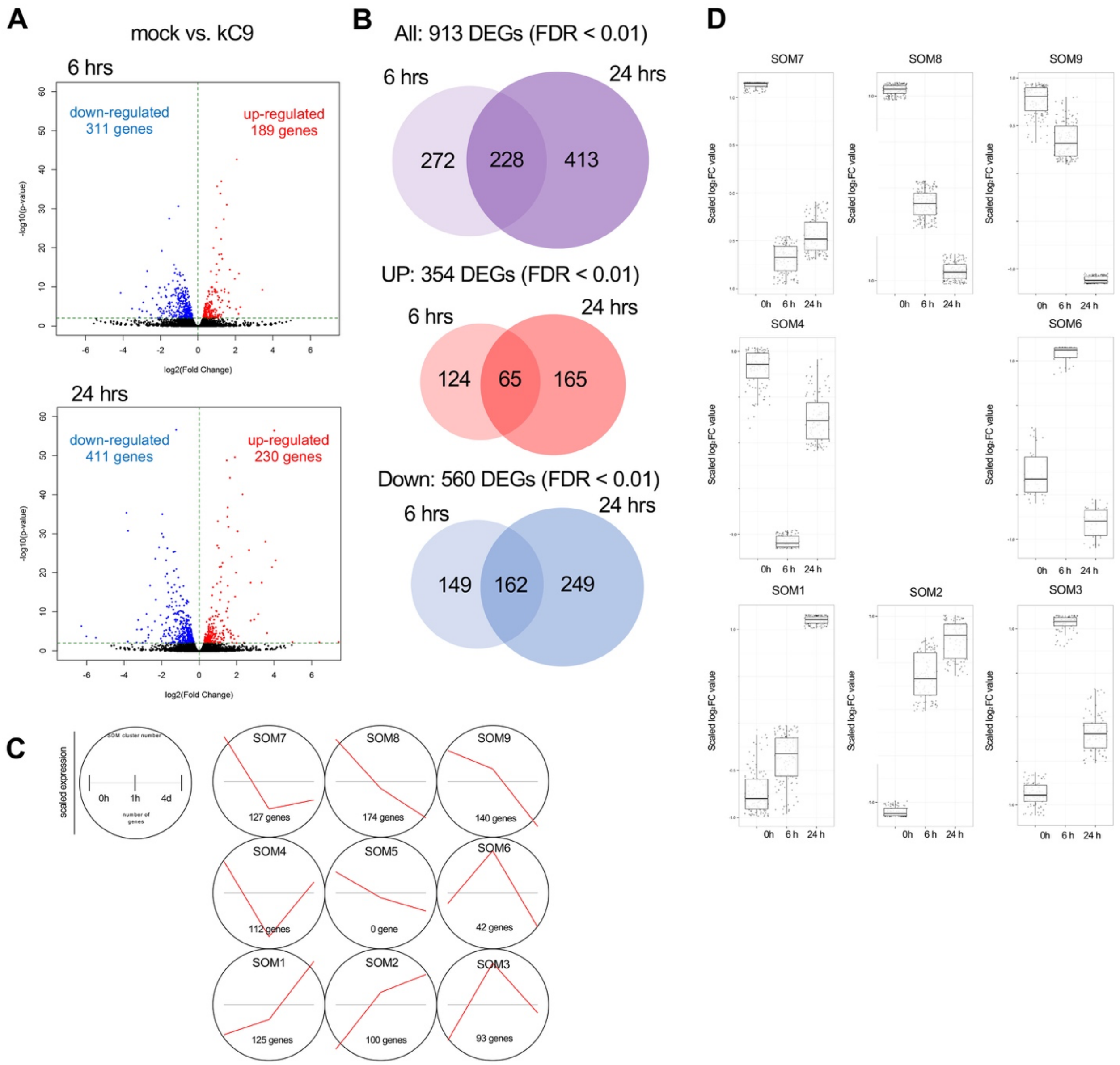

Figure S5. Differentially expressed genes (DEGs) between mock vs. kC9 treatment and SOM cluster analysis

(**A**) Volcano plots showing the expression profile of up- or down-regulated genes by kC9 treatment for 6 hours (top) and 24 hours (bottom). Genes whose expression was significantly altered by kC9 treatment (FDR < 0.01) are included in the plot.

(**B**) Venn diagram showing the number of DEGs (mock vs kC9) that were identified based on significant difference (FDR < 0.01). Plots are for all DEGs (top), Up-regulated DEGs (middle), and Down-regulated DEGs (bottom). Note that the total number of Up- and Down-regulated DEGs

is not exactly equal to that of the total DEGs due to the presence of a gene downregulated in 6 hours but upregulated in 24 hours. For the list of DEGs see Dataset S2.

(**C**) Results of 3 x 3 SOM clustering. Line plots indicate representative expression patterns at 0 hour (virtual), 6 hours and 24 hours after treatment in each cluster. Number of genes assigned to each SOM cluster is shown.

(**D**) Scaled expression between at 0 hour (virtual), 6 hours and 24 hours after treatment are shown. Box plot explanation: upper horizontal line of box, 75th percentile; lower horizontal line of box, 25th percentile; horizontal bar within box, median. For a list of genes in each SOM, see Dataset S2

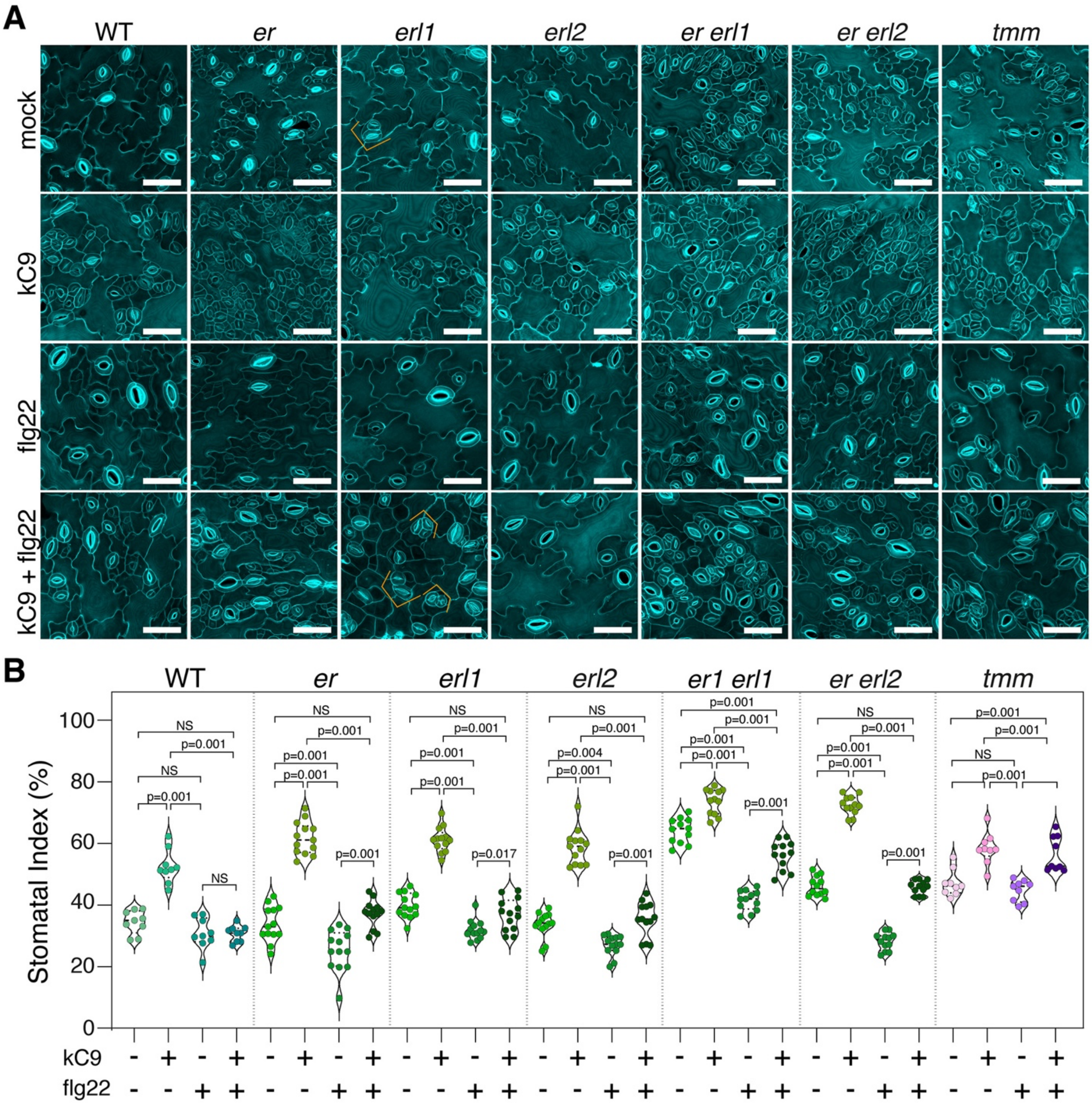

Figure S6. flg22 triggers differential nullifying effects on kC9-treated stomatal development signaling mutants

(**A**) Representative confocal microscopy of cotyledon abaxial seedlings epidermis from 7-days-after-germination: wild-type (WT), *erecta (er)*, *erl1*, *erl1, er erl1, er erl2* or *tmm* single or double mutant combinations (from left to right) treated with either mock, 50 μM kC9, 0.1 μM flg22 or

kC9 + flg22 (from top to bottom). Some data are replotted from Fig. 5D for a better comparison among the genotypes. Scale bars, 50 µm.

(**B**) Stomatal index of wild-type (WT), *erecta (er)*, *erl1*, *erl1, er erl1, er erl2* or *tmm* single or double mutant combinations (from left to right) treated with either mock (-/-), 50 µM kC9 (+/-), 0.1 µM flg22 (-/+) or kC9 + flg22 (+/+). Some data are replotted from Fig. 5E for a better comparison among the genotypes. One-way ANOVA followed by Tukey's HSD analyses were performed. For exact *p* values see Dataset S1.

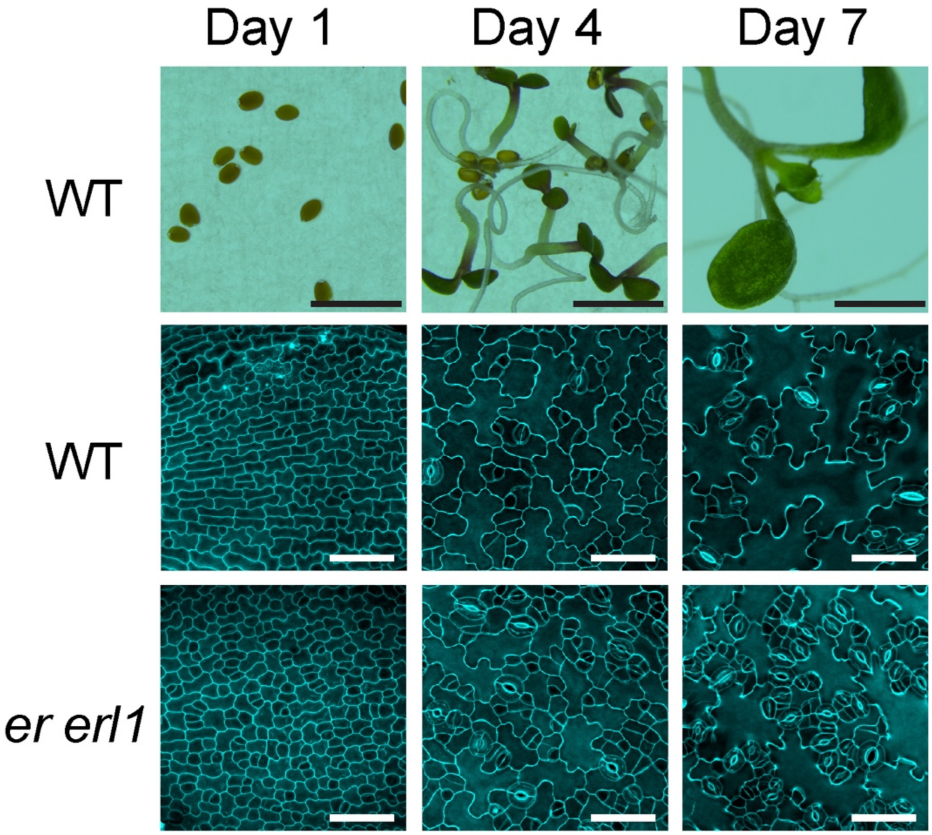

Figure S7. Developmental progression of stomatal lineage cells in wild-type and *er erl1* mutants.

Shown are mock-treated wild-type (WT) seeds/seedlings (top), a representative WT (middle) and *er erl1* (bottom) cotyledon epidermis at Day 1 (left), Day 4 (middle), and Day 7 (right) post germination. Stomatal precursor cells in WT seedlings are actively undergoing proliferative divisions at Day 4. The number of stomatal lineage cells and stomata is intensified in *er erl1* at Day 4 and Day 7, respectively. All seedlings were grown in mock condition, and cotyledon epidermis were stained with propedium iodide to highlight the cell periphery. For imaging Day 1, the embryos were carefully separated from the seed coat using a stereomicroscope. Scale bars, 2 mm (whole seedlings, top); 50 µm (middle and bottom).

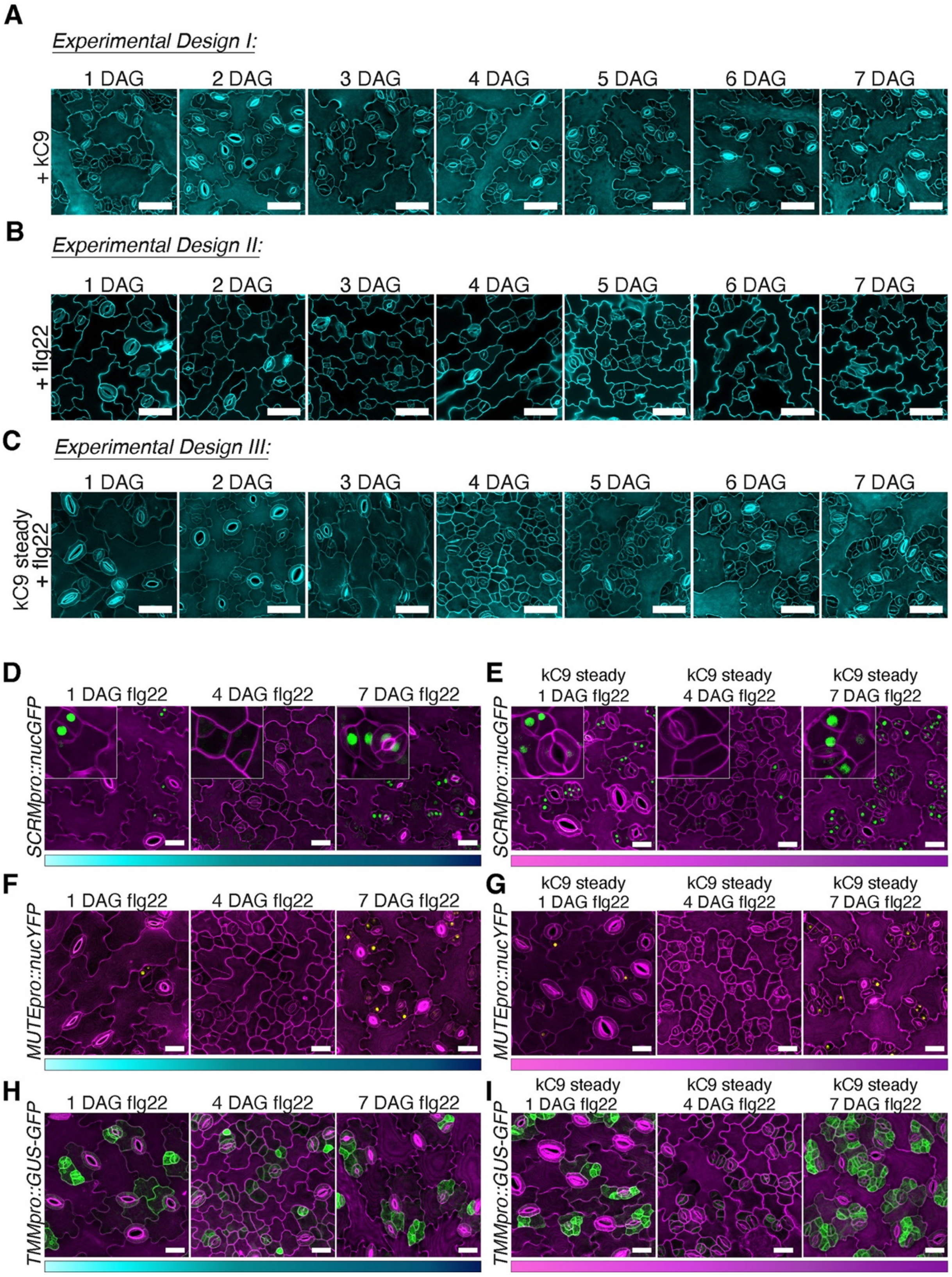
A
Experimental Design I:
1 DAG
2 DAG
3 DAG
4 DAG
5 DAG
6 DAG
7 DAG
+ kC9
B
Experimental Design II:
+ flg22
C
Experimental Design III:
kC9 steady + flg22
D
1 DAG flg22
4 DAG flg22
7 DAG flg22
SCRMpro::nucGFP
E
kC9 steady 1 DAG flg22
kC9 steady 4 DAG flg22
kC9 steady 7 DAG flg22
F
MUTEpro::nucYFP
G
H
TMMpro::GUS-GFP
I

Figure S8. Narrow developmental window of flg22 action to inhibit stomatal-lineage identity
(**A-C**) Representative confocal microscopy of cotyledon abaxial wild-type (WT) seedlings epidermis from 7-days-after-germination (DAG) treated on different days as indicated with either 50 µM kC9 (**A**, *Experimental Design I*), 0.1 µM flg22 (**B**, *Experimental Design II*) or 0.1µM flg22 where kC9 steadily was present for 7 days (**C**, *Experimental Design III*). Scale bars, 50 µm. Corresponds to Figure 6A-G.

(**D-I**) Representative confocal microscopy of cotyledon abaxial seedlings epidermis from 7-days-after-germination (7 DAG) expressing expressing the stomatal transcriptional reporter lines: *SCRMpro::nucGFP* (**D, E**); *MUTEpro::nucYFP* (**F, G**); and *TMM::proGUS-GFP* (**H, I**) treated on 1, 4 or 7 DAG with 0.1 µM flg22 in the presence (**E, G, I**) or absence (**D, F, H**) of kC9. Note the substantially diminished fluorescent signals when flg22 was applied on 4 DAG. Scale bars, 25 µm.

Table S1. Thermodynamic parameters for the binding of kC9 binding with wild type MPK6, constitutively active MPK6 and inactive version of MPK6, and MKK5, ATP and SCRM KiDoK peptide binding with MPK6 in presence and absence of kC9

	Kd	n	ΔH	-TΔS	ΔG
kC9-MPK6	4.9 ± 0.6 μM	1.0	-15.5 ± 0.6	3.2 ± 0.6	-12.3 ± 0.2
kC9-$MPK6_{D218G, E222A}$	58.0 ± 13 μM	1.1	-2.5 ± 0.2	1.2 ± 0.3	-1.3 ± 0.2
kC9-$MPK6_{K92M, K92R}$	9.8 ± 0.8 μM	0.97	-8.1 ± 0.3	2.3 ± 0.4	-5.8 ± 0.2
kC9-MKK5	NB	NB	NB	NB	NB
kC9-3 to MPK6	NB	NB	NB	NB	NB
ATP to MPK6	1.2 ± 0.3 μM	1.0	-2.5 ± 0.2	1.1 ± 0.2	-1.4 ± 0.2
ATP to MPK6+kC9	3.1 ± 0.4 μM	1.2	-3.1 ± 0.4	2.6 ± 0.3	-0.5 ± 0.2
SCRM KDock to MPK6	43.4 ± 2.1 nM	1.0	-22.5 ± 0.2	10.2 ± 0.3	-12.3 ± 0.1
SCRM KDock to MPK6+kC9	1403 ± 40 nM	0.85	-10.1 ± 0.2	6.2 ± 0.2	-3.9 ± 0.2

Table S2. Kinetic- and binding constants for kC9 binding with wild type MPK6, constitutively active MPK6 and inactive version of MPK6, and SCRM KiDoK peptide binding with MPK6 in presence and absence of kC9

	k_{on} ($M^{-1}s^{-1}$)	k_{off} (s^{-1})	K_D	K_D (SS)
kC9-MPK6	3.02×10^3	1.75×10^{-2}	5.8 ± 0.5 μM	5.6 ± 0.6 μM
kC9-$MPK6_{D218G, E222A}$	3.36×10^3	2.52×10^{-1}	75.3 ± 9.5 μM	65 ± 6.3 μM
kC9-$MPK6_{K92M, K92R}$	4.35×10^3	4.84×10^{-2}	11.1 ± 0.3 μM	9.8 ± 1.1 μM
kC9-3 to MPK6	ND	ND	ND	ND
ATP-MPK6	8.92×10^3	1.25×10^{-2}	1.4 ± 0.3 μM	1.8 ± 0.2 μM
ATP-MPK6+kC9	4.97×10^3	1.74×10^{-1}	3.5 ± 1.2 μM	5.6 ± 0.4 μM
SCRM KDock to MPK6	7.78×10^5	3.27×10^{-2}	42. 2 ± 0.7 nM	40.1 ± 2.5 nM
SCRM KDock to MPK6+kC9	1.70×10^4	1.78×10^{-2}	1048.5 ± 4.3 nM	856.5 ± 16.0 nM

Table S3. Binding energies for MPK6-ATP, MPK6-kC9, MPK6-kC9-3 and MPK6-kC9-8

**MPK6-ATP**							
#Cluster	haddock score	rmsd (Å)	Nstruc	vdw (au)	Eelec (au)	BSA (Å)	#dH
Cluster1	-30.996	0.436	187	-11.75	-120.91	564.94	-15.557
Cluster2	-49.619	0.413	13	-14.23	-127.16	567.733	-27.564
**MPK6-kC9**							
#Cluster	haddock score	rmsd (Å)	Nstruc	Evdw	Eelec	BSA (Å)	#dH
Cluster1	-32.496	0.649	174	-6.9	-136.68	443.286	-100.545
Cluster2	-25.091	0.843	18	-6.68	-97.81	467.278	-67.41
Cluster3	-25.471	0.485	8	-9.94	-127.61	458.484	-101.874
**MPk6-kC9-3**							
#Cluster	haddock score	rmsd (Å)	Nstruc	Evdw	Eelec	BSA (Å)	#dH
Cluster1	-15.139	0.291	20	-26	-69.75	743.813	43.523
Cluster2	-17.289	0.328	18	-30.41	-34.73	735.184	68.434
Cluster3	-1.289	0.246	9	-30.49	-125.88	777.198	-19.181
Cluster4	-11.676	0.303	7	-23.16	-52.19	708.651	58.652
Cluster5	-6.041	0.368	6	-25.42	-86.73	722.218	25.025
Cluster6	-7.942	0.277	5	-30.3	-118.35	768.544	-10.747
**MPK6-kC9-8**							
#Cluster	haddock score	rmsd (Å)	Nstruc	Evdw	Eelec	BSA (Å)	#dH
Cluster1	-15.428	0.456	12	-20.64	-65.35	595.707	8.46
Cluster2	-5.454	0.432	8	-22.51	46.63	603.367	123.27
Cluster3	-11.511	0.192	7	-14.01	-95.26	579.837	-12.66
Cluster4	-4.278	0.266	7	-16.55	-52.45	584.426	23.158
Cluster4	-5.031	0.477	6	-14.22	-37.5	568.866	45.624
Cluster5	-11.593	0.428	5	-17.67	-65.26	585.887	11.387

Table S4. List of plasmids used in this study

Plasmid ID	Name	Insert	Vector	Bac R
pAHs022	pDONOR_P1P4_cMPK6 w/o STOP	cMPK6 w/o STOP	pDONR_P1P4	Kan
pAHs039	pDONOR_P3P2_cSCRM w/o STOP	cSCRM w/o STOP	pDONR_P3P2	Kan
pAHs041	pDONOR_P3P2_cSPCH w/o STOP	cSPCH w/o STOP	pDONR_P3P2	Kan
pAHs043	pBiFCt-2in1_35S:SCRM-nYFP_35S:MPK6-cYFP, 35S:RFP	cMPK6 w/o STOP, cSCRM w/o STOP	V259	Spec
pAHs045	pBiFCt-2in1_35S:SPCH-nYFP_35S:MPK6-cYFP, 35S:RFP	cMPK6 w/o STOP, cSPCH w/o STOP	V259	Spec
pKM017	pGEX4T-1-MKK5	MKK5_WT	pGEX4T-1	Amp
pKM018	pGEX4T-1-MKK5_DD	MKK5_DD	pGEX4T-1	Amp
pAP186	pGEX4T-1-MPK6 ΔN (1-28)	MPK6 ΔN (1-28)	pGEX4T-1	Amp
pAHs053	pGEX-4T-1_deltaN-cMPK6_K92M_K93R	deltaN-cMPK6_K92M_K93R	pGEX-4T-1	Amp
pAHs055	pGEX-4T-1_deltaN-cMPK6_D218G_E222A	deltaN-cMPK6_D218G_E222A	pGEX-4T-1	Amp

Table S5. List of oligo DNA primers and their sequences used in this study

ID/allele	Description	Sequences	Purpose
AHs001	Lba1	TGG TTC ACG TAG TGG GCC ATC G	genotyping
AHs002	Lbb1	ATTTTGCCGATTTCGGAAC	genotyping
AHs003	LBG_KAT	ATAATAACGCTGCGGACATCTACATTTT	genotyping
AHs004	M13F	GTAAAACGACGGCCAGT	sequencing
AHs005	M13R	CAGGAAACAGCTATGAC	sequencing
AHs006	pENTR_F	GTTTCTACAAACTCTTCCTG	sequencing
er105	ERg2248	AAGAAGTCATTCAAAGATGTGA	genotyping
	ERg3016rc	AGAATTTCCAGGTTTGGAATCTGT	genotyping
	er-105rc	AGCTGACTATACCCGATACTGA	genotyping
erl1-2	ERL1g3659	GAGCTTGGACATATAATCAATC	genotyping
	ERL1g4411rc	CCGGAGAGATTGTTGAAGGA	genotyping
	ERL1K3036	GTCACGTCTCAGCTATTTGTAAGCTTGTT	genotyping
	JL202	CATTTTATAATAACGCTGCGGACATCTAC	genotyping
erl2-1	ERL2 2166	GCCTATTCCACCAATACTTG	genotyping
	ERL2 3182rc	ACAAATCTGAGAGAGTTAATGCAAAGCAG	genotyping
	JL202	CATTTTATAATAACGCTGCGGACATCTAC	genotyping
tmm-KO	TMM391	CTCAAACACCTCAAAGCCTT	genotyping
	TMM 1254.rc	GAACCGAATGCATCATCCAAGTCACT	genotyping
	lBb1.3	ATTTTGCCGATTTCGGAAC	genotyping
epf2-1	EPF2.1.cDNA.xhoI	CACCCTCGAG-ATGACGAAGTTTGTACGCAAGT	genotyping
	EPF2.360.cDNA.ecoRI.rc2	CGGAATTCTAGCTCTAGATGGCACGTGATAG	genotyping
	lBb1.3	ATTTTGCCGATTTCGGAAC	genotyping
spch-3	53210 1.GW	CACCATGCAGGAGATAATACCG	genotyping
	53210 1281.rc	AACCTGAAGAATCTCAAGAGCC	genotyping
	SAIL LB3	TAGCATCTGAATTTCATAACCAATCTCGATACAC	genotyping
mute-2	mute 1	CACCATGTCTCACATCGCTGTTG	genotyping
	bHLH 266.rc	TAATCTTGAATCAACCTC	genotyping
	FST-RB3	CTACAAATTGCCTTTTCTTATCGAC	genotyping
AHs083	attB3_SCRM	GGGG ACA ACT TTG TAT AAT AAA GTT GTA atg GGTCTTGACGGAAACAAT	plasmid construction
AHs084	attB2_SCRM	GGGG AC CAC TTT GTA CAA GAA AGC TGG GTT GATCATACCAGCATACCCTGCT	plasmid construction
AHs087	attB3_SPCH	GGGG ACA ACT TTG TAT AAT AAA GTT GTA atg CAGGAGATAATACCGGATT	plasmid construction
AHs088	attB2_SPCH	GGGG AC CAC TTT GTA CAA GAA AGC TGG GTT GCAGAATGTTTGCTGAATTTGT	plasmid construction
AHs073	attB1_MPK6	GGGG ACA AGT TTG TAC AAA AAA GCA GGC TTA atg GACGGTGGTTCAGGTCAA	plasmid construction
AHs074	attB4_MPK6	GGGG AC AAC TTT GTA TAG AAA AGT TGG GTG TTGCTGATATTCTGGATTGAAAGCA	plasmid construction
AP90	MPK6 PGEX4T NotI R	CATG GCGGCCGC CTA TTG CTG ATA TTC TGG ATT	plasmid construction
AP93	MPK6 Delta N BamH1 F	CATG GGATCC CAG ATG CCT GGG ATT GAG AAT	plasmid construction
SK24	MKK5 WT Not1	CTGCGGCCGCGCCATGGATGAAACCGATTCAATC	plasmid construction
SK25	MKK WT BamH1	ATGGATCCATTCCTAAGAGGCAGAAGGAAGAGGA	plasmid construction
SK26	MKK5_DD PGEX4T BamH1	CAT GGATCC ATT CCT AAG AGG CAG AAG GAA GAG GAC [illegible]	plasmid construction
SK27	MKK5_DD PGEX4T Not1 R	CATGCGGCCCGCGCCATGGATGAAACC GAT TCA ATC TCC TTC T	plasmid construction
AHs091	MPK6_K92M_K93R_F	AGAGCGTTGCGATTATGAGAATTGCTAACGCTTT	plasmid construction
AHs092	MPK6_K92M_K93R_R	AAAGCGTTAGCAATTCTCATAATCGCAACGCTCT	plasmid construction
AHs095	MPK6_D218G_E222A_F	ACTTCTGAGAGTGGTTTCATGACTGCATATGTTGTCACGA	plasmid construction
AHs096	MPK6_D218G_E222A_R	TCGTGACAACATATGCAGTCATGAAACCACTCTCAGAAGT	plasmid construction

Section S1. Chemical Synthesis and NMR Analysis

General

All reagents and solvents were purchased from commercial suppliers and used without further purification.

The ^{1}H, $^{13}C\{^{1}H\}$, $^{31}P\{^{1}H\}$ NMR were recorded on a JEOL JNM-ECA500 (500 MHz for ^{1}H, 125 MHz for ^{13}C, 202 MHz for ^{31}P) spectrometer. Chemical shifts were reported in ppm (δ), and coupling constants were reported in Hz. ^{1}H and ^{13}C-resonances were referenced to solvent residual peaks for $CDCl_3$ (^{1}H, 7.26 ppm), CD_3OD (^{1}H, 3.31 ppm), DMSO-d_6 (^{1}H, 2.50 ppm), $CDCl_3$ (^{13}C, 77.16 ppm), CD_3OD (^{13}C, 49.00 ppm), and DMSO-d_6 (^{13}C, 39.52ppm). ^{31}P-resonance was referenced to 85% phosphoric acid (^{31}P, 0.00 ppm) as external standard. Multiplicity and qualifier abbreviations are as follows: s = singlet, d = doublet, t = triplet, m = multiplet, br = broad, doublet of doublets (dd). Spectra were processed by Delta NMR software (JEOL).

High resolution mass analyses (HRMS) were submitted to the Mass Spectrometry Laboratory (Molecular Structure Characterization Unit) at RIKEN. For crude analysis, ultra high-performance liquid chromatography-mass spectrometry (UPLC/MS) was performed on a SHIMADZU LCMS-2020 equipped with a reverse phase C18 column (2.7 µm particle size, 2.1 x 100 mm) and a API/ESI mass spectrometry detector, and UV detector.

Thin-layer chromatography was performed on Merck 60 F254 precoated silica gel plates. Column chromatography was performed on Biotage Isorela One (Biotage Sfär C18 Duo column or Biotage Sfär Duo column)

Scheme 1. Synthesis of kC9 and its derivatives

kC9 and its derivatives were synthesized from commercially available 2-naphthaldehyde, 1-pyrenecarboxaldehyde, and 2-bromomethyl naphthalene. Phosphonic acid diesters (**1**, **5**, **7**, and **9**) were prepared by nucleophilic addition or substitution reactions with dialkyl phosphites. Acetylation of a-hydroxyl group of **1** led to **3**. Lewis acid treatment of phosphonic acid diesters (**1**, **3**, **5**, **7**, and **9**) successfully provided phosphonic acids (**2**, **4**, **6** and **8**) and phosphonic acid monoester (**10**), respectively.

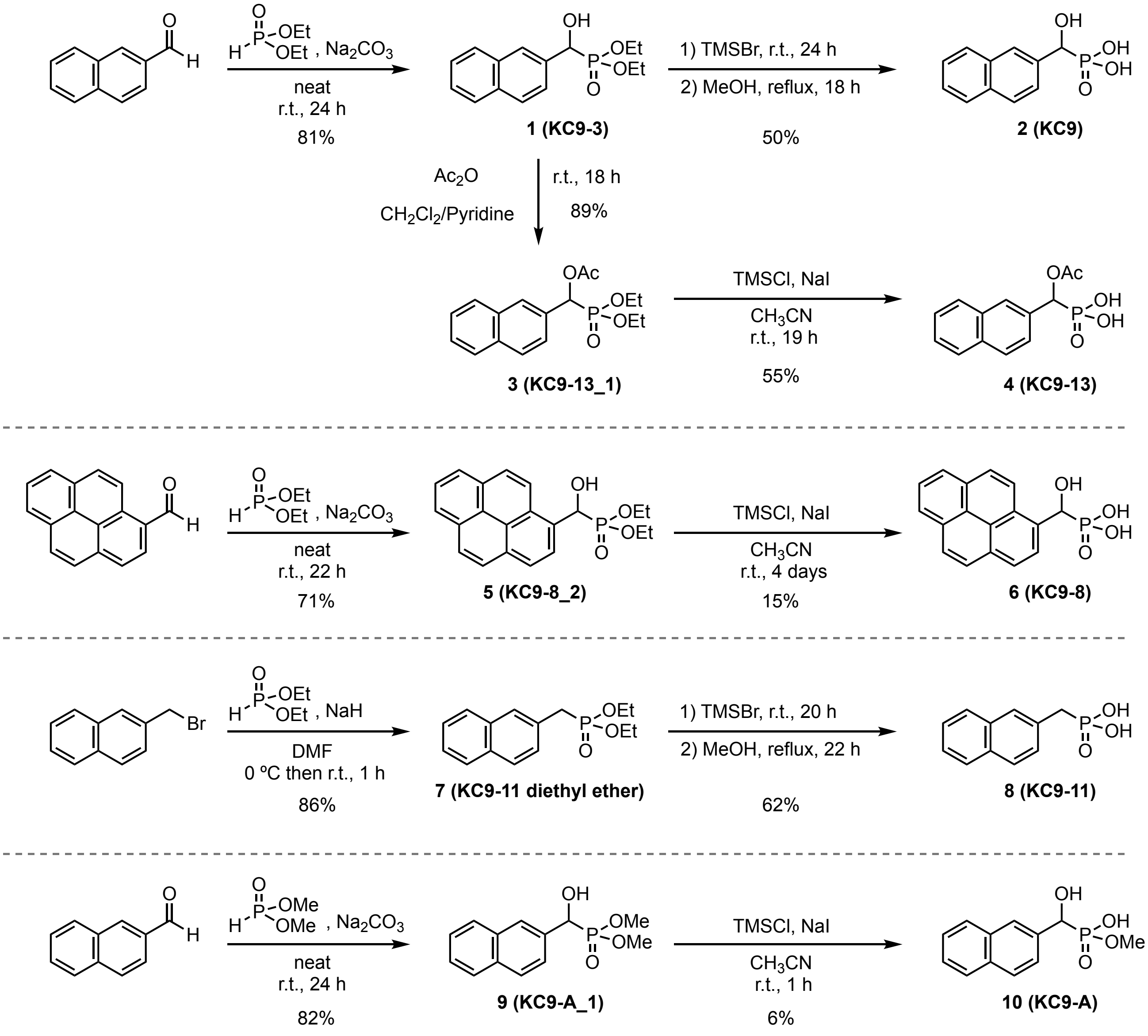

1.1: Synthesis of diethyl (hydroxy(naphthalen-2-yl)methyl)phosphonate 1

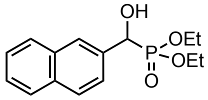

1 (KC9-3)

To a suspension of 2-naphthaldehyde (1.56 g, 10.0 mmol) in diethyl phosphite (2.6 mL, 20.0 mmol) was added Na_2CO_3 (2.12 g, 20.0 mmol) under solvent-free conditions at room temperature. The mixture was stirred for 24 hours at room temperature, after which the reaction mixture was diluted with EtOAc and washed with water. The organic layer was dried over Na_2SO_4, filtrated, and concentrated *in vacuo*. The residue was applied for column chromatography (Biotage Sfär D column, 0–8% $CH_3OH/CHCl_3$ over 10 column volumes) to afford **1** as a pale yellow sticky solid (2.38 g, 81%); ^1H NMR (500 MHz, $CDCl_3$) δ 7.95 (s, 1H), 7.83–7.81 (m, 3H), 7.60 (d, J = 7.0 Hz, 1H), 7.49–7.46 (m, 2H), 5.19 (d, J = 11.0 Hz, 1H), 4.09–3.96 (m, 4H), 3.75 (brs, 1H), 1.25 (t, J = 7.5 Hz, 3H), 1.20 (t, J = 7.5 Hz, 3H); ^{13}C NMR (125 MHz, $CDCl_3$) δ 134.2 (d, J = 2.4 Hz), 133.2, 128.2, 128.0, 127.8, 126.3, 126.2, 125.0 (d, J = 4.9 Hz), 71.1 (d, J = 159 Hz), 63.4 (d, J = 7.1 Hz), 63.3 (d, J = 7.1 Hz), 16.5 (d, J = 3.6 Hz), 16.5 (d, J = 3.6 Hz); ^{31}P NMR (202 MHz, $CDCl_3$) δ 22.0; HRMS (ESI): calculated for $C_{15}H_{19}O_4NaP$ ($[M + Na]^+$) requires m/z = 317.0919, found 317.0917. *Two carbon signals are missing due to signal overlap.

1.2: Synthesis of (hydroxy(naphthalen-2-yl)methyl)phosphonic acid 2

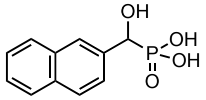

2 (KC9)

TMSBr (0.20 mL, 1.50 mmol) was slowly added to **2** (150 mg, 0.501 mmol) under solvent-free conditions at room temperature. After being stirred for 24 hours at room temperature under N_2, the remaining TMSBr was removed *in vacuo*. This residue was dissolved in MeOH (1.5 mL) and refluxed for 18 hours under N_2, after which volatile compounds were removed *in vacuo*. The resulting solid was filtrated and washed with cold acetone to afford **2** as a white solid (61.2 mg, 50%); ^1H NMR (500 MHz, DMSO-d_6) δ 7.90–7.86 (m, 3H), 7.83 (d, J = 8.5 Hz, 1H), 7.58 (d, J = 8.5 Hz, 1H), 7.50–7.45 (m, 2H), 5.95 (brs, 1H), 4.85 (d, J = 14.0 Hz, 1H); ^{13}C NMR (125 MHz, DMSO-d_6) δ 137.9, 132.6, 132.3, 127.7, 127.5, 126.8, 126.1 (d, J = 3.6 Hz), 125.9, 125.7 (d, J = 7.3 Hz), 125.6, 70.6 (d, J = 159 Hz); ^{31}P NMR (202 MHz, DMSO-d_6) δ 18.7; HRMS (ESI): calculated for $C_{11}H_{10}O_4P$ ($[M – H]^–$) requires m/z = 237.0317, found 237.0319.

1.3: Synthesis of diethyl (acetoxy(naphthalen-2-yl)methyl)phosphonic acid 3

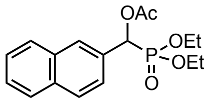

3 (KC9-13_1)

To a solution of **2** (200 mg, 0.679 mmol) in CH_2Cl_2 (3.4 mL) and pyridine (3.4 mL) was added Ac_2O (0.26 mL, 2.72 mmol) at room temperature. The mixture was stirred for 18 hours at room temperature, after which the reaction mixture was diluted with EtOAc and washed with water. The organic layer was dried over Na_2SO_4, filtrated, and concentrated *in vacuo*. The residue was applied for column chromatography (Biotage Sfär D column, 10–100% EtOAc/Hexane over 10 column volumes) to afford **3** as a colorless oil (204 mg, 89%); ^{1}H NMR (500 MHz, $CDCl_3$) δ□7.95 (s, 1H), 7.85–7.82 (m, 3H), 7.61 (d, J = 9.0 Hz, 1H), 7.51–7.47 (m, 2H), 6.31 (d, J = 14.0 Hz, 1H), 4.13–4.02 (m, 3H), 3.98–3.90 (m, 1H), 2.20 (s, 3H), 1.27 (t, J = 7.0 Hz, 3H), 1.20 (t, J = 7.0 Hz, 3H); ^{13}C NMR (125 MHz, $CDCl_3$) δ□169.4 (d, J = 9.5 Hz), 133.4, 133.1, 131.0 (d, J = 2.4 Hz), 128.3, 128.3, 127.8, 127.5 (d, J = 8.4 H), 126.6, 126.5, 127.5 (d, J = 4.8 H), 71.2 (d, J = 169 Hz), 63.5 (d, J = 7.3 Hz), 63.5 (d, J = 7.3 Hz), 21.0, 16.5 (d, J = 6.0 Hz), 16.4 (d, J = 6.0 Hz); ^{31}P NMR (202 MHz, $CDCl_3$) δ□18.4; HRMS (ESI): calculated for $C_{17}H_{21}O_5NaP$ ($[M + Na]^+$) requires m/z = 359.1024, found 359.1020.

1.4: Synthesis of (acetoxy(naphthalen-2-yl)methyl)phosphonic acid 4

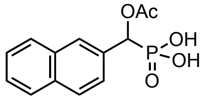

4 (KC9-13)

TMSCl (0.19 mL, 1.49 mmol) was added to a mixture of **3** (100 mg, 0.297 mmol) and NaI (223 mg, 1.49 mmol) in CH_3CN (3 mL) at room temperature. The reaction mixture was stirred for 19 hours at room temperature, after which the reaction was quenched with an excess amount of MeOH and concentrated *in vacuo*. The residue was applied for column chromatography (Biotage Sfär C18 Duo column, 10–60% CH_3CN/H_2O over 10 column volumes) to afford **4** as a colorless oil (45.4 mg, 55%); ^{1}H NMR (500 MHz, DMSO-d_6) δ□7.94–7.88 (m, 4H), 7.56–7.50 (m, 3H), 5.99 (d, J = 14.0 Hz, 1H), 2.15 (s, 3H); ^{13}C NMR (125 MHz, DMSO-d_6) δ□169.5 (d, J = 9.5 Hz), 133.2, 132.5, 127.9, 127.5 (d, J = 2.4 Hz), 126.3, 126.3, 126.2 125.7 (d, J = 3.6 H), 71.7 (d, J = 161 Hz), 20.9; ^{31}P NMR (202 MHz, DMSO-d_6) δ□13.5; HRMS (ESI): calculated for $C_{13}H_{12}O_5P$ ($[M - H]^-$) requires m/z = 279.0422, found 279.0424. *Two carbon signals are missing due to overlapping with signal overlap.

1.5: Synthesis of diethyl (hydroxy(pyren-1-yl)methyl)phosphonate 5

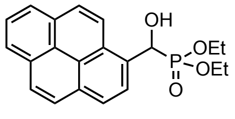

5 (KC9-8_2)

To a suspension of 1-pyrenecarboxaldehyde (691 mg, 3.00 mmol) in diethyl phosphite (0.77 mL, 6.00 mmol) was added Na_2CO_3 (636 mg, 6.00 mmol) under solvent-free conditions at room temperature. The mixture was stirred for 22 hours at room temperature, after which the reaction mixture was diluted with $CHCl_3$ and small amount of MeOH and washed with water. The organic layer was dried over Na_2SO_4, filtrated, and concentrated *in vacuo*. The residue was applied for column chromatography (Biotage Sfär D column, 0–8% $CH_3OH/CHCl_3$ over 10 column volumes) to afford **5** as a pale yellow solid (791 mg, 71%); ^{1}H NMR (500 MHz, DMSO-d_6) δ 8.51 (d, J = 9.5 Hz, 1H), 8.35–8.30 (m, 4H), 8.21 (d, J = 9.0 Hz, 1H), 8.19–8.17 (m, 2H), 8.08 (t, J = 8.0 Hz, 1H), 6.55 (d, J = 17.5 Hz, 1H), 6.05 (d, J = 14.0 Hz, 1H), 4.03–3.97 (m, 2H), 3.92–3.87 (m, 1H), 3.79–3.73 (m, 1H), 1.17 (t, J = 8.0 Hz, 3H), 0.994 (t, J = 8.0 Hz, 3H); ^{13}C NMR (125 MHz, DMSO-d_6) δ□132.6, 130.8, 130.3 (d, J = 2.5 Hz), 130.2, 127.9 (d, J = 7.1 Hz), 127.4, 127.3, 126.9, 126.4 (d, J = 4.8 Hz), 126.2, 125.3, 125.1, 124.6 (d, J = 2.4 Hz), 124.1, 123.8, 123.7, 66.5 (d, J = 163 Hz), 62.3 (d, J = 7.1 Hz), 61.9 (d, J = 7.1 Hz), 16.4 (d, J = 4.8 Hz), 16.2 (d, J = 4.8 Hz); ^{31}P NMR (202 MHz, DMSO-d_6) δ□22.3; HRMS (ESI): calculated for $C_{21}H_{21}O_4NaP$ ([M + Na]$^+$) requires m/z = 391.1075, found 391.1071.

1.6: Synthesis of (hydroxy(pyren-1-yl)methyl)phosphonic acid 6

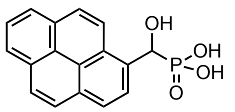

6 (KC9-8)

TMSCl (68.6 μL, 0.543 mmol) was added to a mixture of **5** (50 mg, 0.136 mmol) and NaI (81.0 mg, 0.540 mmol) in CH_3CN (1.5 mL) at room temperature. The resulting solid was filtrated and washed with H_2O. The residue was applied for column chromatography (Biotage Sfär C18 Duo column, 10–60% CH_3CN/H_2O over 12 column volumes) to afford **6** as a pale yellow solid (12.7 mg, 15%); ^{1}H NMR (500 MHz, DMSO-d_6) δ 8.48 (d, J = 8.5 Hz, 1H), 8.33 (d, J = 8.5 Hz, 1H), 8.30–8.26 (m, 3H), 8.17–8.15 (m, 3H), 8.06 (t, J = 8.0 Hz, 1H), 5.81 (d, J = 14.5 Hz, 1H); ^{13}C NMR (125 MHz, DMSO-d_6) δ□134.8, 130.8, 130.2, 129.8, 127.8 (d, J = 6.0 Hz), 127.4, 126.9, 126.6, 126.4 (d, J = 4.8 Hz), 126.0, 125.0, 124.8, 124.5, 124.0, 123.7, 67.4 (d, J = 159 Hz); ^{31}P NMR (202 MHz, DMSO-d_6) δ□18.8; HRMS (ESI): calculated for $C_{17}H_{12}O_4P$ ([M – H]$^-$) requires m/z = 311.0473, found 311.0474. *One carbon signal is missing due to signal overlap.

1.7: Synthesis of diethyl (naphthalen-2-ylmethyl)phosphonate 7

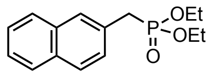

7 (KC9-11 diethyl ether)

To a solution of diethyl phosphite (0.46 mL, 3.54 mmol) in DMF (19 mL) was slowly added NaH (153 mg, 3.82 mmol) at 0 ºC. After being stirred for 30 min at 0 ºC, 2-(bromomethyl)naphthalene (500 mg, 2.26 mmol) was slowly added. The mixture was stirred for 30 minutes at 0 ºC, after which the reaction mixture was allowed to warm up to room temperature and further stirred for 60 minutes. The reaction mixture was then quenched with sat. NH_4Cl aq. and extracted with EtOAc. The organic layer was washed with water, dried over Na_2SO_4, filtrated, and concentrated *in vacuo*. The residue was applied for column chromatography (Biotage Sfär D column, 10–100% EtOAc/Hexane over 10 column volumes) to afford **7** as a colorless oil (543 mg, 86%); ^{1}H NMR (500 MHz, $CDCl_3$) δ□7.82–7.79 (m, 3H), 7.75 (s, 1H), 7.48–7.43 (m, 3H), 4.06–3.97 (m, 4H), 3.32 (d, *J* = 21.5 Hz, 2H), 1.24 (t, *J* = 7.5 Hz, 6H); ^{13}C NMR (125 MHz, $CDCl_3$) δ□133.6 (d, *J* = 3.5 Hz), 132.5 (d, *J* = 2.4 Hz), 129.3 (d, *J* = 9.5 Hz), 128.6 (d, *J* = 8.3 Hz), 128.3 (d, *J* = 6.0 Hz), 128.0 (d, *J* = 6.0 Hz), 127.7, 127.7, 126.3, 125.9, 62.3 (d, *J* = 7.1 Hz), 34.6 (d, *J* = 137 Hz), 16.5 (d, *J* = 5.9 Hz); ^{31}P NMR (202 MHz, $CDCl_3$) δ□26.9; HRMS (ESI): calculated for $C_{15}H_{20}O_3P$ ($[M + H]^+$) requires *m/z* = 279.1150, found 279.1148.

1.8: Synthesis of (naphthalen-2-ylmethyl)phosphonic acid 8

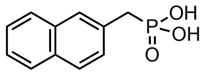

8 (KC9-11)

TMSBr (0.76 mL, 5.85 mmol) was slowly added to **7** (150 mg, 0.501 mmol) under solvent-free conditions at room temperature. After being stirred for 20 hours at room temperature under N_2, the remaining TMSBr was removed in vacuo. This residue was dissolved in MeOH (5.6 mL) and refluxed for 22 hours under N_2, after which volatile compounds were removed *in vacuo*. The resulting solid was filtrated and washed with cold acetone to afford **8** as a white solid (268 mg, 62%); ^{1}H NMR (500 MHz, DMSO-*d$_6$*) δ□7.84–7.78 (m, 3H), 7.70 (s, 1H), 7.47–7.39 (m, 3H), 3.10 (d, *J* = 21.0 Hz, 2H); ^{13}C NMR (125 MHz, DMSO-*d$_6$*) δ□133.0 (d, *J* = 2.4 Hz), 132.0 (d, *J* = 9.5 Hz), 131.6 (d, *J* = 2.4 Hz), 128.6 (d, *J* = 4.8 Hz), 127.8 (d, *J* = 8.3 Hz), 127.5, 127.3, 126.0, 125.4, 35.6 (d, *J* = 130 Hz); ^{31}P NMR (202 MHz, DMSO-*d$_6$*) δ□21.5; HRMS (ESI): calculated for $C_{11}H_{12}O_3P$ ($[M + H]^+$) requires *m/z* = 223.0524, found 223.0521. *One carbon signal is missing due to signal overlap.

1.9: Synthesis of dimethyl (hydroxy(naphthalen-2-yl)methyl)phosphonate 9

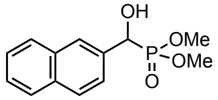

9 (KC9-A_1)

To a suspension of 2-naphthaldehyde (1.56 g, 10.0 mmol) in dimethyl phosphite (1.8 mL, 20.0 mmol) was added Na_2CO_3 (2.12 g, 20.0 mmol) under solvent-free conditions at room temperature. The mixture was stirred for 24 hours at room temperature, after which the reaction mixture was diluted with EtOAc and washed with water. The organic layer was dried over Na_2SO_4, filtrated, and concentrated *in vacuo*. The residue was applied for column chromatography (Biotage Sfär D column, 0–8% $CH_3OH/CHCl_3$ over 10 column volumes) to afford **9** as a white solid (2.17 g, 82%); ^{1}H NMR (500 MHz, $CDCl_3$) δ□7.96 (s, 1H), 7.87–7.84 (m, 3H), 7.65–7.63 (m, 1H), 7.50–7.45 (m, 2H), 5.15 (d, *J* = 13.0 Hz, 1H), 3.71 (d, *J* = 10.0 Hz, 3H); ^{13}C NMR (125 MHz, $CDCl_3$) δ□133.8 (d, *J* = 2.4 Hz), 133.4, 133.3 (d, *J* = 2.5 Hz), 128.3, 128.3, 127.8, 126.4, 126.2 (d, *J* = 8.3 Hz), 124.8 (d, *J* = 4.8 Hz), 71.0 (d, *J* = 159 Hz), 54.1 (d, *J* = 7.1 Hz), 53.9 (d, *J* = 7.1 Hz); ^{31}P NMR (125 MHz, $CDCl_3$) δ□24.0; HRMS (ESI): calculated for $C_{13}H_{15}O_4NaP$ ($[M + Na]^+$) requires *m/z* = 289.0606, found 289.0604. *One carbon signal is missing due to signal overlap.

1.10: Synthesis of monomethyl (hydroxy(naphthalen-2-yl)methyl)phosphonic acid 10

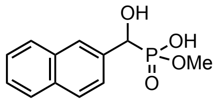

10 (KC9-A)

TMSCl (23.7 μL, 0.188 mmol) was added to a mixture of **9** (200 mg, 0.751 mmol) and NaI (28.0 mg, 0.187 mmol) in CH_3CN (7.5 mL) at room temperature. The reaction mixture was filtrated and washed with H_2O. The reaction mixture was quenched with MeOH and concentrated in vacuo. The residue was applied for column chromatography (Biotage Sfär C18 Duo column, 5–45% CH_3CN/H_2O over 12 column volumes) to afford **10** as a white solid (11.6 mg, 6%); ^{1}H NMR (500 MHz, CD_3OD) δ□7.97 (s, 1H), 7.85–7.82 (m, 3H), 7.60–7.58 (m, 1H), 7.50–7.47 (m, 2H), 5.22 (d, *J* = 13.0 Hz, 1H), 3.71 (d, *J* = 10.0 Hz, 3H); ^{13}C NMR (125 MHz, CD_3OD) δ□136.8, 134.6, 129.0, 128.6, 127.4 (d, *J* = 7.1 Hz), 127.1, 127.0, 126.4 (d, *J* = 4.8 Hz), 71.7 (d, *J* = 162 Hz), 53.8 (d, *J* = 7.1 Hz); ^{31}P NMR (125 MHz, CD_3OD) δ□22.7; HRMS (ESI): calculated for $C_{12}H_{12}O_4P$ ($[M - H]^-$) requires *m/z* = 251.0473, found 251.0475. *Two carbon signals are missing due to signal overlap.

Scheme 2. NMR spectra

2.1a: ^{1}H NMR (500 MHz, $CDCl_3$)

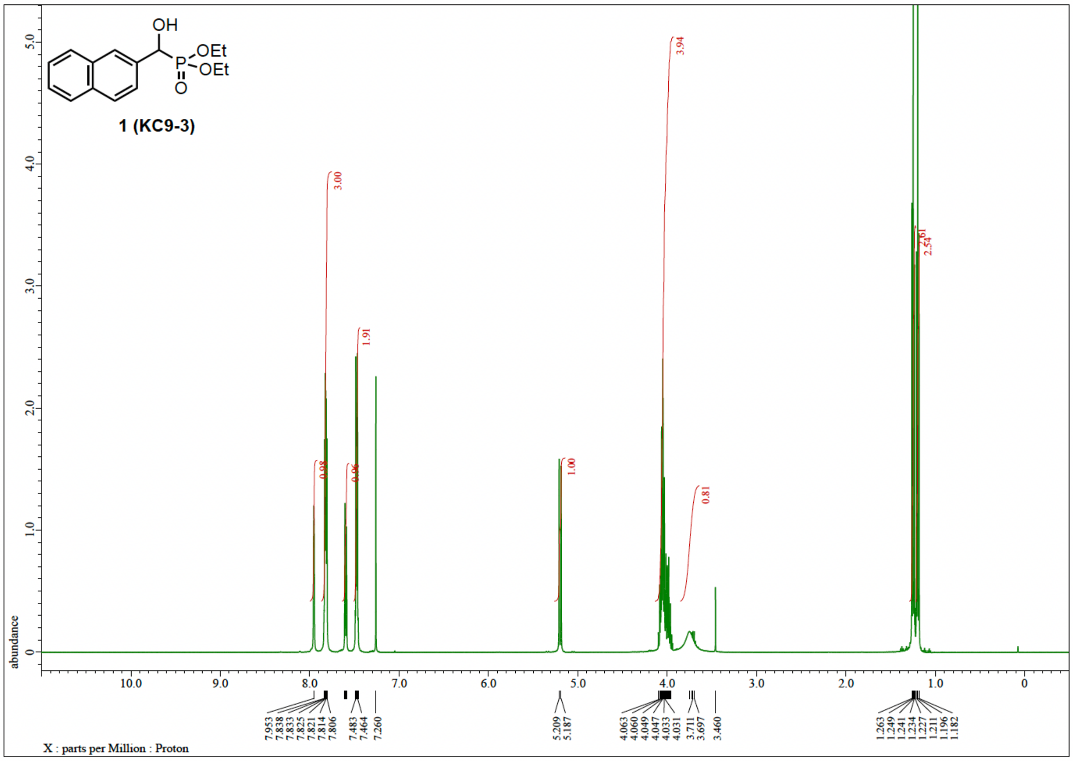

2.1b: ^{13}C NMR (125 MHz, $CDCl_3$)

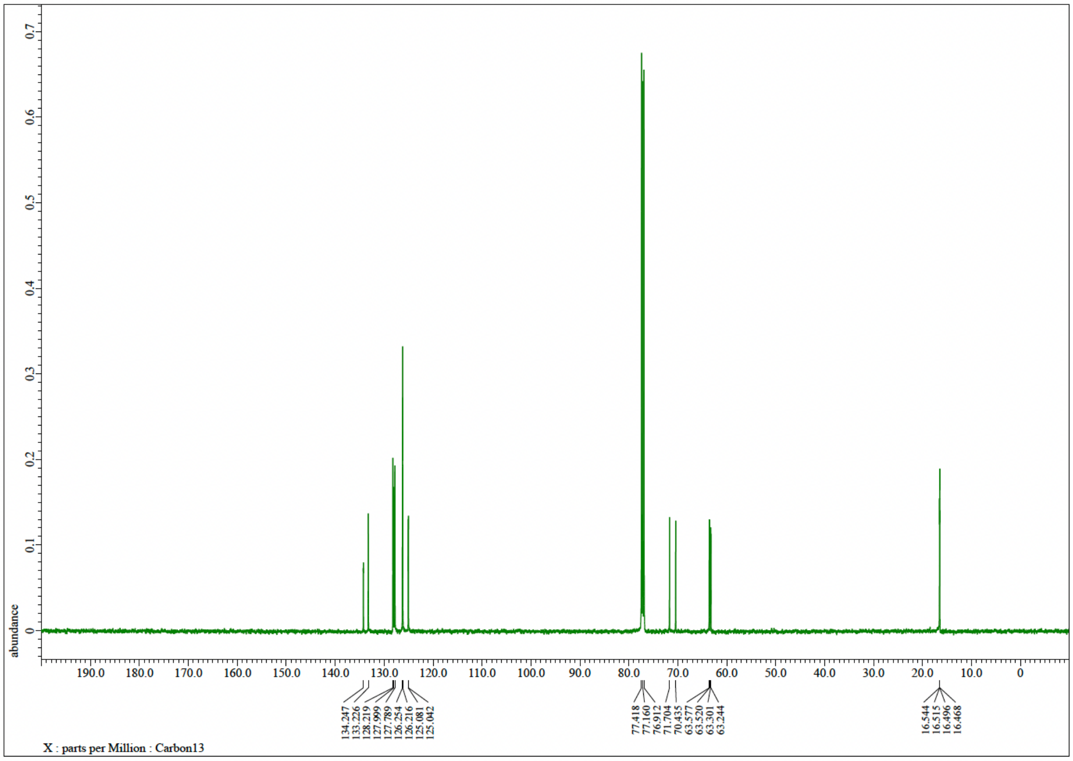

2.1c: ^{31}P NMR (202 MHz, $CDCl_3$)

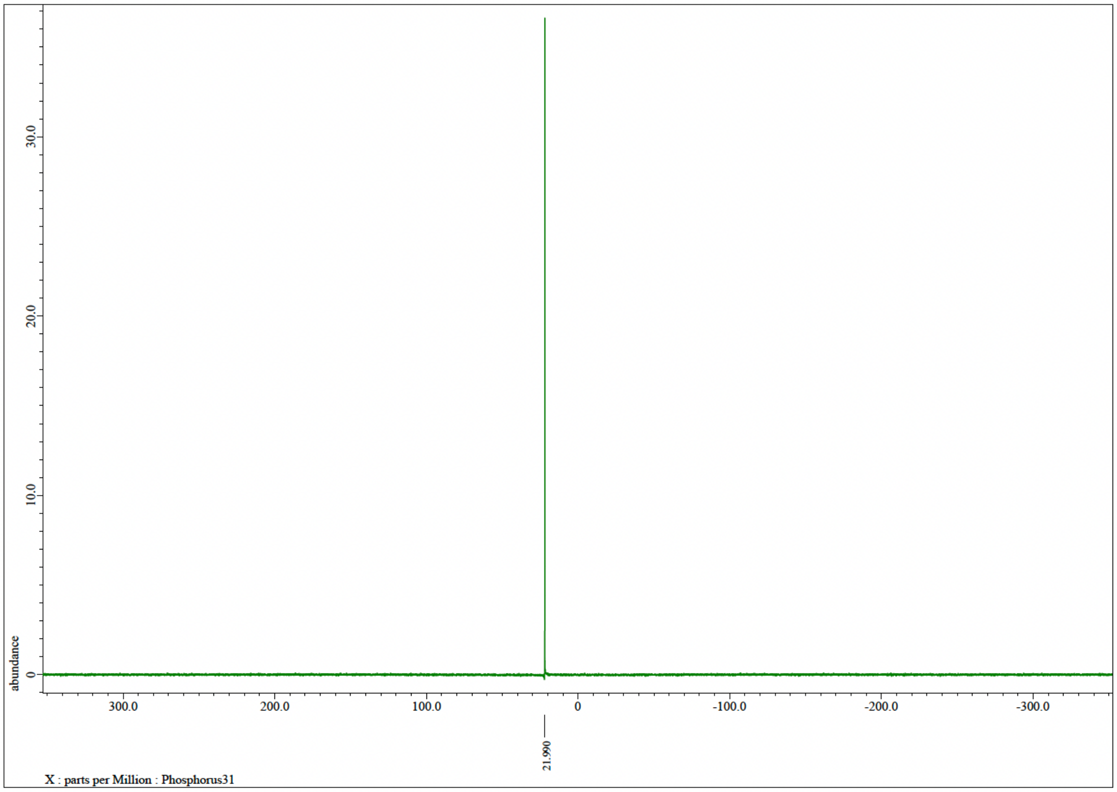

2.2a: ^{1}H NMR (500 MHz, DMSO-d_6)

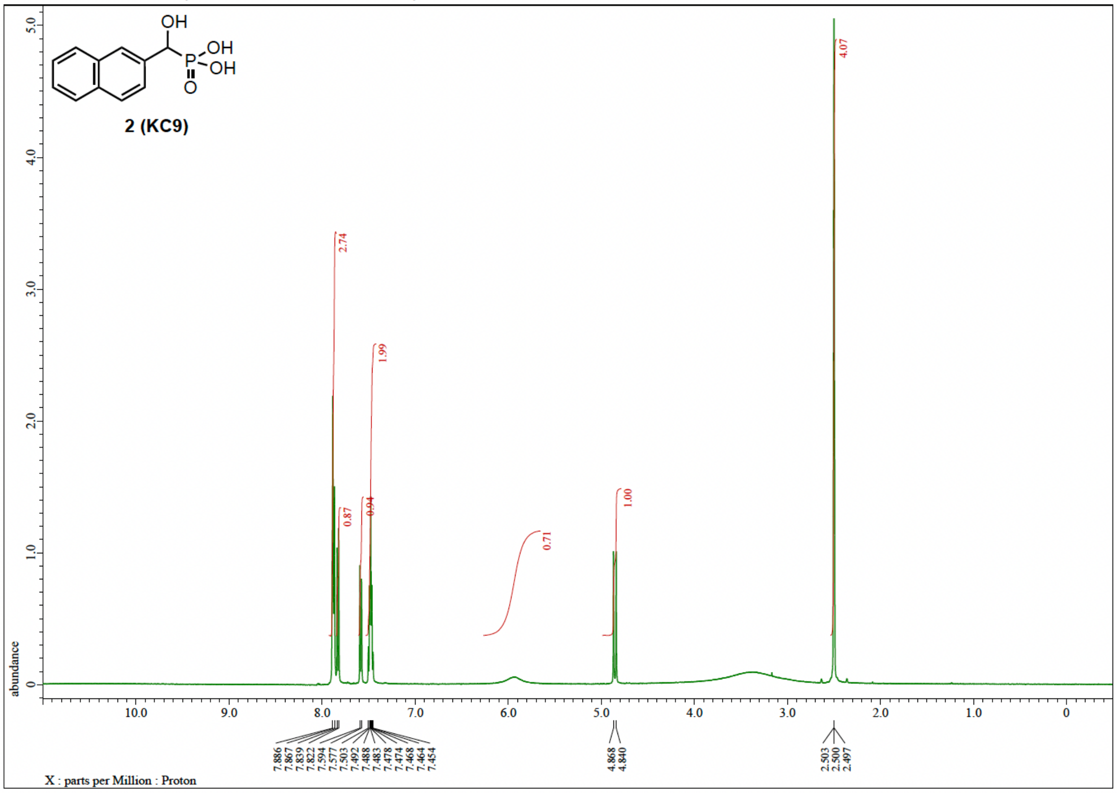

2.2b: ^{13}C NMR (125 MHz, DMSO-d_6)

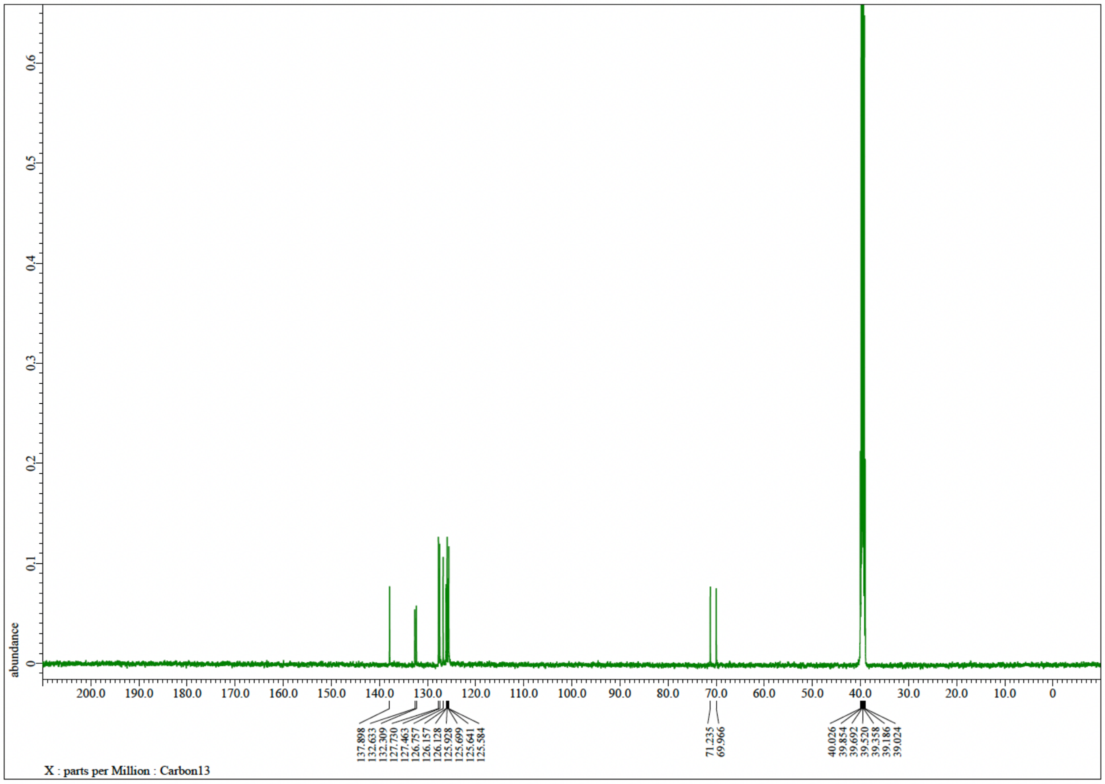

2.2c: ^{31}P NMR (202 MHz, DMSO-d_6)

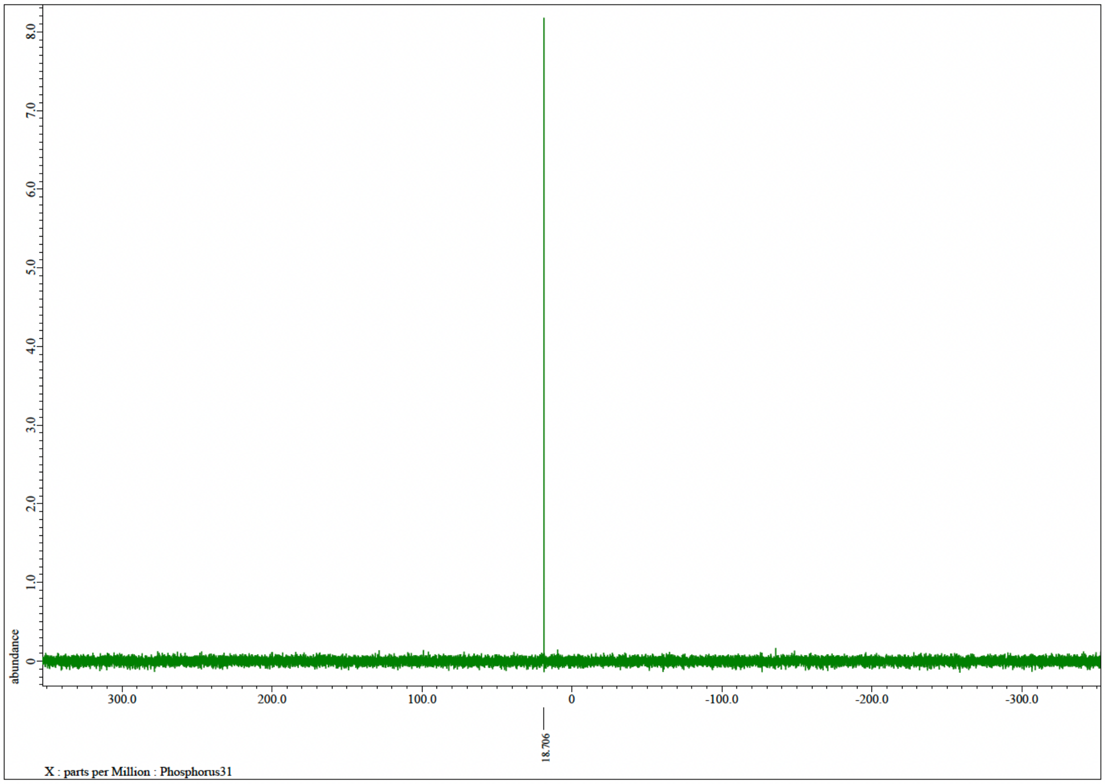

2.3a: ^{1}H NMR (500 MHz, $CDCl_3$)

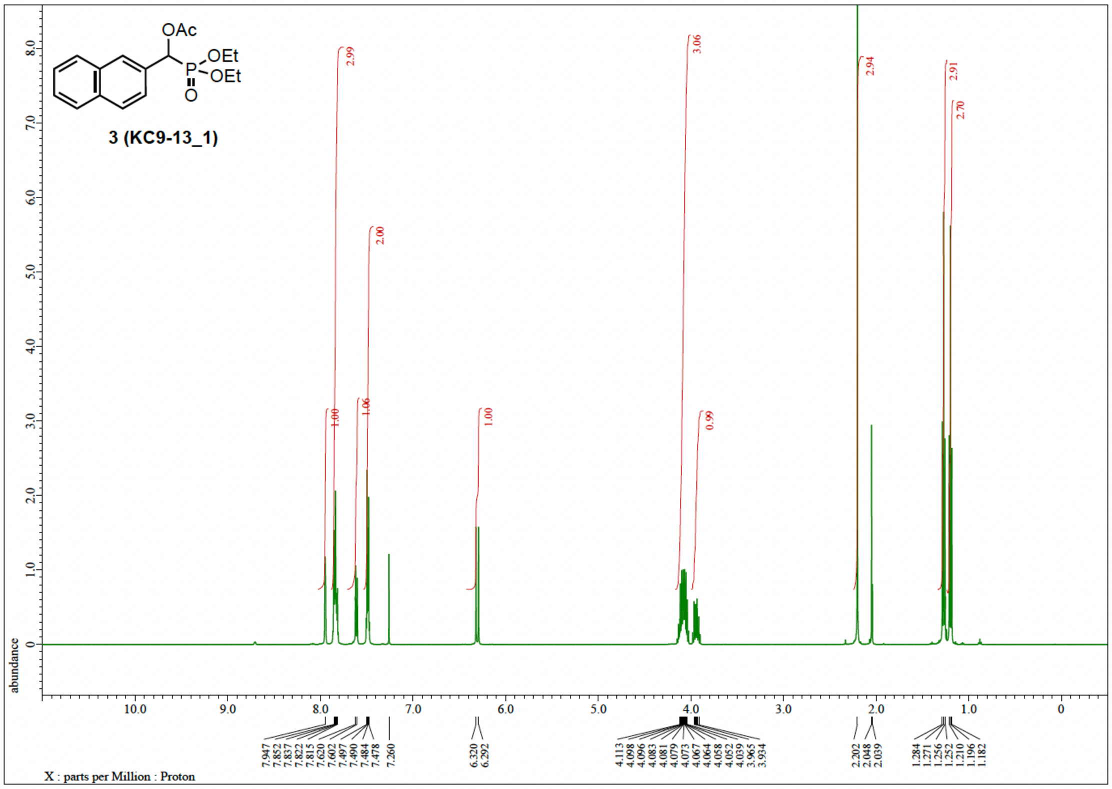

2.3b: ^{13}C NMR (125 MHz, $CDCl_3$)

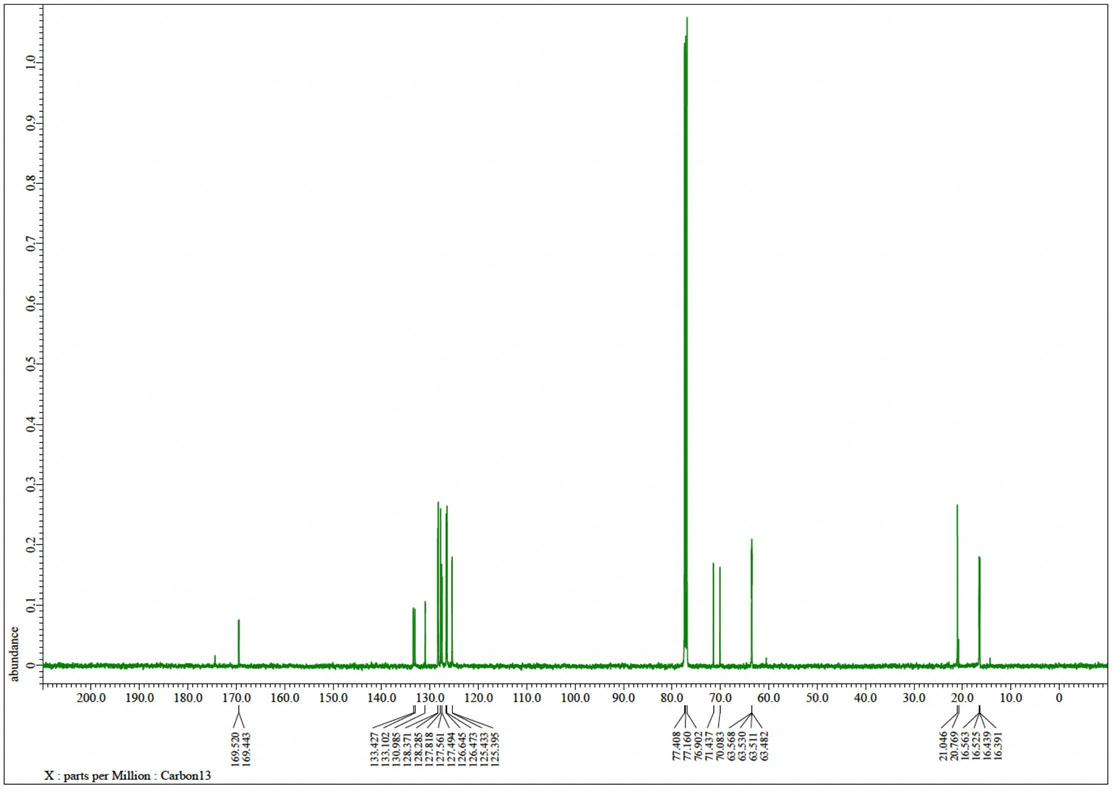

2.3c: ^{31}P NMR (202 MHz, $CDCl_3$)

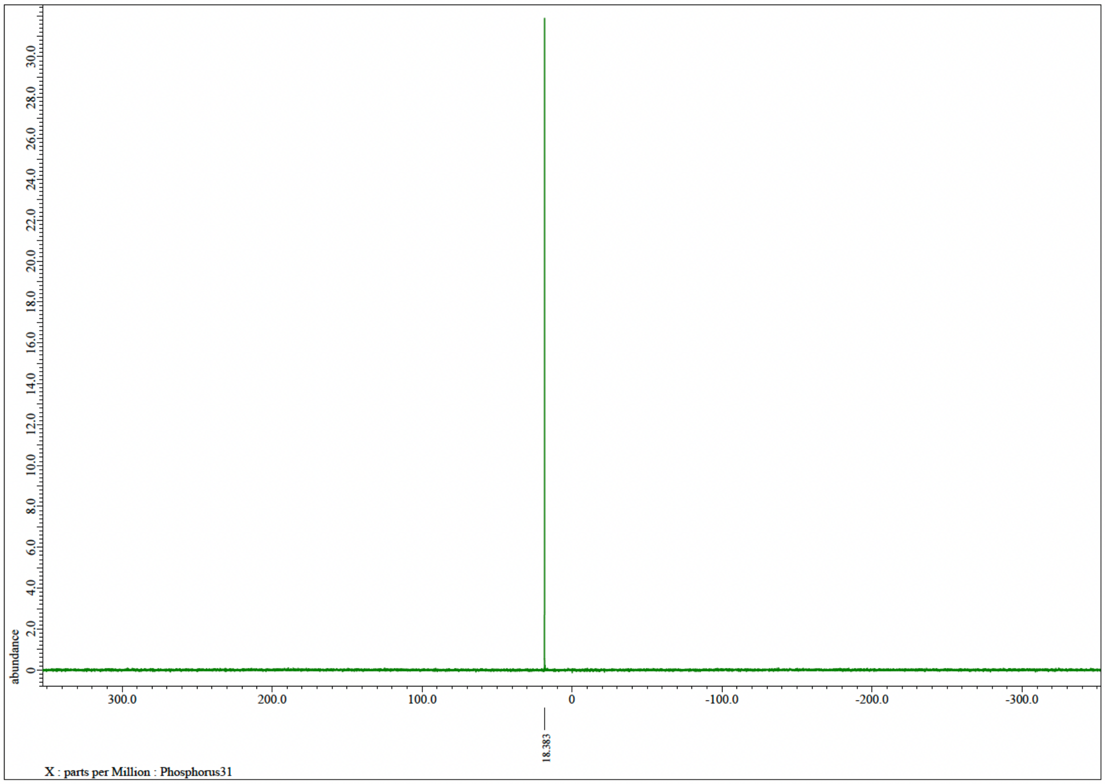

2.4a: ^{1}H NMR (500 MHz, DMSO-d_6)

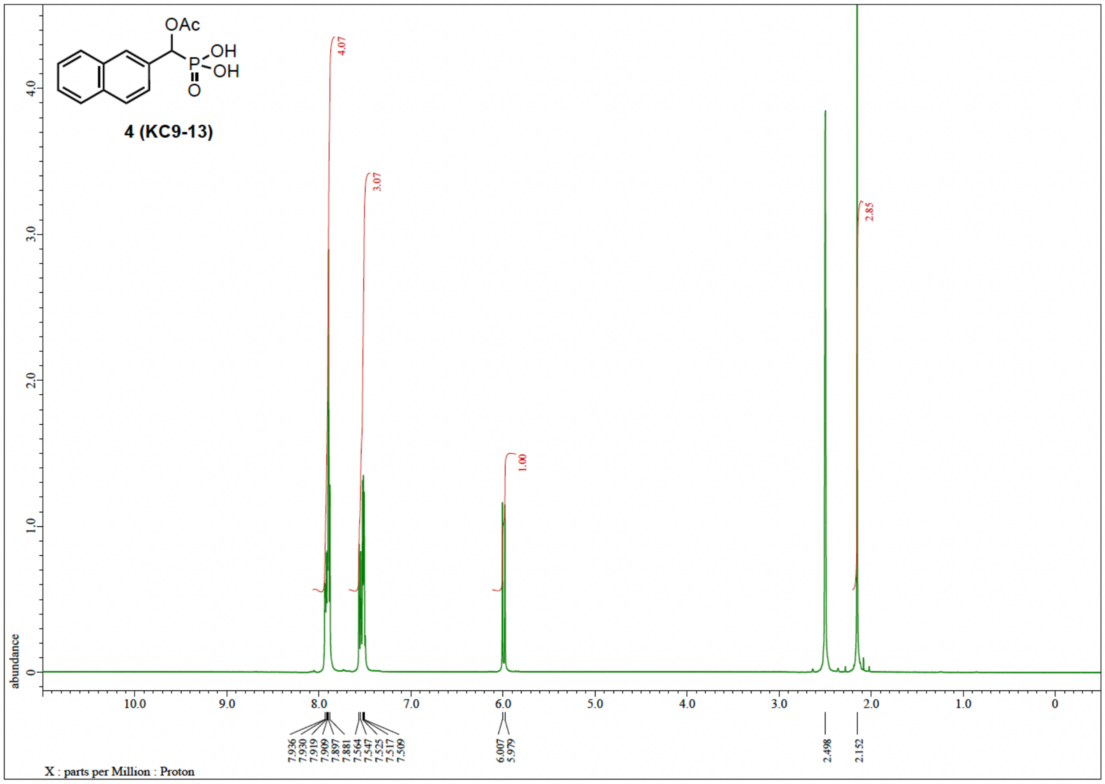

2.4b: ^{13}C NMR (125 MHz, DMSO-d_6)

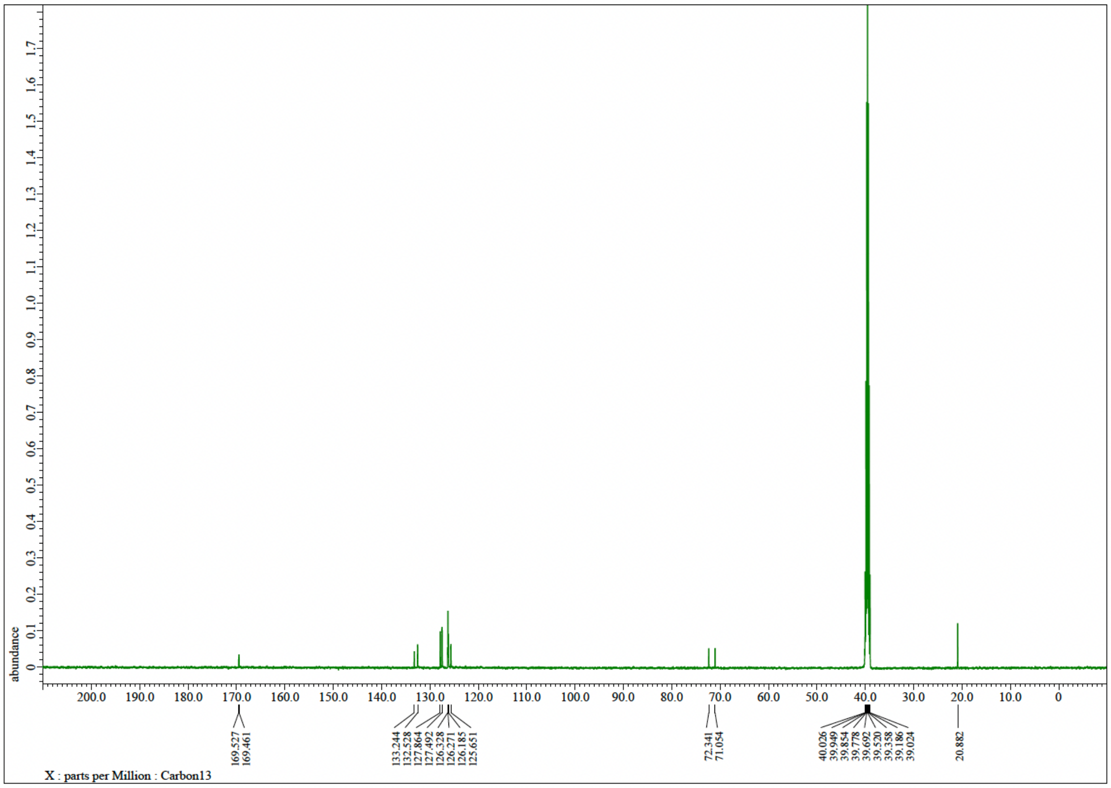

2.4c: ^{31}P NMR (202 MHz, DMSO-d_6)

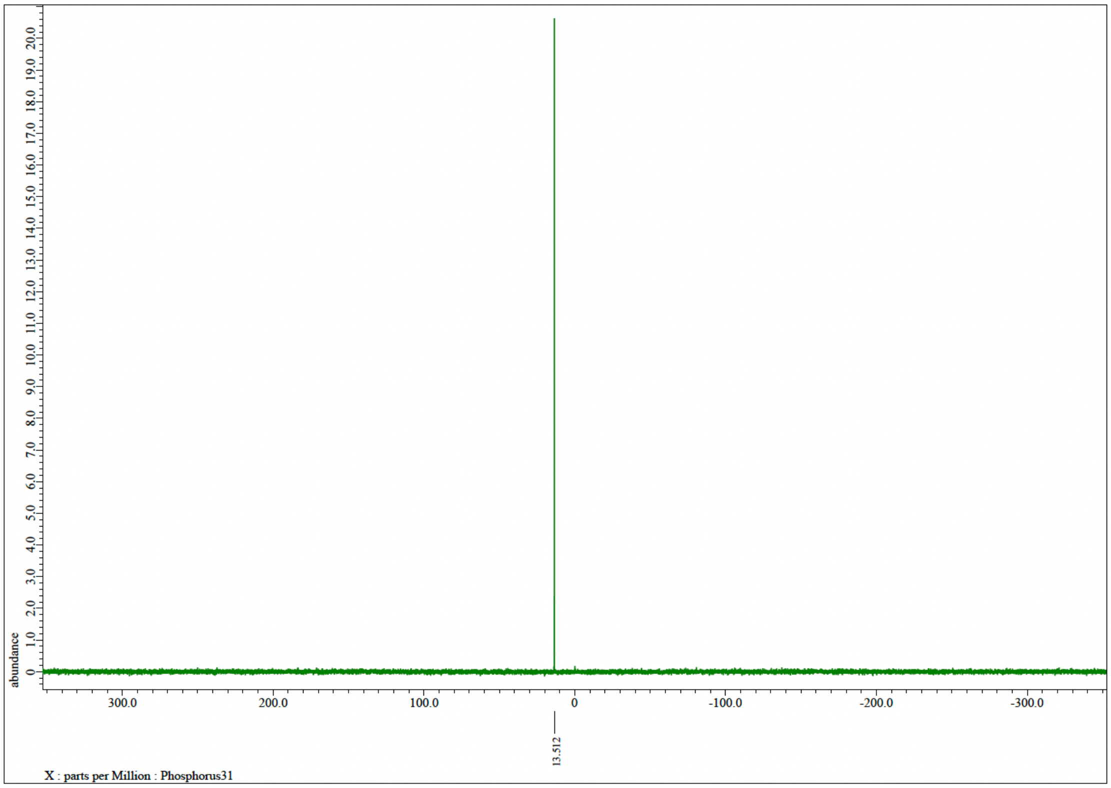

2.5a: ^{1}H NMR (500 MHz, DMSO-d_6)

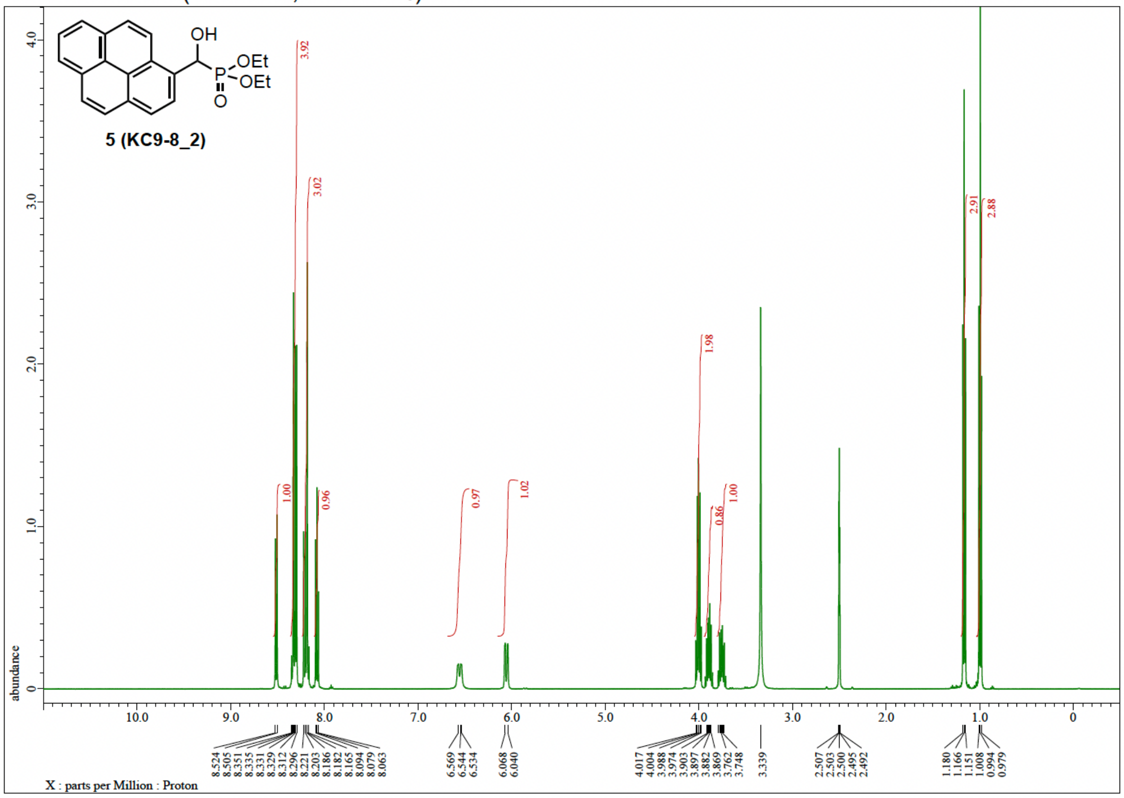

2.5b: ^{13}C NMR (125 MHz, DMSO-d_6)

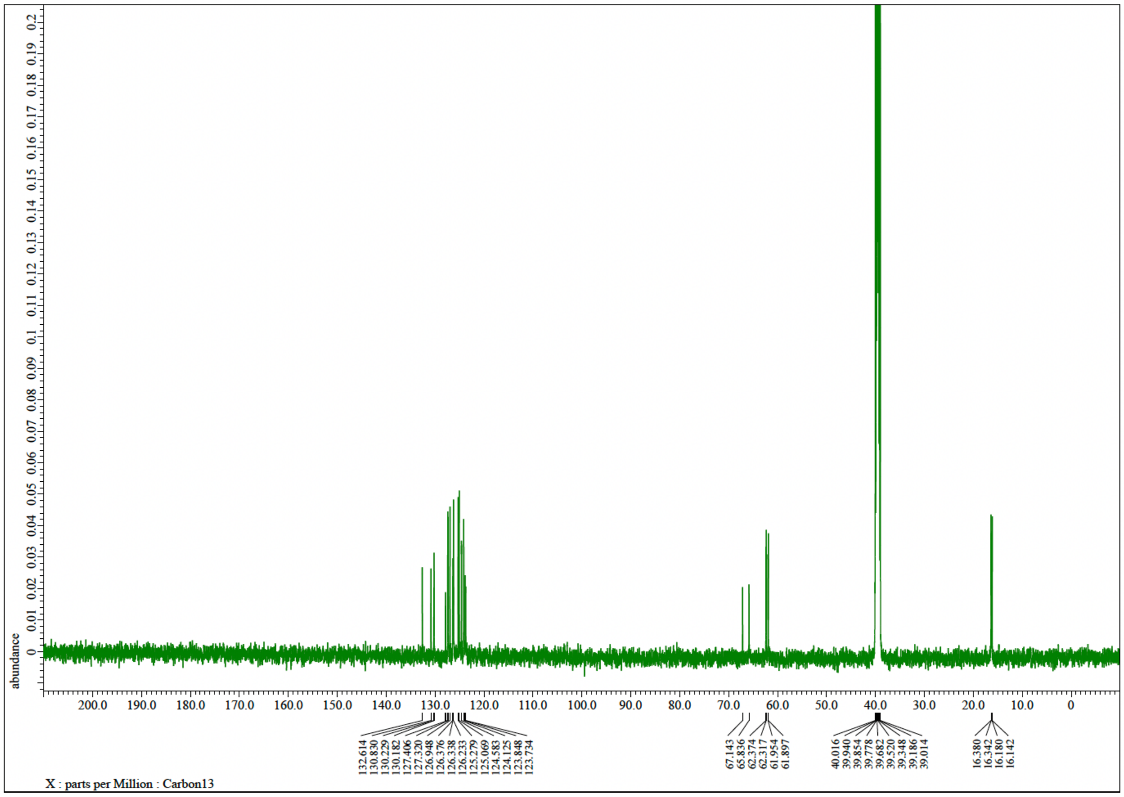

2.5c: ^{31}P NMR (202 MHz, DMSO-d_6)

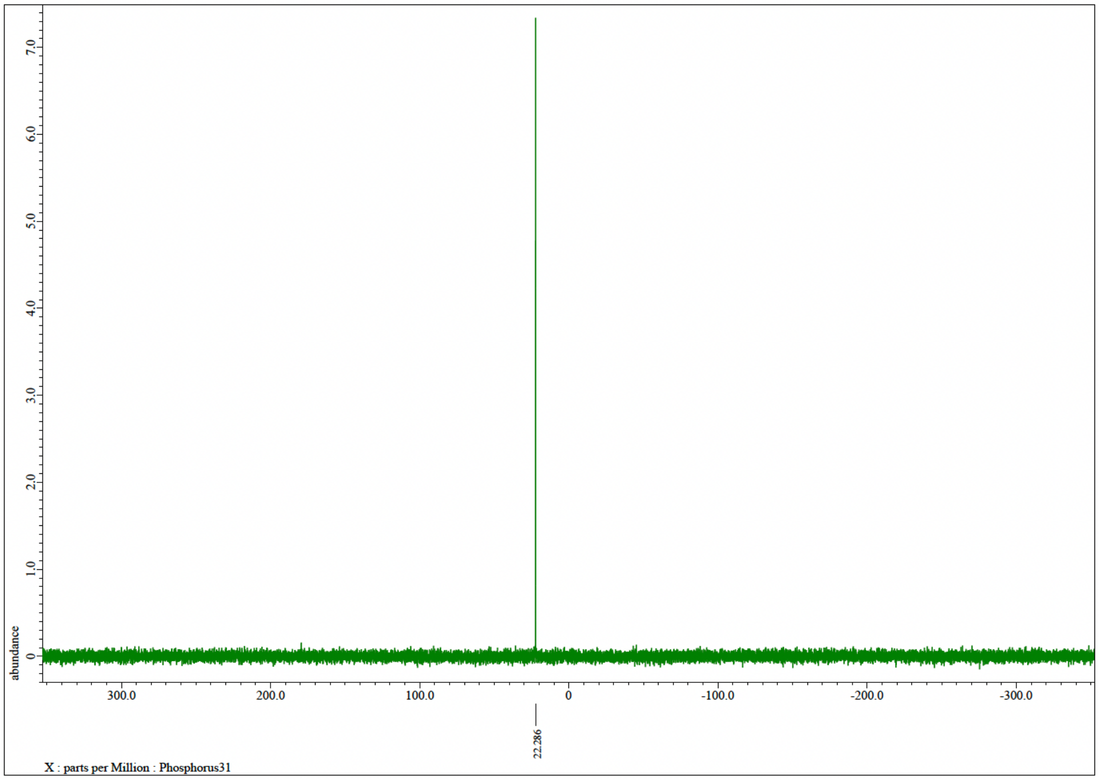

2.6a: ^{1}H NMR (500 MHz, DMSO-d_6)

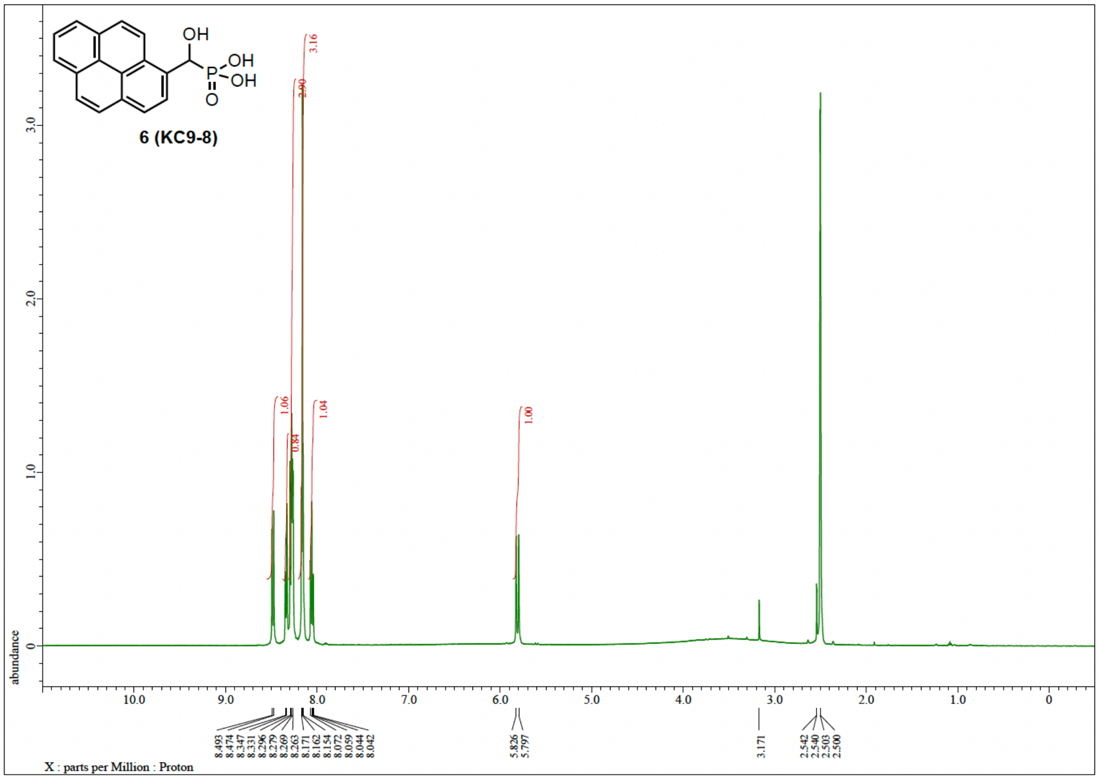

2.6b: ^{13}C NMR (125 MHz, DMSO-d_6)

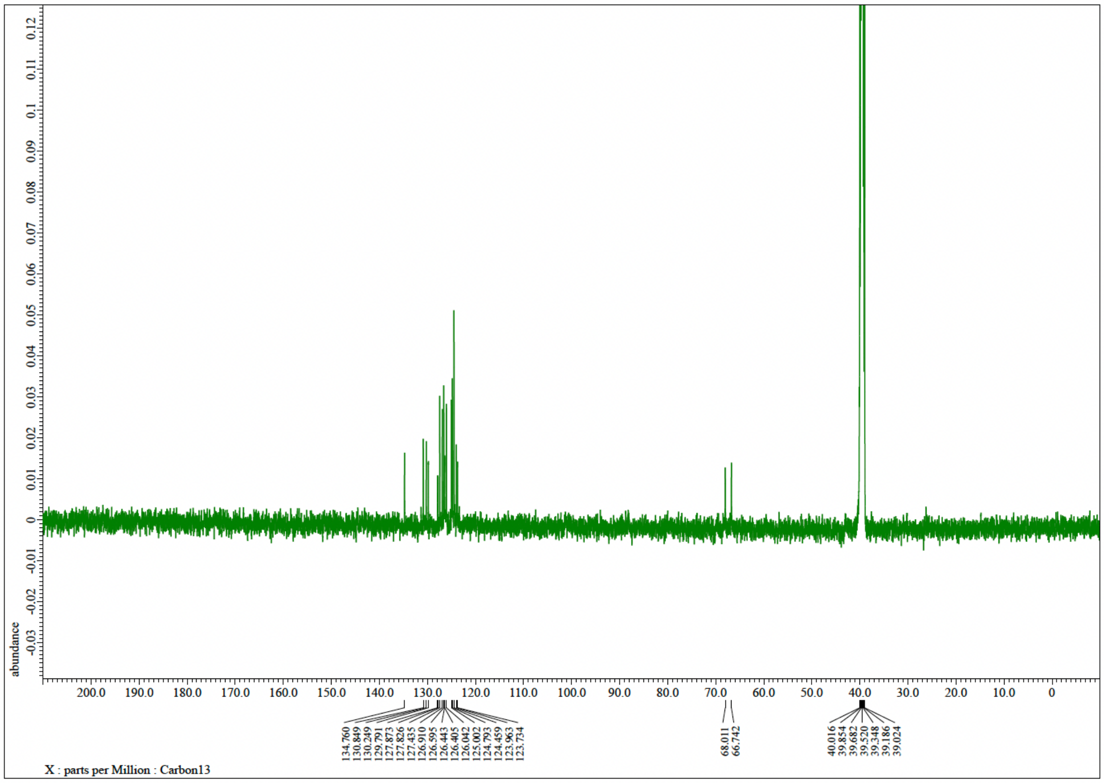

2.6c: ^{31}P NMR (202 MHz, DMSO-d_6)

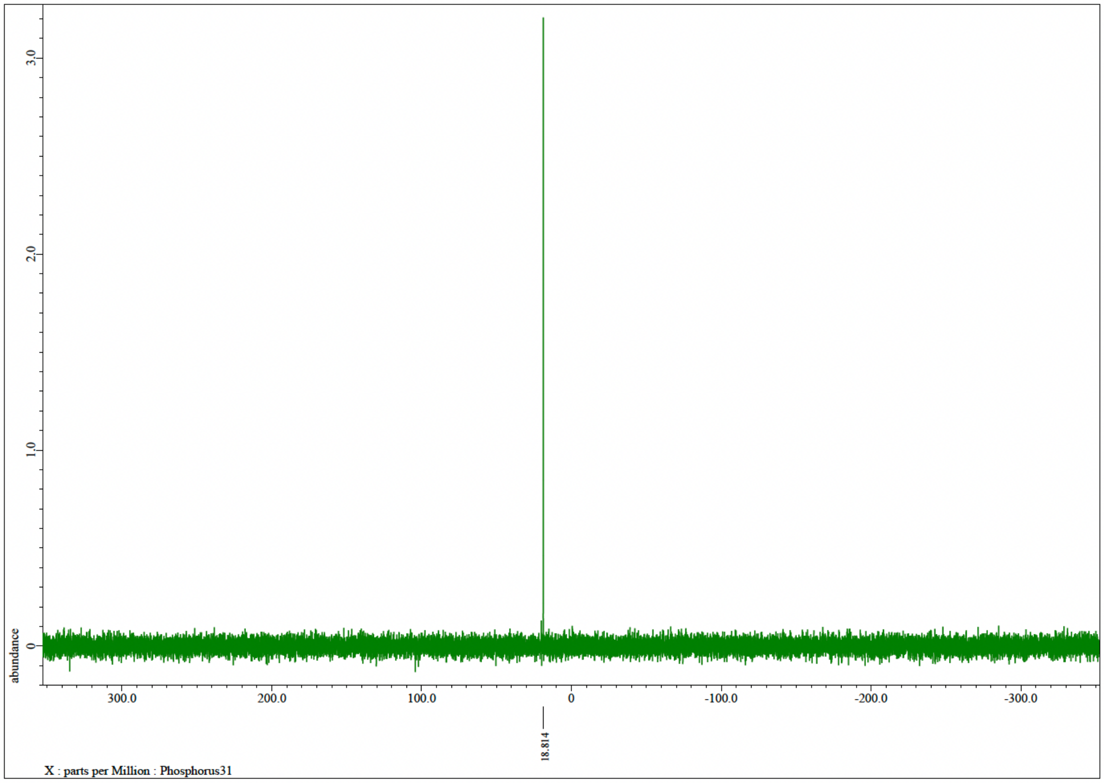

7: ^{1}H NMR (500 MHz, $CDCl_3$)

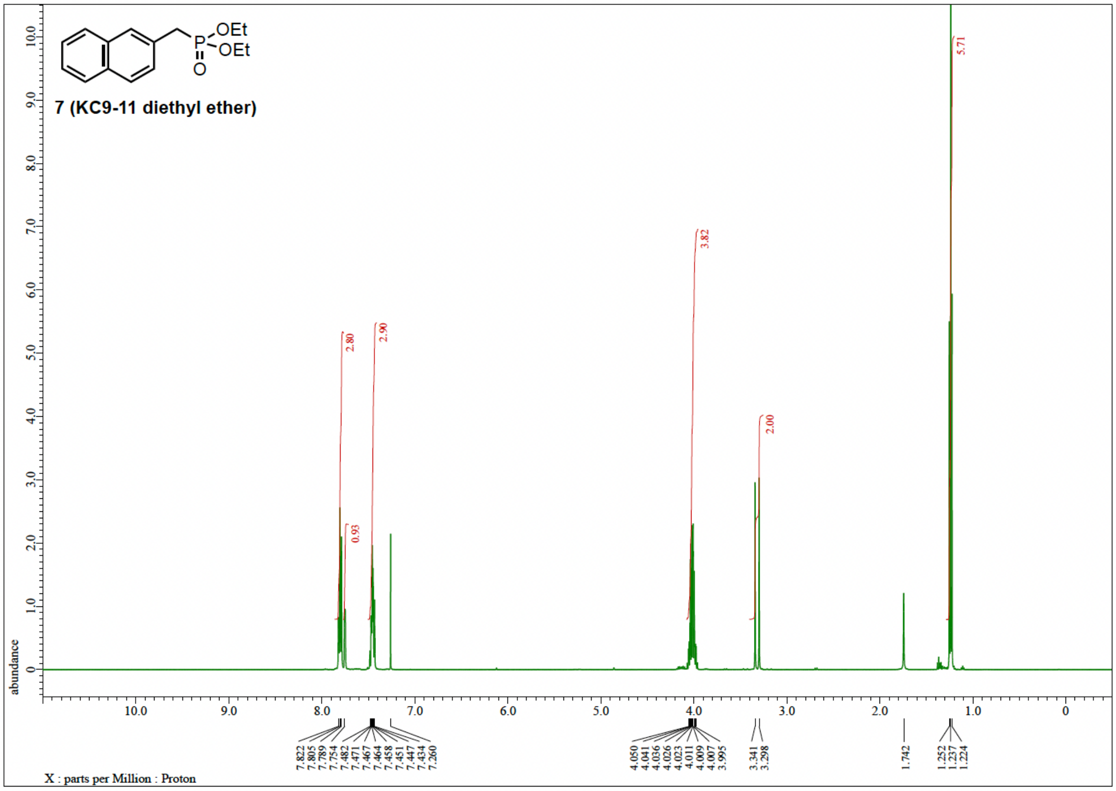

7: ^{13}C NMR (125 MHz, $CDCl_3$)

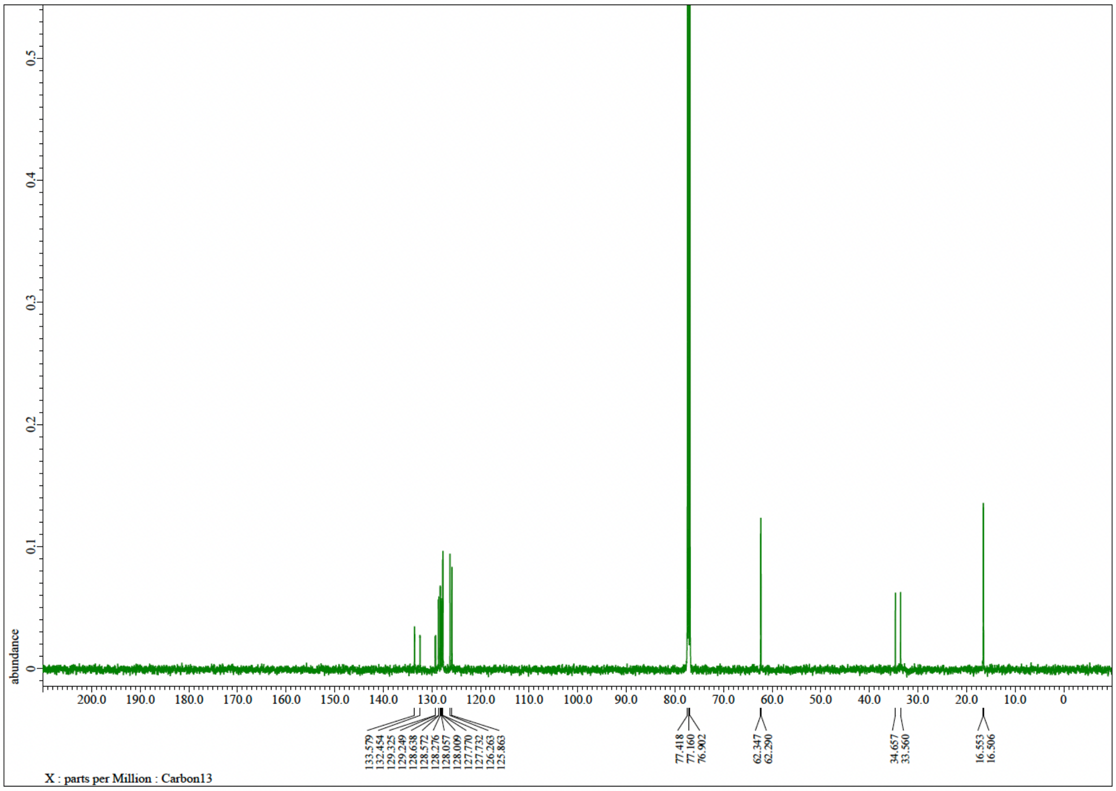

7: ^{31}P NMR (202 MHz, $CDCl_3$)

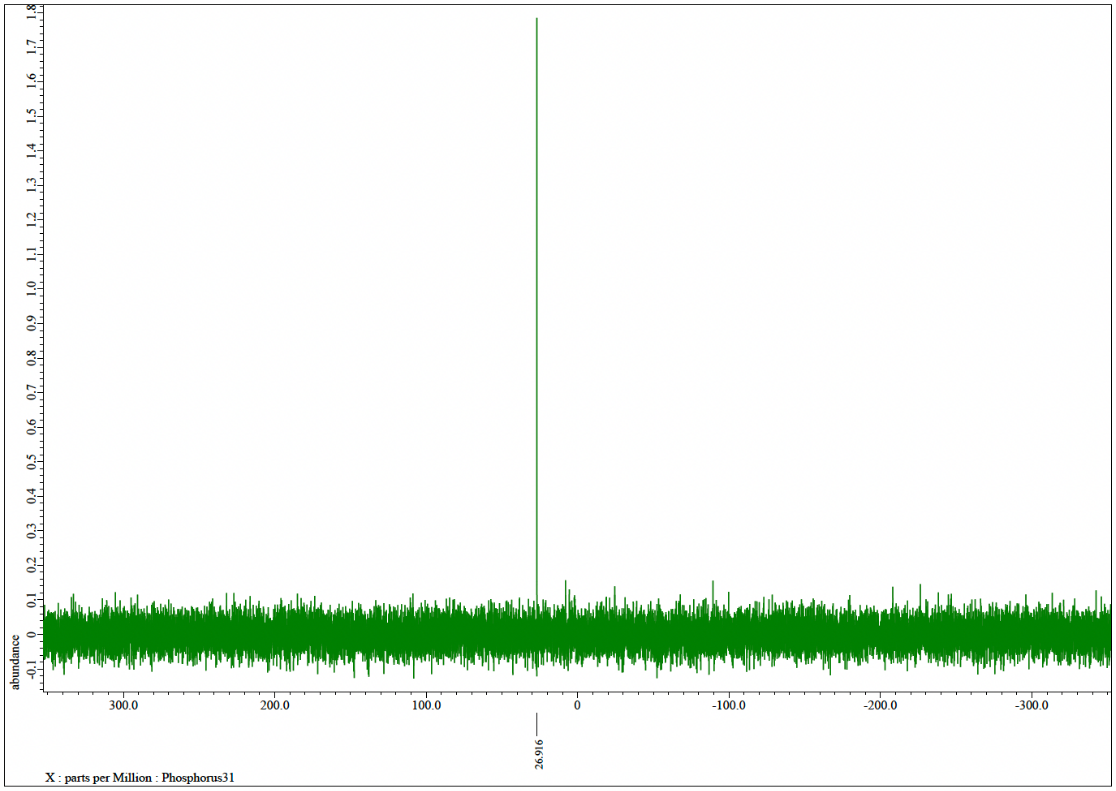

8: ^{1}H NMR (500 MHz, DMSO-d_6)

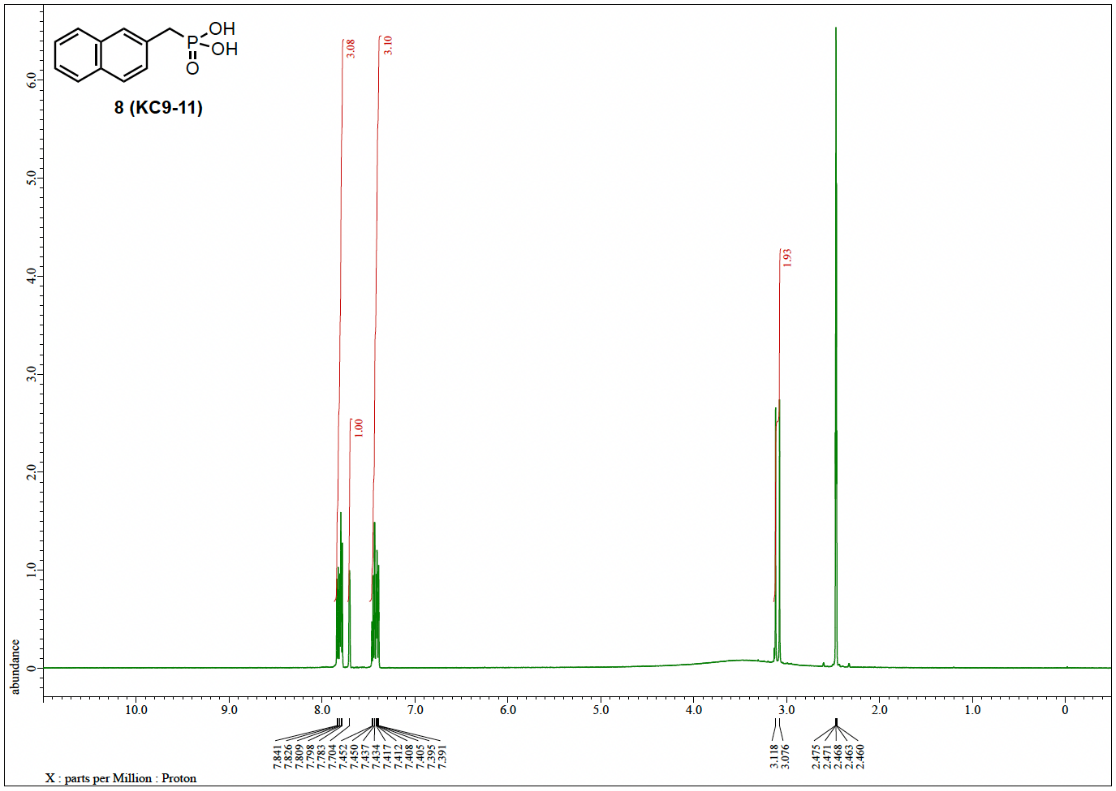

8: ^{13}C NMR (125 MHz, DMSO-d_6)

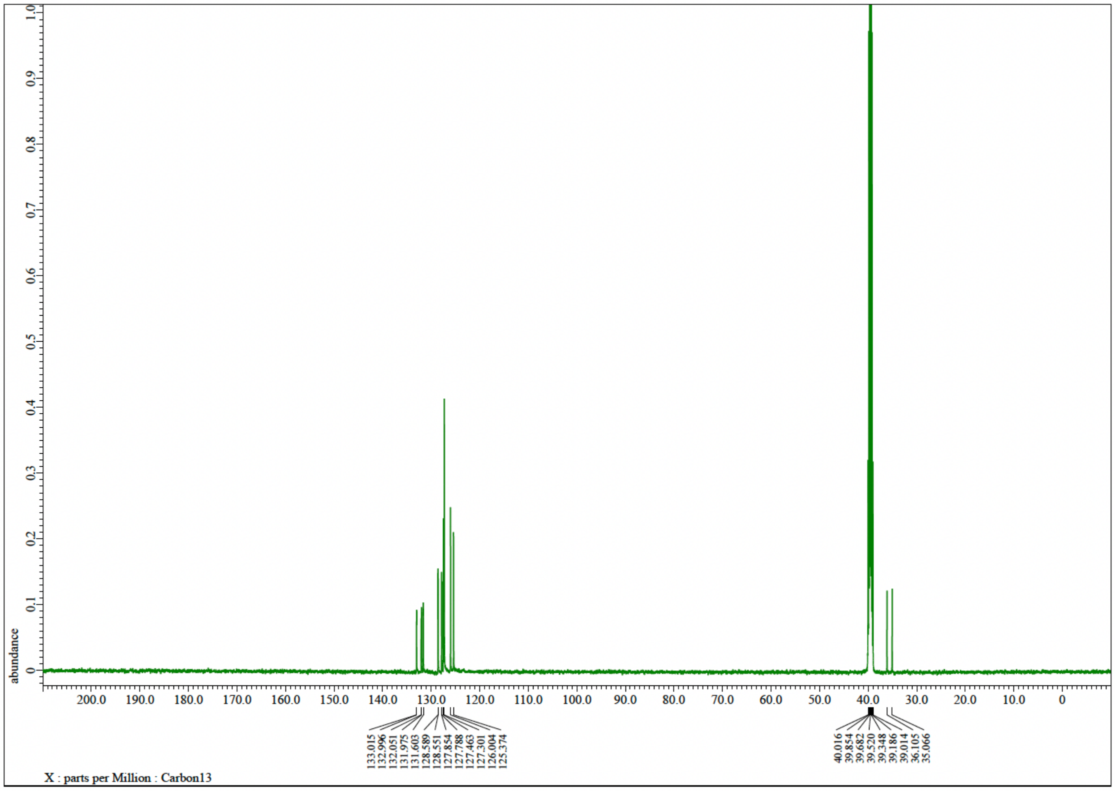

8: ^{31}P NMR (202 MHz, DMSO-d_6)

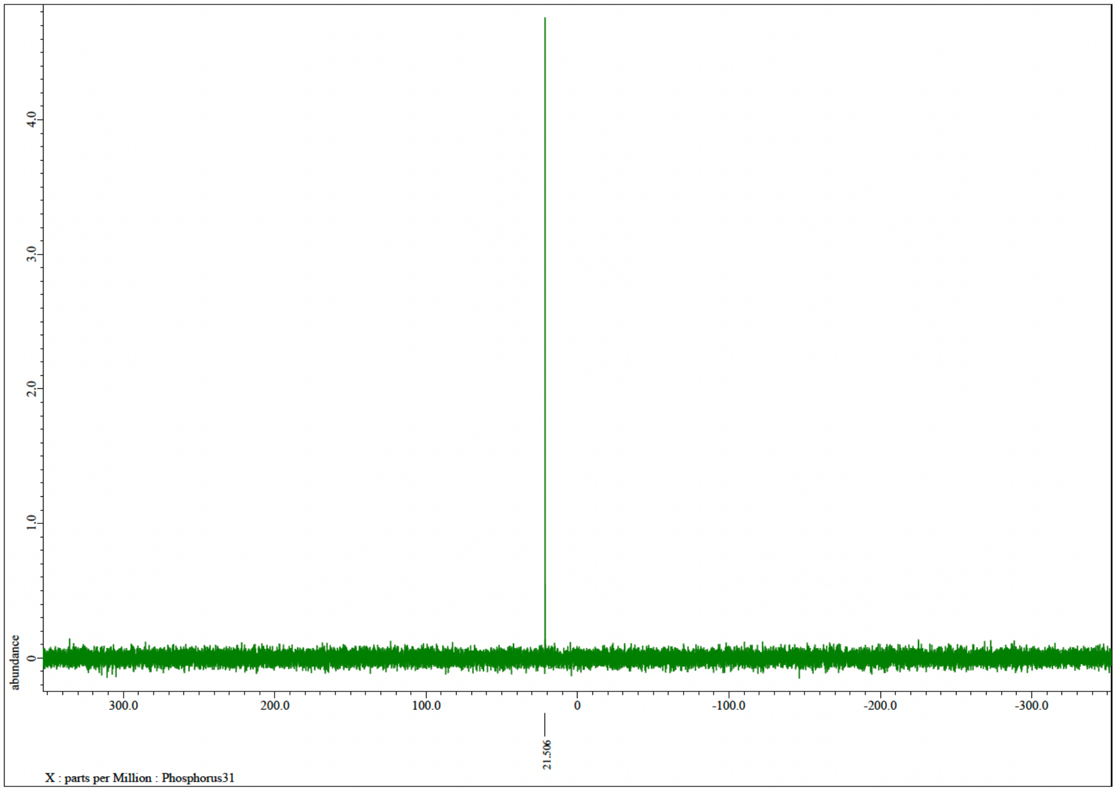

9: ^{1}H NMR (500 MHz, $CDCl_3$)

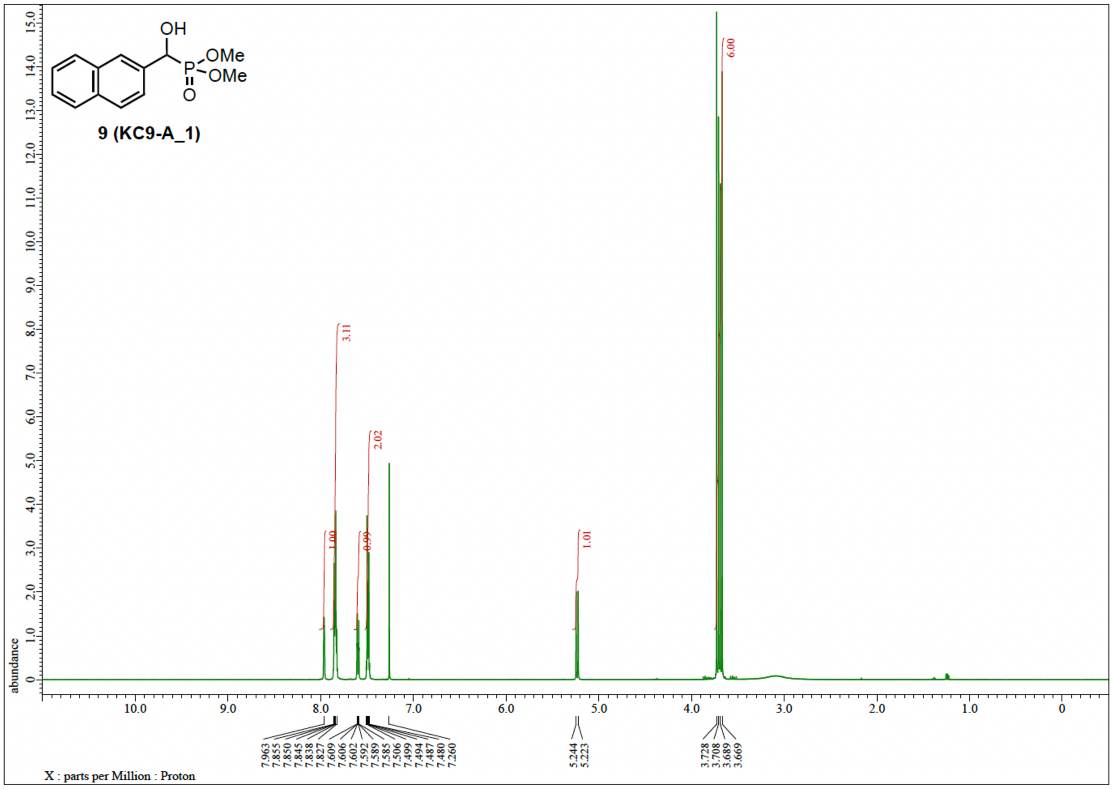

9: ^{13}C NMR (125 MHz, $CDCl_3$)

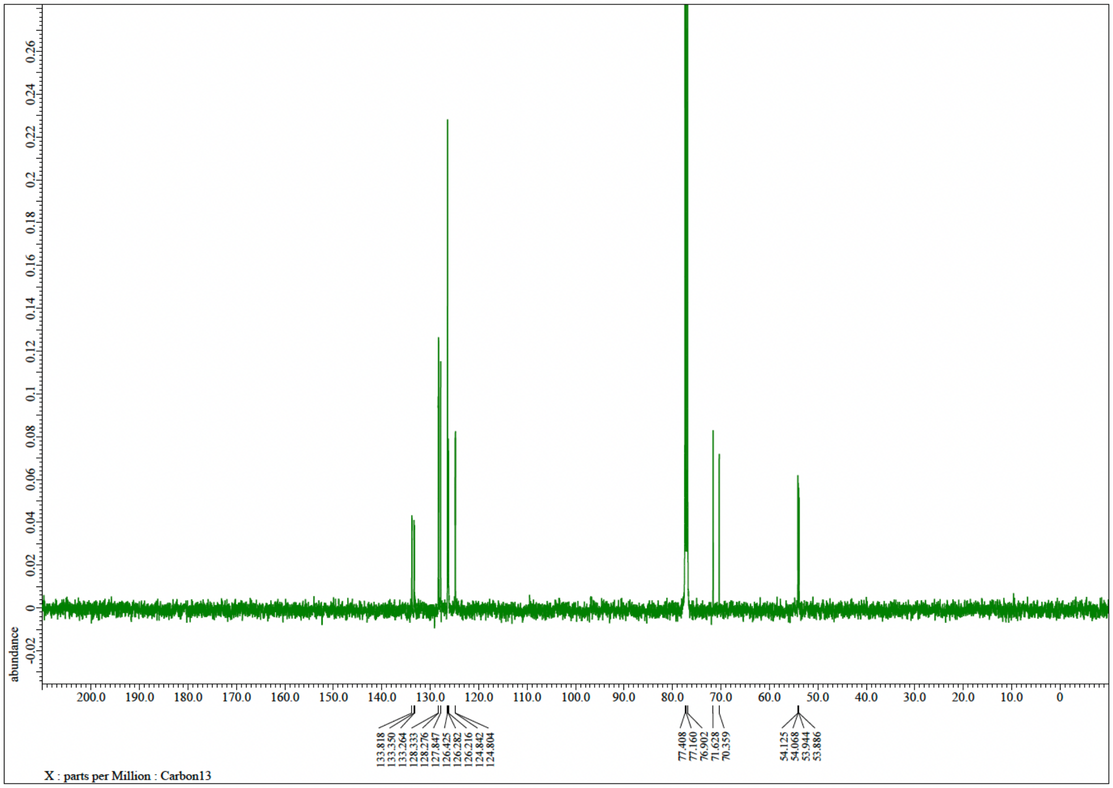

9: ^{31}P NMR (202 MHz, $CDCl_3$)

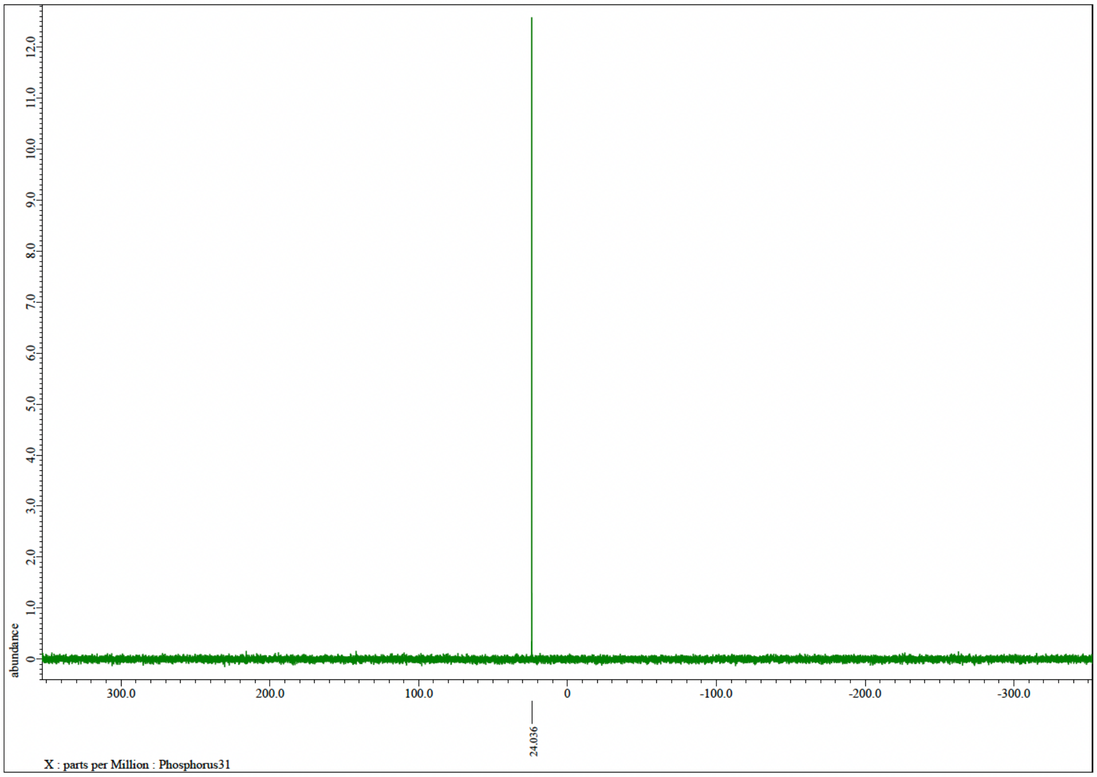

10: ^{1}H NMR (500 MHz, CD_3OD)

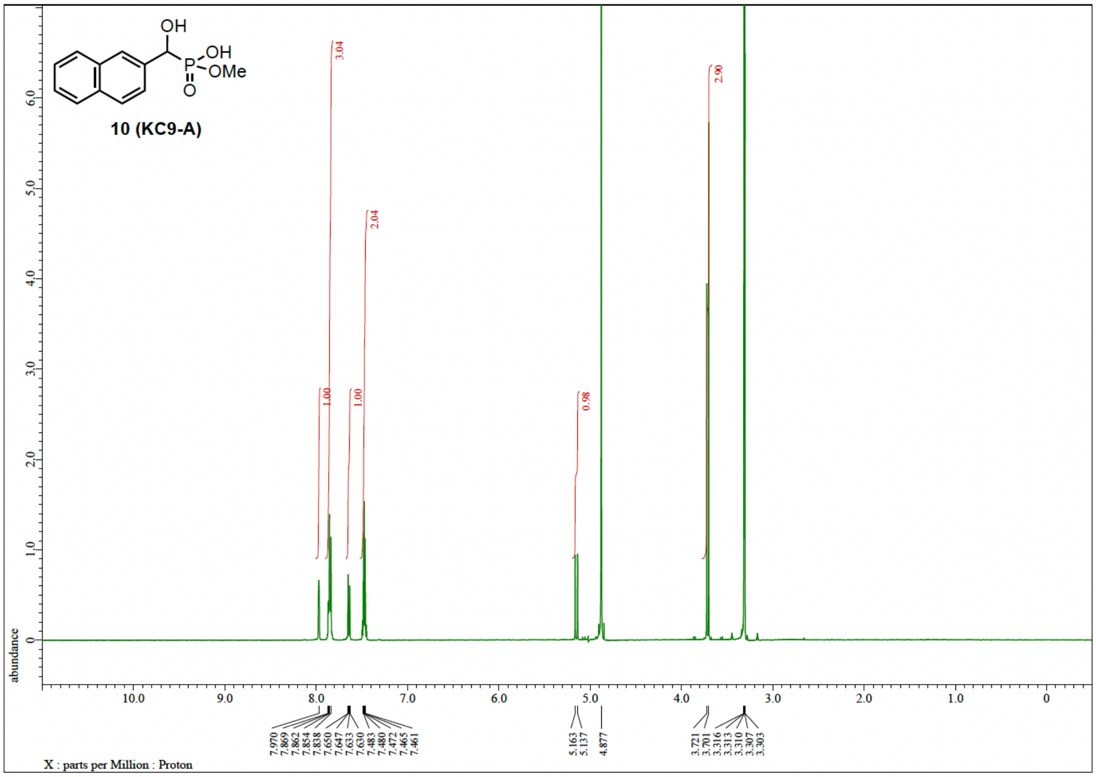

10: ^{13}C NMR (125 MHz, CD_3OD)

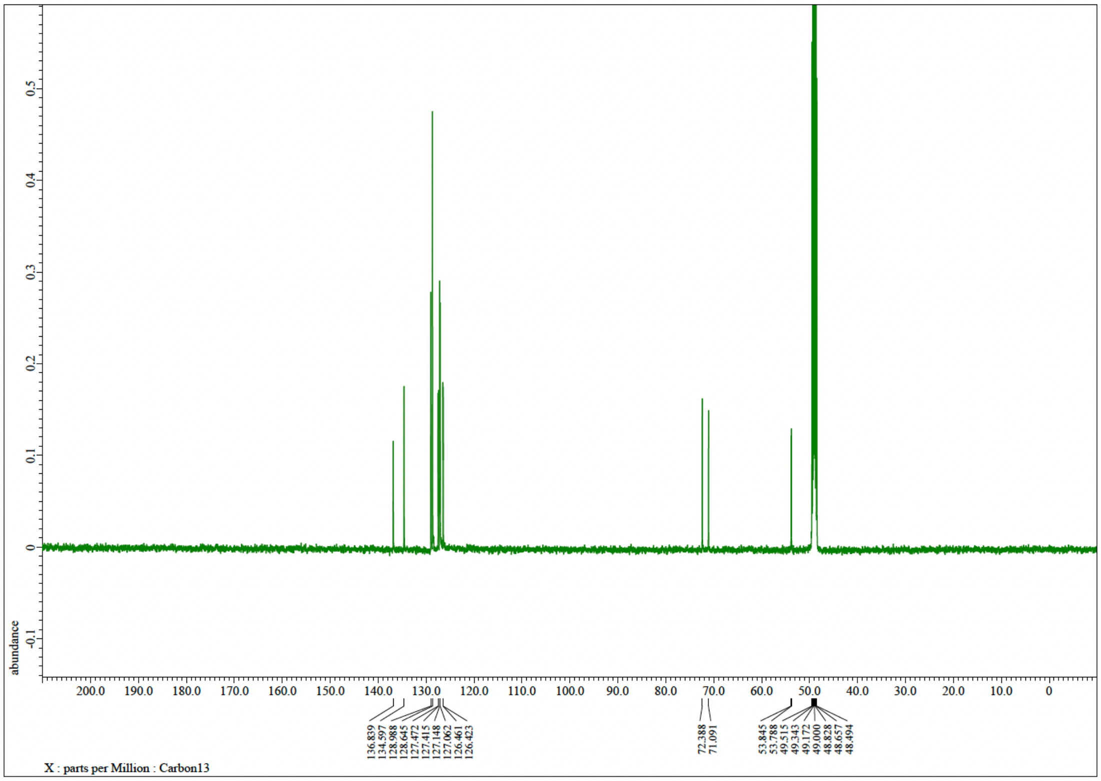

10: ^{31}P NMR (202 MHz, CD_3OD)

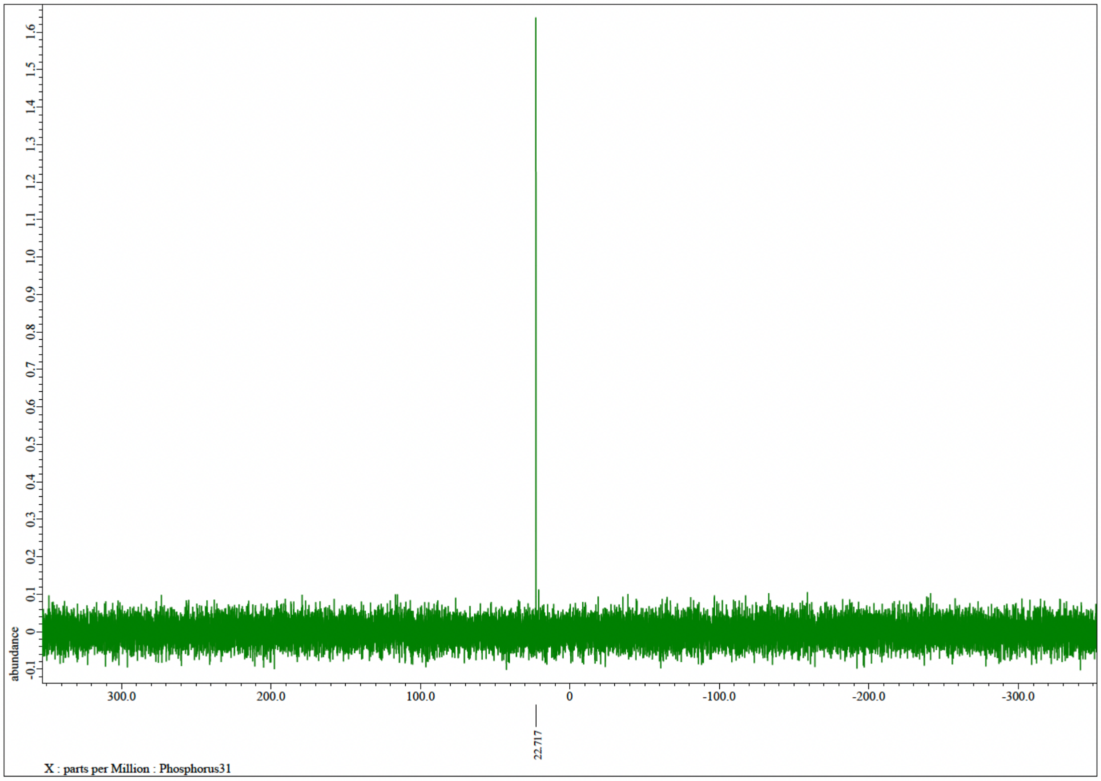

Data S1. (Separate file) Quantification of stomatal phenotypes and p values

Data S2. (Separate file) RNA-seq read statistics, DESs, and SOM Cluster GO categories
